# Adult neural stem cells and neurogenesis are resilient to intermittent fasting

**DOI:** 10.15252/embr.202357268

**Published:** 2023-11-21

**Authors:** Rut Gabarró‐Solanas, Amarbayasgalan Davaatseren, Justus Kleifeld, Tatjana Kepčija, Thomas Köcher, Albert Giralt, Iván Crespo‐Enríquez, Noelia Urbán

**Affiliations:** ^1^ Institute of Molecular Biotechnology of the Austrian Academy of Sciences (IMBA) Vienna BioCenter (VBC) Vienna Austria; ^2^ Vienna BioCenter PhD Program Doctoral School of the University of Vienna and Medical University of Vienna Vienna Austria; ^3^ Vienna BioCenter Core Facilities Vienna Austria; ^4^ Departament de Biomedicina, Facultat de Medicina, Institut de Neurociències Universitat de Barcelona Barcelona Spain; ^5^ Institut d'Investigacions Biomèdiques August Pi i Sunyer (IDIBAPS) Barcelona Spain; ^6^ Centro de Investigación Biomédica en Red Sobre Enfermedades Neurodegenerativas (CIBERNED) Barcelona Spain; ^7^ Production and Validation Center of Advanced Therapies (Creatio), Faculty of Medicine and Health Science University of Barcelona Barcelona Spain

**Keywords:** adult neurogenesis, dietary restriction, IF, NSCs, quiescence, Metabolism, Neuroscience, Stem Cells & Regenerative Medicine

## Abstract

Intermittent fasting (IF) is a promising strategy to counteract ageing shown to increase the number of adult‐born neurons in the dentate gyrus of mice. However, it is unclear which steps of the adult neurogenesis process are regulated by IF. The number of adult neural stem cells (NSCs) decreases with age in an activation‐dependent manner and, to counteract this loss, adult NSCs are found in a quiescent state which ensures their long‐term maintenance. We aimed to determine if and how IF affects adult NSCs in the hippocampus. To identify the effects of every‐other‐day IF on NSCs and all following steps in the neurogenic lineage, we combined fasting with lineage tracing and label retention assays. We show here that IF does not affect NSC activation or maintenance and, that contrary to previous reports, IF does not increase neurogenesis. The same results are obtained regardless of strain, sex, diet length, tamoxifen administration or new‐born neuron identification method. Our data suggest that NSCs maintain homeostasis upon IF and that this intervention is not a reliable strategy to increase adult neurogenesis.

## Graphical abstract

Combining genetic lineage tracing and label retention experiments, this study shows that every‐other‐day intermittent fasting does not affect adult neural stem cell behaviour and, despite previous encouraging data, is not a reliable strategy to promote adult hippocampal neurogenesis in mice.

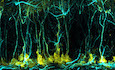

## Introduction

Adult stem cells are rare cell populations in adult tissues that generate new somatic cells to support tissue turnover and repair upon injury (Li & Clevers, 2010). With age, stem cell pools decline and the function of the remaining stem cells is impaired (López‐Otín *et al*, 2013; Goodell & Rando, 2015; Schultz & Sinclair, 2016; Tümpel & Rudolph, 2019). Fasting, either in the form of intermittent fasting or time‐restricted feeding, is a dietary intervention that prolongs life and health span (Longo & Panda, 2016; Madeo *et al*, 2019). The health benefits of fasting are explained, in part, by improved stem cell function throughout the body, including in the intestine, muscle or haematopoietic system (Novak *et al*, 2021).

The adult mammalian brain harbours neural stem cells (NSCs) in specific locations such as the dentate gyrus (DG) of the hippocampus (Kuhn *et al*, 2018). There, NSCs are the source of adult‐born granule neurons that integrate into the pre‐existing hippocampal circuit offering an additional layer of plasticity that is crucial for the modulation of memory, learning, mood or emotions (Bond *et al*, 2015). NSCs in the DG have a limited ability to self‐renew and are mostly found in an inactive, quiescent state that prevents their activity‐coupled exhaustion (Bonaguidi *et al*, 2011; Encinas *et al*, 2011; Urbán *et al*, 2016; Pilz *et al*, 2018; Bottes *et al*, 2021). Interestingly, NSCs can return to quiescence after proliferation, a mechanism that helps preserve the active NSC pool and sustain neurogenesis throughout life (Urbán *et al*, 2016; Bottes *et al*, 2021). Yet, the aged brain contains fewer NSCs which, in addition, shift to a deeper quiescent state resulting in a decrease in adult neurogenesis (Harris *et al*, 2021). Understanding NSC quiescence is therefore crucial to elucidate the mechanisms that control and support neurogenesis during adulthood and ageing.

Fasting is widely regarded as an efficient strategy to increase adult neurogenesis, therefore holding great potential to improve cognitive ability and prevent the age‐related neurogenic decline (Mattson, 2003; Levenson & Rich, 2007; Zainuddin & Thuret, 2012; Fusco & Pani, 2013; Pani, 2015; Van Cauwenberghe *et al*, 2016; Wahl *et al*, 2016; Poulose *et al*, 2017; Landry & Huang, 2021). However, we still do not understand how NSCs and other cells along the neurogenic lineage respond to fasting. To generate new neurons, quiescent NSCs first activate and give rise to intermediate progenitor cells (IPCs). These are highly proliferative cells that amplify the progenitor pool and further differentiate into neuroblasts, eventually maturing into functional granule neurons. Fasting has been reported to promote the survival of newly born cells (Lee *et al*, 2002a,b; Kitamura *et al*, 2006; Brandhorst *et al*, 2015; Kim *et al*, 2015), with some works also reporting an increase in proliferation in the DG (Park & Lee, 2011; Brandhorst *et al*, 2015; Dias *et al*, 2021; Cao *et al*, 2022). It is therefore not clear whether fasting increases the neuronal output of adult NSCs by promoting maturation and survival of newly born neurons, by increasing proliferation of IPCs and/or by activating quiescent NSCs. A fasting‐induced burst in NSC proliferation could lead to the exhaustion of the NSC pool eventually impairing adult neurogenesis. Determining the stages of the neurogenic lineage at which fasting acts to increase adult neurogenesis and whether it affects NSC activation and maintenance is necessary to predict its long‐term consequences.

Here, we have established an intermittent fasting (IF) protocol that effectively causes systemic changes associated with the benefits of fasting without altering the activity pattern of mice. To study the effects of IF on NSCs, we used genetic lineage tracing and thymidine analogues in combination with immunofluorescence. IF did not affect NSC proliferation or maintenance even after a 4‐month‐long intervention. This could make IF an ideal strategy to increase adult neurogenesis without compromising the stem cell pool. However, we also found that IF did not increase adult neurogenesis at any given time. We investigated experimental variables that could explain why our results did not match previous reports on adult neurogenesis, such as mouse strain, sex, diet length, tamoxifen administration or newly born neuron quantification method. Our results consistently show that adult neurogenesis does not increase upon IF.

## Results

### Intermittent fasting with daytime refeeding disrupts the circadian activity pattern of mice

**Figure 1:**
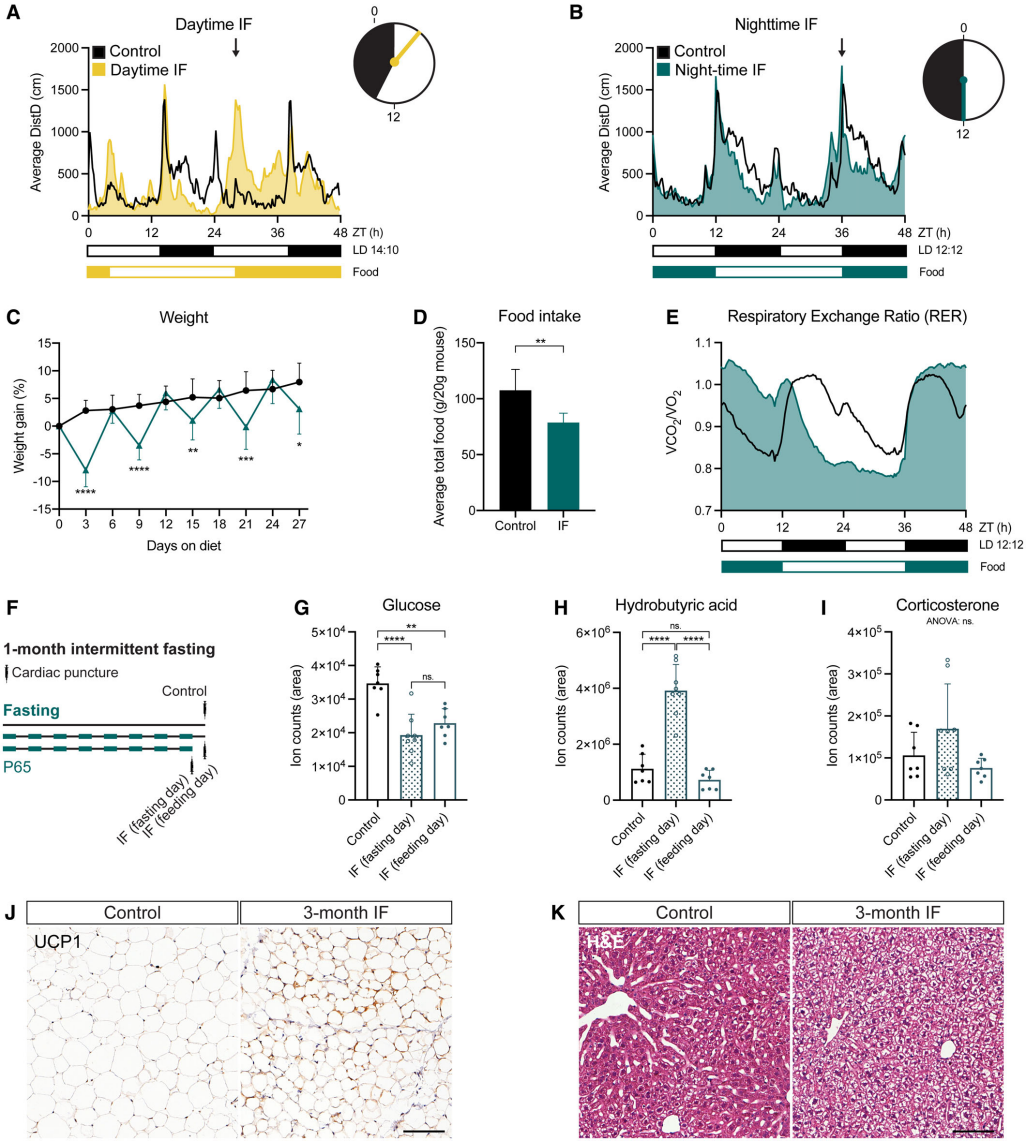
Night‐time IF induces systemic features of IF without disrupting the circadian activity pattern of mice A, B
Mouse locomotor activity in control (*ad libitum*, *n*_control‐daytime_ = 3, *n*_control‐night‐time=_6), daytime IF (A, *n* = 4), and night‐time IF (B, *n* = 6) mice. Activity is displayed as average distance covered by mice in 48‐h cycles throughout a 1‐month long intervention. Black and white boxes under the graphs indicate respectively the dark and light phases of the light:dark (LD) cycle with zeitgeber time (ZT) 0 being the time of lights ON. Yellow/blue and white boxes indicate the presence and absence of food respectively. Arrows indicate the time of food availability onset. The clocks represent 24‐h cycles in ZT with light and dark phases filled in white and black respectively. Clock hands indicate time of food change: ZT3 for daytime IF and ZT12 for night‐time IF. Daytime IF introduces abnormal peaks of activity during the day disrupting the circadian rhythmicity of mouse activity while night‐time IF preserves their normal activity pattern.C
Mouse weight every 3 days shown as the percentage of weight difference to the first day of the diet. Days 3, 9, 15, 21 and 27 show weight on fasting days, and days 6, 12, 18 and 24 on feeding days. Weight oscillates in feeding and fasting days. *n*_control_ = 17, *n*_IF_ = 18. Two‐way ANOVA *P*_time_ < 0.0001, *P*_diet_ = 0.0005, *P*_int_ < 0.0001; followed by Šídák's multiple comparisons test; *P* of days 3–27 in order: < 0.0001, > 0.9999, < 0.0001, 0.6993, 0.0100, 0.8441, 0.0001, 0.8960, 0.0119.D
Average total food consumed for a month shown as g of food per 20 g of mouse weight (weight reference of mice at the beginning of the diet). IF induces a mild (~ 70%) caloric restriction. *n*_Control_ = 17, *n*_IF_ = 18. Two‐tailed unpaired *t*‐test, *P* = 0.0013.E
Respiratory Exchange Ratio (RER) as a comparison of CO_2_ over O_2_ volumes. RER values close to 1 indicate predominant carbohydrate metabolism as energy fuel, while RER values close to 0.7 indicate a shift towards lipid oxidation. RER is displayed as average of 48 h cycles throughout a 1‐month long intervention. IF induces a longer shift towards lipid oxidation. *n* = 6 in both groups.F
The serum of mice was obtained by cardiac puncture after 1 month of *ad libitum* eating (control) or IF. Two independent IF groups were used to collect serum on a fasting or feeding day.G–I
Targeted metabolomics were performed to determine the levels of glucose (G), hydrobutyric acid (H), and corticosterone (I) in the serum of mice. The area of ion counts is displayed. *n*_control_ = 7, *n*_IF(fasting day)_ = 8, *n*_IF(feeding day)_ = 7. One‐way ANOVA, (G) *P* < 0.0001, (H) *P* < 0.0001, (I) *P* = 0.0613; followed by Tukey's multiple comparisons test, (H) *P*_control‐IFfasting_ < 0.0001, *P*_control‐IFfeeding_ = 0.0014, *P*_IFfasting‐IFfeeding_ = 0.4061, (G) *P*_control‐IFfasting_ < 0.0001, *P*_control‐IFfeeding_ = 0.5180, *P*_IFfasting‐IFfeeding_ < 0.0001.J
Representative images of UCP1‐stained inguinal adipose tissue after 3 months of IF. Adipocytes of IF mice look smaller and show a mild increase in UCP1. Images of all animals can be found in Appendix Fig S1A.K
Representative images of liver tissue stained with H&E after 3 months of IF. IF induces hepatocyte swelling. Images of all animals can be found in Appendix Fig S1B. Data information: Bars and error bars represent average + s.d.; dots represent individual mice except in (C), where they represent the average. Significance summary: ns (or absence of sign in C), *P* > 0.05; *, *P* < 0.05; **, *P* < 0.01; ***, *P* < 0.001; ****, *P* < 0.0001. Scale bars: 100 μm. Source data are available online for this figure.

**Figure EV1:**
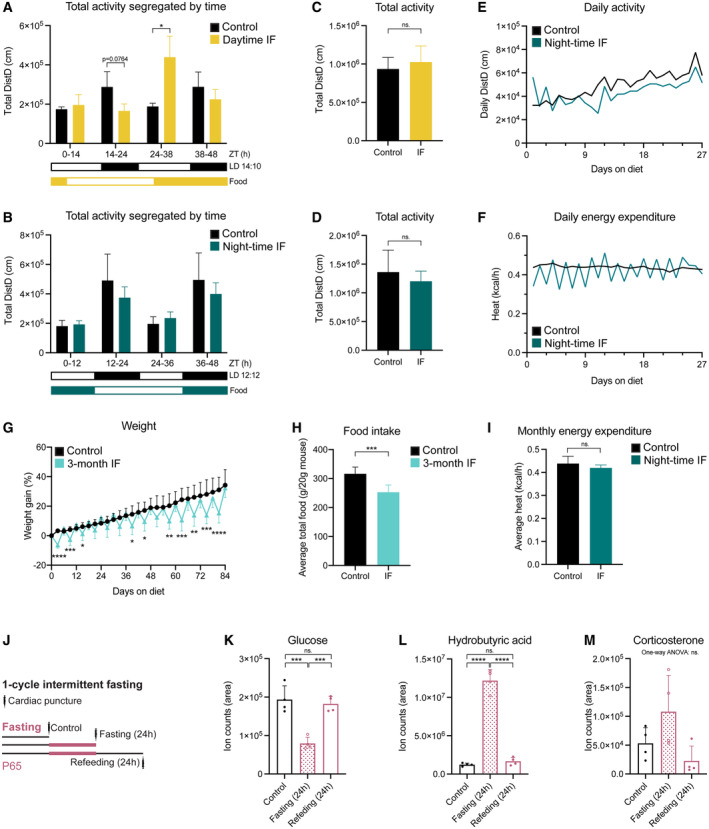
Systemic effects of IF A, B
Mouse locomotor activity in control (*ad libitum*, *n*_control‐daytime_ = 3, *n*_control‐night‐time_ = 6), daytime IF (A, *n* = 4), and night‐time IF (B, *n* = 6) mice displayed as sum of activity during 1 month of IF segregated by time of the day (grouped according to light phase). Black and white boxes under the graphs indicate respectively the dark and light phases of the light:dark (LD) cycle with zeitgeber time (ZT) 0 being the time of lights ON. Yellow/blue and white boxes indicate the presence and absence of food respectively. Expected differences between light and dark phases were observed. Daytime IF increased locomotor activity during the feeding day while night‐time IF preserved the activity pattern. Two‐way ANOVA followed by Šídák's multiple comparisons test. See significance values in Appendix Table S3.C, D
Total mouse locomotor activity during 1 month of daytime (C) or night‐time (D) IF. Two‐tailed unpaired *t*‐test; (C) *n*_control‐daytime_ = 3, *n*_IF‐daytime_ = 4, *P* = 0.5596, (D) *n*_control‐night‐time_ = 6, *n*_IF‐night‐time_ = 6, *P* = 0.3698.E, F
Daily mouse activity (E) and energy expenditure (F) over time in control and night‐time IF mice. Mice in the two conditions follow comparable trends except for daily activity during the first 3–4 days, when activity is more variable in night‐time IF mice than in control mice.G
Mouse weight shown as the percentage of weight difference to the first day of the diet. Weight oscillations persist throughout the 3 months of IF and there is no weight loss on refeeding days compared to control mice. *n*_control_ = 17, *n*_IF_ = 17. Mixed‐effects analysis *P*_time_ < 0.0001, *P*_diet_ = 0.0215, *P*_int_ < 0.0001; followed by Šídák's multiple comparisons test, see significance values in Appendix Table S4.H
Average total food consumed for 3 months shown as g of food per 20 g of mouse weight (weight reference of mice at the start of the diet, P65). *n*_control_ = 17, *n*_IF_ = 17. Two‐tailed unpaired *t*‐test, *P* = 0.8333.I
Monthly energy expenditure displayed as average heat per hour. *n*_control_ = 6, *n*_IF_ = 6. Two‐tailed unpaired *t*‐test, *P* = 0.2141.J
The serum of mice was obtained by cardiac puncture after 1 month of *ad libitum* eating (control) or IF. Different groups were used to collect serum after a fasting or feeding day.K–M
Targeted metabolomics were performed to determine the levels of glucose (K), hydrobutyric acid (L) and corticosterone (M) in the serum of mice. The area of ion counts is displayed. *n*_control_ = 4, *n*_IF(fasting day)_ = 4, *n*_IF(feeding day)_ = 4. One‐way ANOVA, (K) *P* = 0.0002, (L) *P* < 0.0001, (M) *P* = 0.0515; followed by Tukey's multiple comparisons test when the ANOVA was significant, (K) *P*_control‐IFfasting_ = 0.0004, *P*_control‐IFfeeding_ = 0.8032, *P*_IFfasting‐IFfeeding_ = 0.0008; (L) *P*_control‐IFfasting_ < 0.0001, *P*_control‐IFfeeding_ = 0.7749, *P*_IFfasting‐IFfeeding_ < 0.0001. Data information: Bars and error bars represent average + s.d., dots represent individual mice. Significance summary: ns, *P* > 0.05; *, *P* < 0.05; **, *P* < 0.01; ***, *P* < 0.001; ****, *P* < 0.0001.

We first set out to establish a fasting protocol to study adult neurogenesis. IF is a popular form of fasting that encompasses a group of dietary interventions that rely on alternating fasting and feeding periods (Hofer *et al*, 2022). One of the most widely used IF interventions ‐and the one we chose for this study‐ is every‐other‐day‐fasting (also known as alternate‐day‐fasting), which consists of cycles of 24 h of food deprivation followed by 24 h of free access to food, where food is removed or added always at the same time of the day. The specific time at which food is removed/added respect to the time at which lights are switched on in the animal house (usually referred to as Zeitgeber Time 0 or ZT0) varies among studies, although it is often not specified. Morning (ZT0‐ZT4, daytime IF) and evening (ZT12‐14, night‐time IF) times for removing/adding food are commonly used in IF studies, including those showing effects on adult neurogenesis in young, healthy mice (M. Mattson and S. Thuret, personal communication December 2020 and June 2021 respectively). We monitored mouse behaviour in *ad libitum* fed (Control, free access to food), day‐ and night‐time intermittently fasted mice for 1 month using automated home cage phenotyping PhenoMaster cages (TSE Systems). *Ad libitum* fed mice displayed higher activity levels during the night than during the day, with a prominent peak of activity at the beginning of the dark phase, as expected for nocturnal animals (Figs 1A and B, and EV1A and B). It has been previously shown that IF with daytime refeeding decouples feeding patterns from light cues and daily behaviour, flattening the rhythmic expression of circadian clock genes (Froy *et al*, 2009; Acosta‐Rodríguez *et al*, 2022). Accordingly, daytime refeeding introduced an additional peak of activity during the light phase and decreased the general activity of mice during the night (Figs 1A and EV1A). Night‐time IF, on the other hand, synchronised food cues to the light/dark cycle preserving the normal circadian activity pattern of mice (Figs 1B and EV1B). Interestingly, the total activity after 1 month of diet was not affected by day‐ nor night‐time IF compared to control mice (Fig EV1C and D). The activity over time of the mice as well as their daily energy expenditure also followed the same trend in control and night‐time groups (Fig EV1E and F). Since adult neurogenesis is influenced by circadian rhythms (Holmes *et al*, 2004; Bouchard‐Cannon *et al*, 2013; Draijer *et al*, 2019), we chose the night‐time IF protocol to study the effects of IF without introducing circadian rhythm disruption as an additional variable.

### Night‐time IF induces characteristic features of IF in mice

We then characterised the systemic response of mice to night‐time IF (from here on IF unless stated otherwise) during 1 and 3 months. Every 24 h of fasting resulted in a weight loss of up to 7% of the original weight, which was recovered during the following 24 h of free access to food (Figs 1C and EV1G). These oscillations persisted throughout the whole 3‐month intervention. We also observed that mice subjected to IF ate nearly twice as much as control mice on feeding days. Quantification of overall food consumption by the end of the treatment showed a caloric restriction of 73 or 79.8% upon 1 or 3 months of IF respectively (Figs 1D and EV1H). Despite the observed caloric restriction, the monthly energy expenditure was comparable between groups and fasted mice exhibited a similar growth curve to *ad libitum* fed mice and a comparable weight at the end of the IF protocol even after 3 months of diet (Figs 1C and EV1G–I).

To characterise the impact of IF on systemic metabolism we performed an indirect calorimetry during a whole month of IF to estimate the fuel source used by the body to produce energy. We used the automated home cage phenotyping system to monitor CO_2_ production and O_2_ consumption, with which we calculated the respiratory exchange ratio (RER). A RER value close to 0.7 indicates predominant usage of lipid oxidation, characteristic of resting periods, while a value around 1 indicates that carbohydrates are the main source of energy. As expected, the RER of control mice was higher during the night than during the day (Fig 1E). In IF mice, fasting extended the periods in which the body shifted its metabolism towards lipid oxidation, a feature of prolonged fasting, while feeding induced a sharp metabolic shift towards the use of carbohydrates as a fuel source (Fig 1E). Therefore, alternating fasting and feeding days resulted in the periodic metabolic shifts that are proposed to mediate the benefits of IF (Anton *et al*, 2018).

To further confirm that IF induced the expected effects in our protocol, we measured the levels of fasting‐related metabolites in the serum of control and fasted mice both after the first cycle of fasting and after 1 month of IF (Figs 1F and EV1J). Glucose levels were reduced in fasted mice respect to control mice on fasting days, both after 24 h and 1 month of fasting (Figs 1G and EV1K). On the next refeeding day, glucose levels rapidly recovered in mice fasted for 24 h but stayed low in mice subjected to IF for 1 month, indicating long‐lasting changes in glucose metabolism and improved insulin sensitivity in IF mice, as previously described. On fasting days, either after one cycle or 1 month, we also observed a marked increase in the levels of hydrobutyric acid in the serum of fasted mice, a consequence of the use ketone bodies as a fuel source normally associated with low glucose levels (Figs 1H and EV1L). We also measured corticosterone as a readout of stress levels. There were no significant differences between control and fasted mice, although we did observe a trend towards higher levels of corticosterone on fasting days at both time points (Figs 1I and EV1M).

Browning of adipose tissue is another indicator of fasting (Li *et al*, 2017; Liu *et al*, 2019). Fat browning is characterised by a reduction in adipocyte size and an upregulation of uncoupling protein 1 (UCP1), which regulates mitochondrial energy production, associated with thermogenesis in these cells (Ricquier, 2011). After 3 months of IF, inguinal adipocytes of IF mice looked smaller and had higher levels of UCP1 than those of mice fed *ad libitum*, although the effects were mild and variable (Fig 1J and Appendix Fig S1A). We also observed robust structural changes in the livers of IF mice (Fig 1K and Appendix Fig S1B), where hepatocytes showed an oedematous morphology. This hepatocyte oedema resembled the one reported upon 1‐week of IF as a compensatory mechanism to fluctuating liver size between the fasting and fed states (preprint: Sarkar *et al*, 2021). Our data show that liver remodelling persists after 3 months of IF. Together, these results demonstrate that cycles of 24 h fasting and 24 h feeding induce typical features of IF.

### IF does not affect the proliferation or maintenance of adult NSCs in the hippocampus

**Figure 2:**
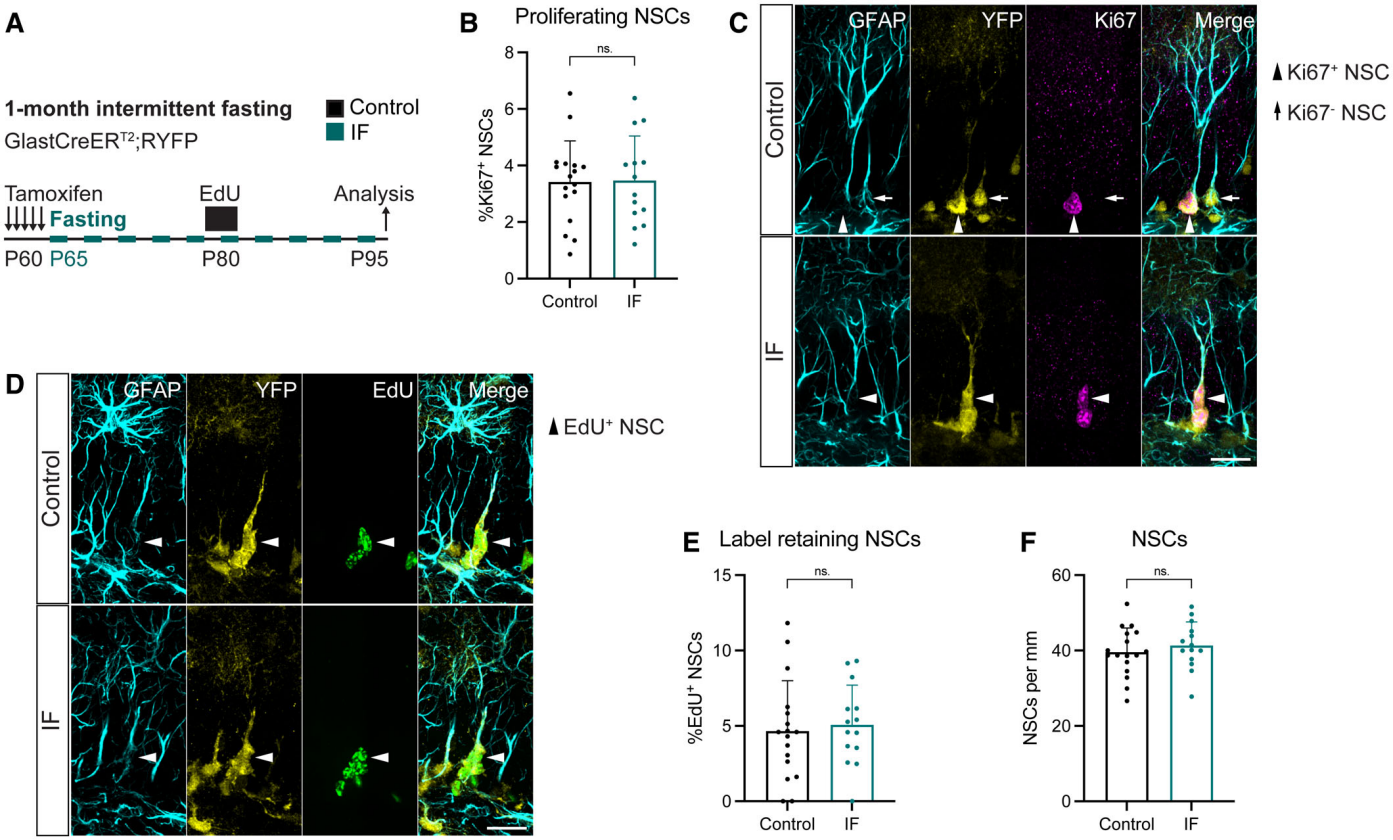
1 month of IF does not affect NSC proliferation, quiescence/activation transitions nor maintenance A
2‐month‐old GlastCreER^T2^;RYFP mice were administered tamoxifen on 5 consecutive days to fluorescently (YFP) label NSCs, after which, they were subjected to every‐other‐day IF for 1 month. EdU was administered in the drinking water for 5 days, 15 days before the analysis.B, C
Images of proliferating NSCs in control and IF mice, and quantification of the percentage of proliferating NSCs. NSCs were identified by their localisation in the SGZ, the presence of a single GFAP^+^ vertical projection and the help of YFP. Nuclear colocalisation with Ki67 was used to distinguish proliferating (Ki67^+^, arrowheads) from quiescent (Ki67^−^, arrow) NSCs. The Ki67^−^ NSC is only shown for comparison, as it is unlikely to be the daughter cell of the proliferating NSC in the same picture. The percentage of proliferating NSCs was unchanged by IF. Two‐tailed unpaired *t*‐test, *P* = 0.9165.D, E
Images of EdU retaining NSCs in control and IF mice and quantification of their percentage. Arrowheads indicate EdU^+^ NSCs. IF did not affect the percentage of label retaining NSCs. Two‐tailed unpaired *t*‐test, *P* = 0.7046.F
Total number of NSCs normalised to DG length per 40‐μm‐thick section. The number of NSCs is unaffected by IF. Two‐tailed unpaired *t*‐test, *P* = 0.4452. Data information: Bars and error bars represent average + s.d.; dots represent individual mice; *n*_control_ = 17, *n*_IF_ = 14. Significance summary: ns, *P* > 0.05. Scale bars: 20 μm. Source data are available online for this figure.

**Figure EV2:**
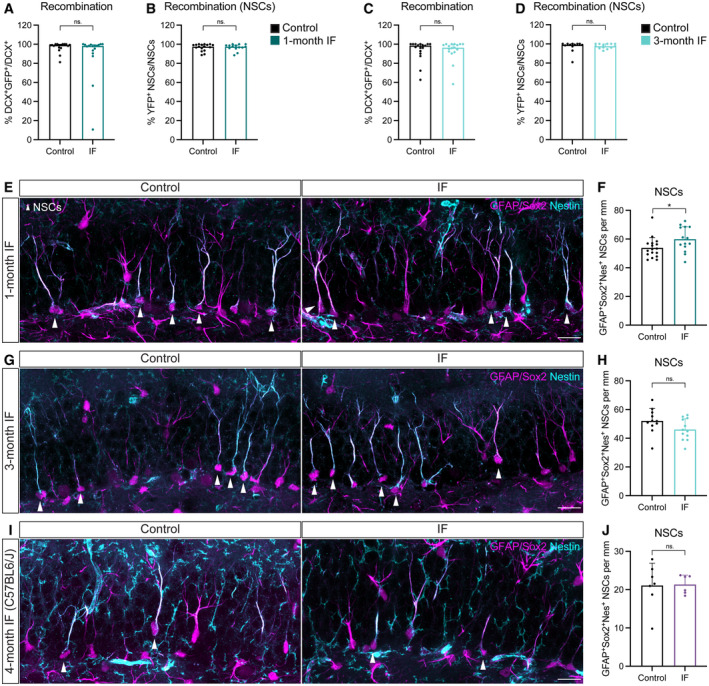
High recombination in Glast‐CreER^T2^;RYFP throughout conditions, and additional NSC analysis A–D
Recombination of the YFP reporter in Glast‐CreER^T2^;RYFP upon tamoxifen induction shown as YFP^+^ neuroblasts (A, C) or NSCs (B, D) over their total numbers after 1 (A, B) or 3 (C, D) months of treatment. Only animals with a recombination rate higher than 80% were considered for further analysis. Mann–Whitney tests. (A) *n*_control_ = 17, *n*_IF_ = 17, *P* = 0.3233; (B) *n*_control_ = 17, *n*_IF_ = 14, *P* = 0.7760; (C) *n*_control_ = 17, *n*_IF_ = 17, *P* = 0.7818; (D) *n*_control_ = 12, *n*_IF_ = 13, *P* = 0.5318. Bars and error bars represent median + interquartile range.E–J
Images of NSCs (arrowheads) and quantifications normalised to DG length per 40‐μm section in Glast‐CreER^T2^;RYFP mice after 1 (E, F) or 3 (G, H) months of *ad libitum* (control) eating or IF, and C57BL6/J mice after 4 months of diet (I, J). NSCs were identified as cells with a Sox2^+^ nucleus in the SGZ extending a single GFAP^+^Nestin^+^ radial projection to the molecular layer. (F) *n*_control_ = 17, *n*_IF_ = 14; (H) *n*_control_ = 11, *n*_IF_ = 12; (I) *n*_control_ = 7, *n*_IF_ = 6. Two‐tailed unpaired *t*‐tests; (F) *P* = 0.0414, (H) *P* = 0.09, (I) *P* = 0.9243. Bars and error bars represent average + s.d. Data information: Control (*ad libitum*) for each experiment (black), 1‐month IF (dark blue), 3‐month IF (light blue), 4‐months IF C57BL6/J (purple). Dots represent individual mice. Significance summary: ns, *P* > 0.05; *, *P* < 0.05.

To specifically characterise how adult NSCs respond to IF, we used Glast‐CreER^T2^;RYFP mice, a well‐established mouse transgenic model for lineage tracing of NSCs in the adult DG in which Tamoxifen‐induced Cre recombination indelibly labels Glast‐expressing cells with YFP (Srinivas *et al*, 2001; Mori *et al*, 2006; Andersen *et al*, 2014). Tamoxifen was given to 2‐month‐old mice for the 5 days prior to starting the diet and we examined the mice on a feeding day 1 month after the diet began (Fig 2A). The rate of recombination was very high throughout our experiments and identical in *ad libitum*‐fed and intermittently fasted mice (Fig EV2A–D). We identified NSCs by their localisation in the sub‐granular zone (SGZ) and the extension of a single glial fibrillary acidic protein (GFAP)^+^ radial projection to the molecular layer with help of the YFP fluorescent reporter. A small fraction of NSCs (~ 3.4%) was positive for the proliferation marker Ki67 in control mice, as expected for a predominantly quiescent NSC population. This number was unchanged by IF, suggesting that NSC proliferation is not affected by this intervention (Fig 2B and C).

NSCs can return to quiescence after proliferating, slowing down the activation‐triggered loss of the stem cell pool. We used the thymidine analogue 5‐ethynyl‐2′‐deoxyuridine (EdU) in a label retention experiment to explore potential changes in the ability of adult NSCs to return to quiescence (Urbán *et al*, 2016). We administered EdU in the drinking water for 5 days to label all cells going through the S‐phase of the cell cycle during that period. After a 10‐day chase period, we quantified NSCs that had incorporated EdU during the 5‐day labelling period and had retained both the label and their NSC features (i.e. SGZ localisation and a single GFAP^+^ radial process) (Fig 2A). The percentage of EdU‐retaining NSCs was similar in control and IF mice (Fig 2D and E). This, together with the similar NSC proliferation rates in control and IF mice, suggests that NSC transitions between quiescence and activation are not altered by IF. The total number of NSCs was also not changed between IF and *ad libitum* fed mice (Fig 2F). Together, our results show that adult NSCs are not affected by 1 month of IF.

**Figure 3:**
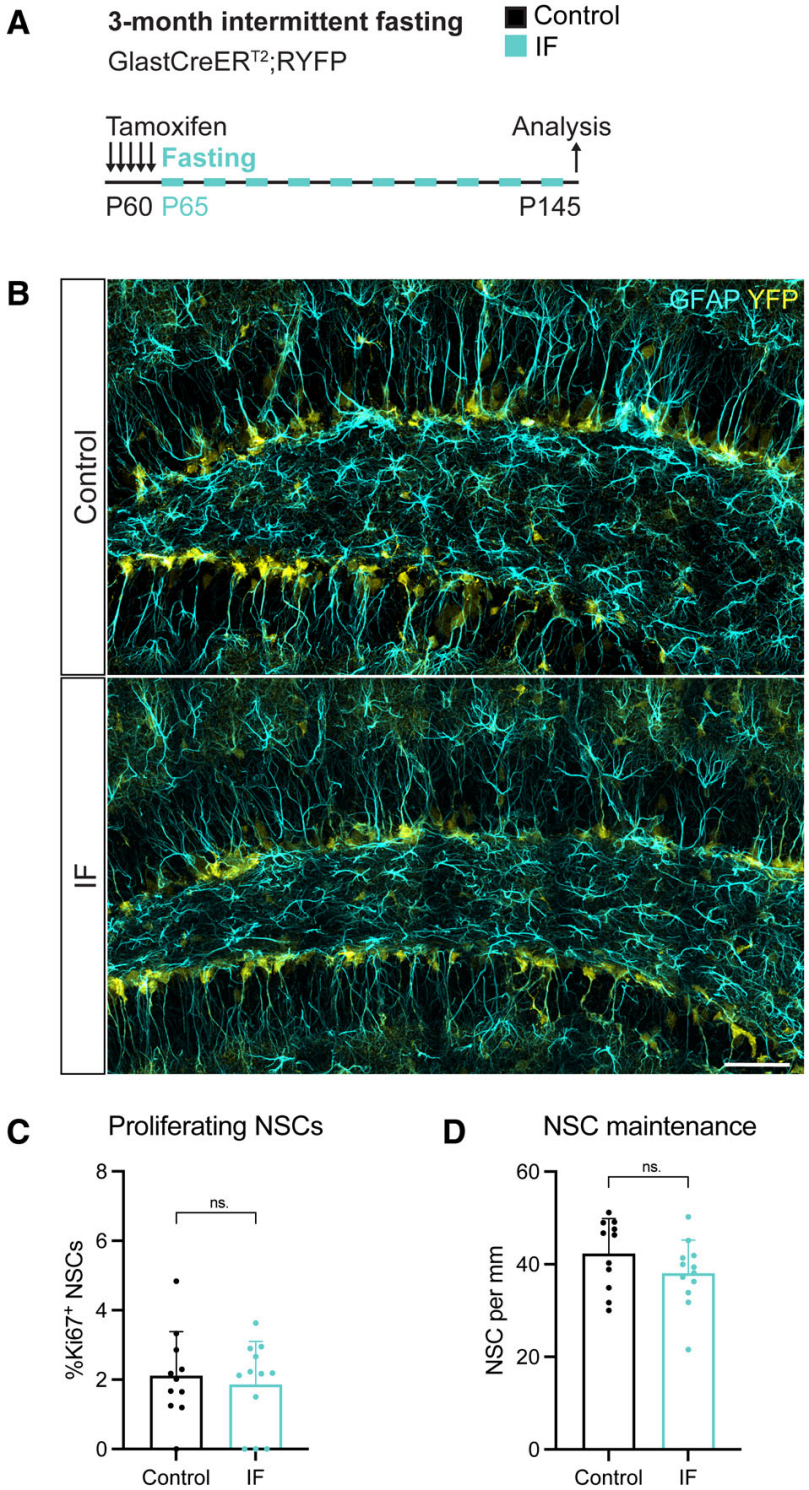
3 months of IF do not affect NSC proliferation nor maintenance A
2‐month‐old GlastCreERT2;RYFP mice were administered tamoxifen on 5 consecutive days to fluorescently (YFP) label NSCs, after which they were subjected to every‐other‐day IF for 3 months.B
Images of NSCs identified by their localisation in the SGZ, the presence of a single GFAP^+^ vertical projection, and the help of YFP. Arrowheads show several NSCs.C, D
Quantification of the percentage of proliferating NSCs and the total number of NSCs in control and IF mice normalised to DG length per 40‐μm‐thick section. IF mice show similar levels of proliferating and total NSCs to control mice. Two‐tailed unpaired *t*‐tests; (C) *P* = 0.6317, (D) *P* = 0.1779. Data information: Bars and error bars represent average + s.d.; dots represent individual mice; *n*_control_ = 11, *n*_IF_ = 12. Significance summary: ns, *P* > 0.05. Scale bar: 50 μm. Source data are available online for this figure.

To understand the consequences of longer exposures to IF, we subjected Glast‐CreER^T2^;RYFP mice to 3 months of IF (Fig 3A), which has been shown to be enough to increase neuronal production in the DG (Lee *et al*, 2002a,b; Kitamura *et al*, 2006; Kim *et al*, 2015; Baik *et al*, 2020; Li *et al*, 2020; Dias *et al*, 2021). The percentage of proliferating NSCs in the 3‐month intervention was halved compared to the same value in the 1‐month intervention, in accordance with the reported increase in quiescence during ageing (Harris *et al*, 2021) (Figs 2B and 3C). NSC proliferation did not change upon IF (Fig 3C). We then examined NSC maintenance by quantifying the total number of NSCs present at the end of the treatment (Fig 3B and D). Despite the well‐established decrease of neurogenesis with age, we did not observe a decrease in total NSCs in 5‐month‐old mice respect to 3‐month‐old mice (Figs 2F and 3D). This could be explained by batch variability in the stainings or by a shift in the exponential phase of the decline in NSC numbers to either before 3 months or after 5 months of age in these mice. Nevertheless, the number of NSCs in IF mice were indistinguishable from those of *ad libitum* fed mice (Fig 3B and D).

As an alternative to using YFP to identify NSCs in Glast‐CreER^T2^;RYFP mice, which could be biased towards a specific high Glast‐expressing population, we also quantified NSCs based on their expression of Nestin, GFAP and Sox2, their location and morphology. We observed a small increase in the number of NSCs after 1 month of IF, with NSC numbers going back to control levels after 3 months of IF (Fig EV2E–H), again indicating that the long‐term maintenance of NSCs is not affected by IF. Similar to our previous quantification, we did not observe a marked decline in NSC numbers between 3‐ and 5‐month‐old mice, suggesting that indeed the exponential decline in NSCs does not happen during this time‐frame in Glast‐CreER^T2^;RYFP mice.

Overall, these data indicate that NSCs are protected from proliferation bursts that could lead to exhaustion, making IF a safe option for potentially increasing neurogenesis through diet‐based interventions.

### Adult neurogenesis is mildly and transiently impaired by IF

**Figure 4:**
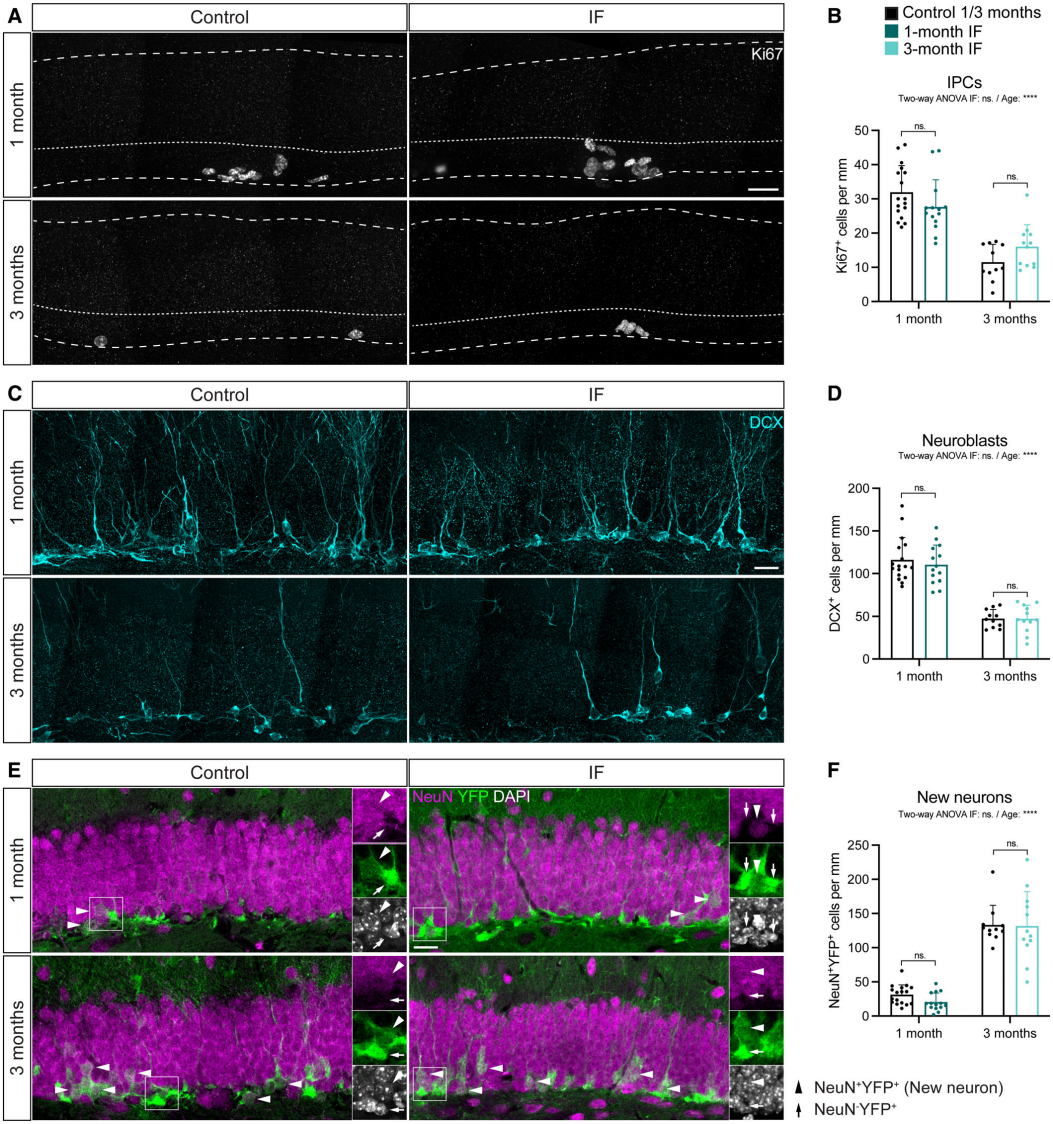
IF does not affect IPCs nor neuroblasts and only mildly and temporarily decreases the neuronal output A, B
Images of Ki67^+^ cells in control and IF mice after a 1‐ or 3‐month intervention as a proxy for IPCs and quantification of IPCs normalised to DG length per 40‐μm‐thick section. Dashed and dotted lines indicate DG area and border between the SGZ and the molecular layer respectively. IPCs were unchanged by IF. Two‐way ANOVA; *P*_IF_ = 0.948, *P*_Age_ = < 0.0001.C, D
Images of neuroblasts identified by the marker for immature neurons DCX and quantification normalised to DG length per 40‐μm‐thick section. IF did not affect the number of neuroblasts. Two‐way ANOVA; *P*_IF_ = 0. 6,092, *P*_Age_ < 0.0001.E, F
Images of newly born neurons (arrowheads) identified by the colocalisation of the mature neuronal marker NeuN and the YFP reporter, indicating that these neurons were generated from the population of NSCs labelled with YFP at the beginning of the diet. Quantification of newly born neurons normalised to DG length per 40‐μm‐thick section. The number of new neurons transiently decreased upon 1 month of IF but was unchanged after 3 months. Two‐way ANOVA; *P*_IF_ = 0.2642, *P*_Age_ < 0.0001. Data information: Bars and error bars represent average + s.d.; dots represent individual mice. *n*_control‐1m_ = 17, *n*_IF1m_ = 14, *n*_control‐3m_ = 11, *n*_IF‐3m_ = 12. See Tukey's multiple comparisons test values in Appendix Table S1. Significance summary: ns, *P* > 0.05; ****, *P* < 0.0001. Scale bar: 20 μm. Source data are available online for this figure.

Since neither the pool nor the activity of NSCs change upon IF, we hypothesised that the previously reported increase in neurogenesis laid in subsequent stages of neurogenesis. We set out to determine at which step/s of the neurogenic lineage IF influences adult neurogenesis. NSCs and neuroblasts are predominantly out of the cell cycle, meaning that the vast majority of proliferating cells in the SGZ are IPCs (Kronenberg *et al*, 2003; Hodge *et al*, 2008). We therefore used Ki67 as a proxy for IPC identification. As previously reported, we observed a decrease in IPC abundance over time (Fig 4A and B). IF neither changed the number of IPCs after 1 month nor prevented their decline after 3 months (Fig 4A and B).

Next, we quantified the number of neuroblasts, identified by doublecortin (DCX) immunoreactivity. Our results show that neuroblasts are more abundant in younger mice and that they are generated at the same rate in IF mice and *ad libitum* fed mice at both time points (Fig 4C and D).

**Figure EV3:**
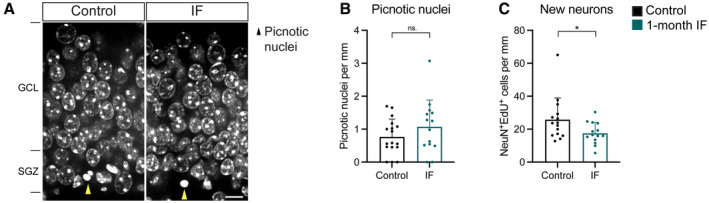
Transient decrease in the generation of new neurons upon 1 month of IF. See experimental design in Fig 2A A
Images of picnotic nuclei (yellow arrowheads) in the SGZ of the DG in control and IF mice after 1 month of diet.B
Quantification of picnotic nuclei normalised to DG length per 40‐μm‐thick section as a proxy for cell death, which was not affected by IF. *n*_control_ = 17, *n*_IF_ = 14. Two‐tailed unpaired *t*‐test, *P* = 0.2172.C
Number of EdU‐labelled neurons (as NeuN^+^ cells) normalised to DG length per 40‐μm‐thick section after a 10‐day chase, showing a decrease in the number of new neurons generated. Note that 10 days is shorter than the conventional 1 month of chase, which is why neuron numbers are still very low. *n*_control_ = 15, *n*_IF_ = 14. Two‐tailed unpaired *t*‐test, *P* = 0.0427. Data information: Bars and error bars represent average + s.d.; dots represent individual mice. Significance summary: ns, *P* > 0.05; *, *P* < 0.05. Scale bar: 10 μm.

Finally, we measured the number of new neurons generated during the intervention. We used colocalisation of the YFP reporter and the marker for mature neurons NeuN to identify newly born neurons. In accordance with the cumulative labelling of neurons upon lineage tracing, mice had many more newly born neurons labelled with the YFP reporter after 3 months than after 1 month (Fig 4E and F). We found a similar number of newly born neurons in IF mice compared to *ad libitum* fed mice after 1 or 3 months of IF (Fig 4E and F). We noticed a slight (non‐significant) reduction in the number of newly born cells after 1 month of IF. This prompted us to measure cell death by quantifying the number of picnotic nuclei in the SGZ, which we found was comparable in control and IF mice (Fig EV3A and B). Since neuroblasts were produced at the same rate (Fig 4D), we wondered whether there could be a transient drop in neurogenesis at an earlier time point. We made use of the label retention experiment in Fig 2A to ask if neuronal production was impaired 2 weeks after starting IF. Indeed, we found that fewer neurons had been produced at that time in mice subjected to IF compared to control mice (Fig EV3C). One month of IF is therefore not only insufficient to increase adult neurogenesis but in fact mildly and transiently decreases the neuronal output of adult NSCs.

Our findings show that 1 or 3 months of IF do not increase adult neurogenesis. This seemingly contradicts previous reports and puts a question mark on the suitability of IF as a tool to increase neurogenesis in the adult hippocampus.

### IF does not increase adult neurogenesis regardless of sex, labelling method, strain, tamoxifen usage or diet length

We next asked which intrinsic variables of our study could explain the contradicting results with previously published studies.

**Figure 5:**
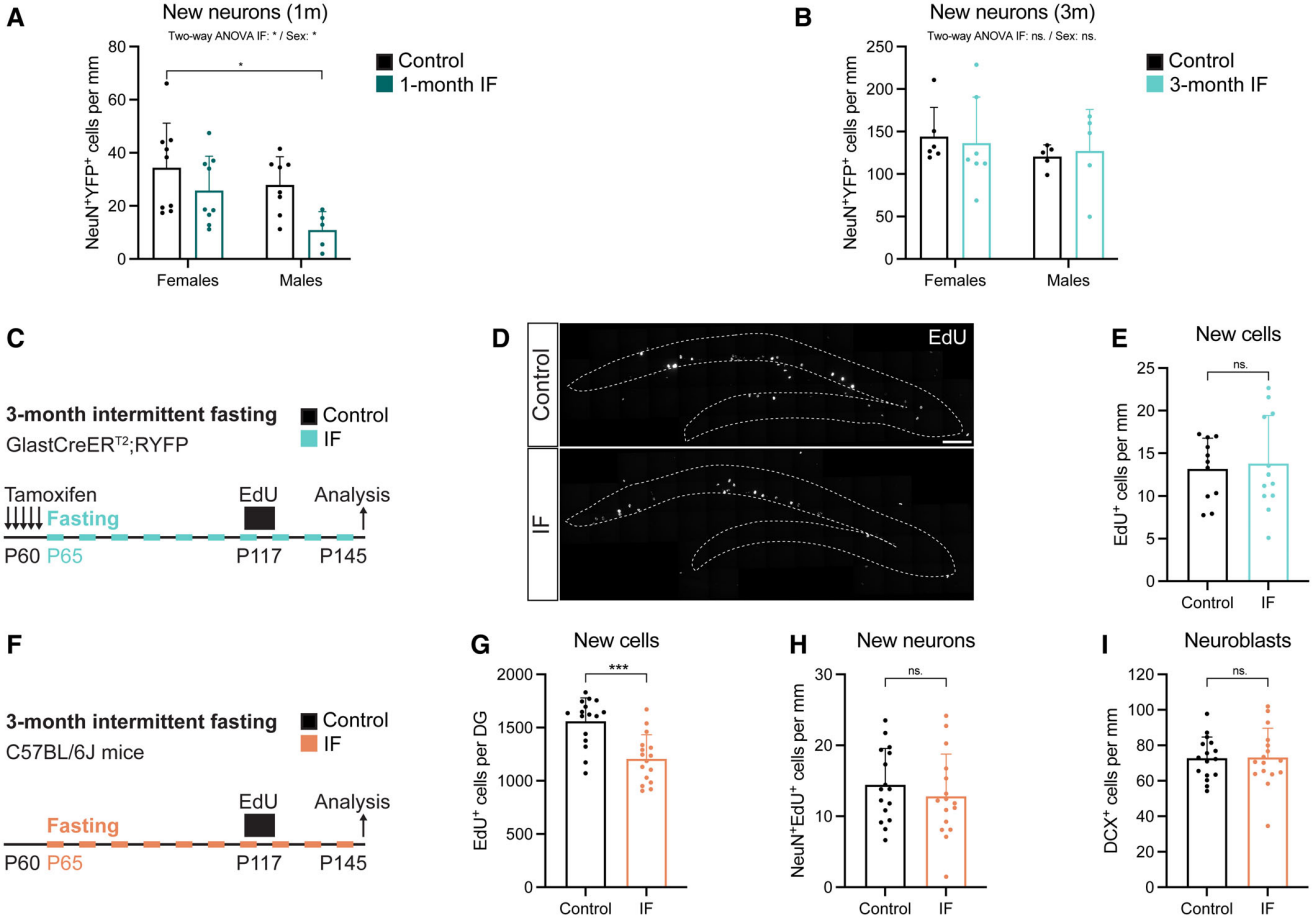
Sex, labelling method, tamoxifen or strain do not alter the neurogenic response to IF A, B
Quantification of newly born neurons after 1 (A) or 3 (B) months of IF from Fig 4F segregated by sex. See all sex‐segregated data in Fig EV4 and images of (B) in Appendix Fig S3. (A) *n*_control♀_ = 9, *n*_control♂_ = 8, *n*_IF♀_ = 9, *n*_IF♂_ = 5; (B) *n*_control♀_ = 6, *n*_control♂_ = 5, *n*_IF♀_ = 7, *n*_IF♂_ = 5. Two‐way ANOVA; (A) *P*_IF_ = 0.0129, *P*_Sex_ < 0.0352; (B) *P*_IF_ = 0.3728, *P*_Sex_ < 0.9784; followed by Tuckey's multiple comparisons tests (see significance values in Appendix Table S2). Sex differences were found only in the number of new neurons at 1 month.C
Survival assay with a thymidine analogue to replicate the labelling method of previous publications. EdU was administered for 4 days to GlastCreER^T2^;RYFP mice (see experimental in Fig 3) that had undergone 2 months of IF to label a cohort of proliferating cells that was analysed after a 1‐month chase, giving the cells time to progress in the neurogenic lineage.D, E
Images of new cells in the DG (enclosed in dashed lines) shown by EdU‐labelled cells 1 month after EdU administration and quantification normalised to DG length per 40‐μm‐thick section. *n*_control_ = 11, *n*_IF_ = 12. Two‐tailed unpaired *t*‐test, *P* = 0.7591. The number of new cells in the DG was unchanged by IF.F
Evaluation of tamoxifen and strain impact on the effects of IF on adult neurogenesis. 2‐month‐old C57BL/6J mice were subjected to 3 months of IF. A survival assay with the thymidine analogue EdU 1 month before the end of the diet was included. *n*_control_ = 16, *n*_IF_ = 16.G
Stereological quantification of new cells in the whole DG. Two‐tailed unpaired *t*‐test, *P* = 0.0001.H
Quantification of newly born neurons normalised to DG length per 40‐μm‐thick section identified by colocalisation of EdU and the marker for mature neurons NeuN. Two‐tailed unpaired *t*‐test, *P* = 0.4144.I
Quantification of new neuroblasts normalised to DG length per 40‐μm‐thick section. Two‐tailed unpaired *t*‐test, *P* = 0.9432. IF did not increase the number of new neurons or neuroblasts in C57BL/6J mice. Data information: Bars and error bars represent average + s.d.; dots represent individual mice. Significance summary: ns, *P* > 0.05; *, *P* < 0.05; ***, *P* < 0.001. Scale bar: 100 μm. Source data are available online for this figure.

**Figure EV4:**
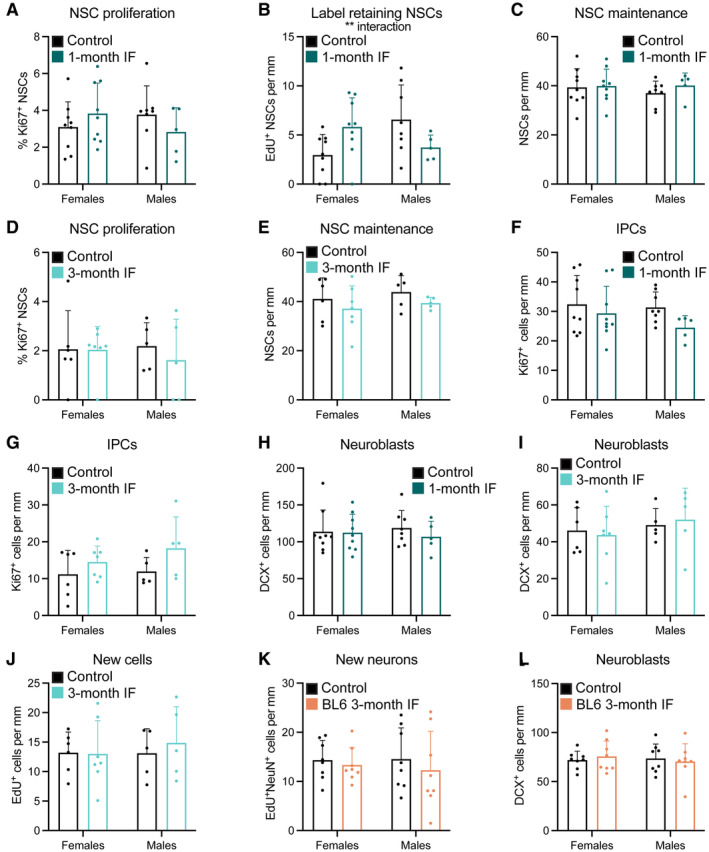
Male and female mice show similar responses to IF A–L
Data of graphs in the main figures segregated by sex showing that the response of adult neurogenesis to IF is sex‐independent. The percentage of label retaining NSCs after 1 month of IF (B) shows an interaction between diet and sex that is not translated in the following steps of the neurogenic lineage. Data information: Graphs are displayed in order of appearance on the text. Bars and error bars represent average + s.d.; dots represent individual mice. (A–C, F, H) *n*_control♀_ = 9, *n*_control♂_ = 8, *n*_IF♀_ = 9, *n*_IF♂_ = 5; (D, E, G, I, J) *n*_control♀_ = 6, *n*_control♂_ = 5, *n*_IF♀_ = 7, *n*_IF♂_ = 5; (K, L) *n* = 8 in all conditions. Two‐way ANOVA (see significance values in Appendix Tables S5 and S6). Significance summary: absence of sign, *P* > 0.05; **, *P* < 0.01.

The metabolism of male and female mice has been shown to react in a sex‐specific manner to fasting and refeeding (Freire *et al*, 2020). While most studies on IF and neurogenesis were conducted with only male or only female mice, differences in the response to caloric restriction of adult hippocampal neurogenesis have been reported between sexes (Park *et al*, 2013). In our experimental set up, we used enough males and females to identify sex differences in the response of adult neurogenesis to IF. We found that the transient decrease in newly born neurons after 1 month of IF was affected by sex, being more prominent in male mice (Fig 5A). However, we did not observe sex differences at any other point of the study, with neurogenesis not being affected by IF in either male or female mice (Figs 5B and EV4 and Appendix Fig S3). Of note, data variability was also similar in male and female mice (Fig EV4).

In this study, we used genetic lineage tracing to quantify the total number of new neurons generated from NSCs throughout the whole dietary intervention. Instead, previous studies relied on the use of thymidine analogues (BrdU) to label a cohort of proliferating cells and quantify the amount of total BrdU^+^ cells or BrdU^+^ neurons 1 month after labelling, obtaining information exclusively on one neurogenic wave at the end of the treatment (Lee *et al*, 2002a,b; Kitamura *et al*, 2006; Brandhorst *et al*, 2015; Kim *et al*, 2015; Dias *et al*, 2021). To assess the effects of IF in a comparable way, we used EdU to pulse‐label proliferating cells 1 month before the end of the 3‐month IF protocol (Fig 5C). Again, the number of EdU^+^ cells (Fig 5D and E) was unchanged by the diet, showing that IF does not affect neurogenesis after 3 months.

**Figure EV5:**
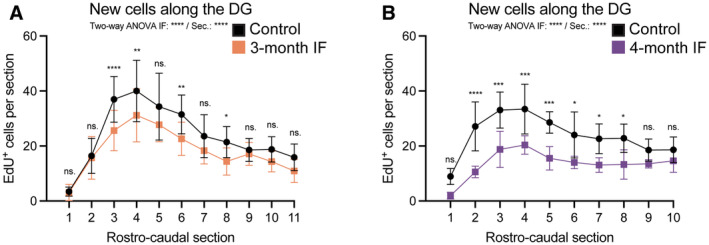
Response to IF along the rostro‐caudal axis of the DG A, B
Number of EdU+ cells along the rostro‐caudal axis of the DG in C57BL6/J mice after 3 or 4 months of IF. See Figs 5 and 6 for experimental design. The average of the two hemispheres in each section was used to calculate the number of cells in a 40‐μm‐thick DG section. The distance between sections is 240 μm. (A) *n*_control_ = 16, *n*_IF_ = 16; (B) *n*_control_ = 7, *n*_IF_ = 6. Two‐way ANOVA followed by Šídák's multiple comparisons test (see significance values in Appendix Table S7). Data information: Dots and error bars represent average ± s.d. Significance summary: ns, *P* > 0.05; *, *P* < 0.05; **, *P* < 0.01; ***, *P* < 0.001, ****, *P* < 0.0001. Sec. = rostro‐caudal section.

Another particularity of our study is that the GlastCreER^T2^;RYFP mice we used had a mixed genetic background (Austin *et al*, 2021), while previous studies had used mice from the C57BL/6J strain. Strain‐specific differences in basal neurogenesis levels as well as the response of adult neurogenesis to dietary interventions such as high‐fat diet have been reported (Hwang *et al*, 2008). Indeed, we found C57BL/6J mice have a higher neurogenic rate than GlastCreER^T2^;RYFP mice at 5 months of age (Appendix Fig S2). In addition, recurrent doses of Tamoxifen alter neurogenesis and induce broad detrimental effects, from damaging the gut epithelia to impairing microglial reactivity (Smith *et al*, 2022). To test whether strain and tamoxifen could alter how IF modulates adult neurogenesis, we subjected C57BL/6J mice to 3 months of IF, excluding tamoxifen administration (Fig 5F). We did a 1‐month EdU‐chase, as in our previous experiment with Glast‐CreER^T2^;RYFP mice, and analysed the number of EdU^+^ cells at the end of the treatment. We found a reduction in EdU^+^ cells in IF compared to control mice (Figs 5G and EV5A). However, when we quantified newly born neurons as EdU^+^NeuN^+^ cells (Fig 5H), we did not find any differences caused by the diet. In addition to that, neuroblasts (Fig 5I) were not altered by IF either suggesting that tamoxifen and strain do not explain the discrepancy in results.

**Figure 6:**
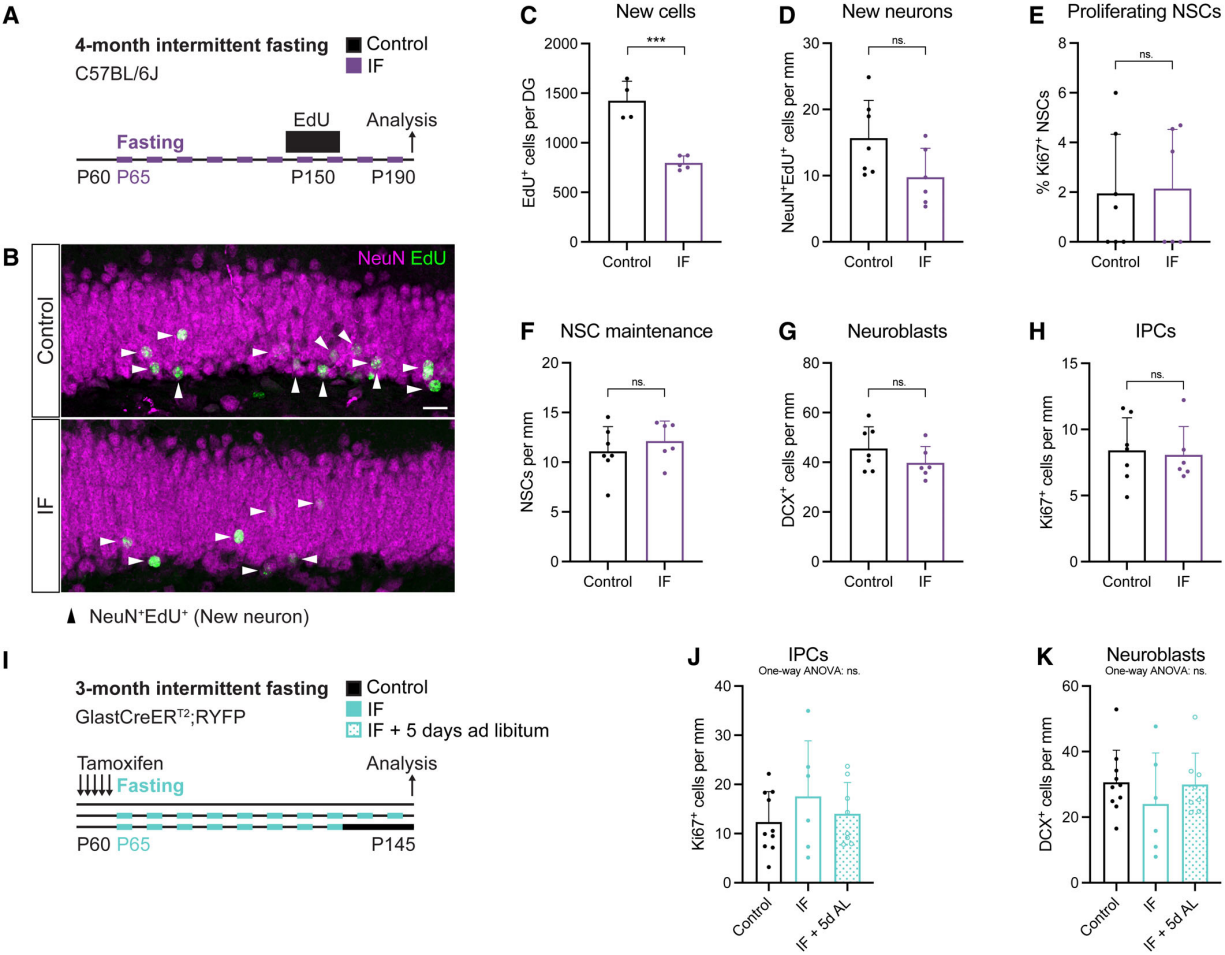
4 months of every‐other‐day IF do not increase adult neurogenesis A
2‐month‐old male C57BL/6J mice were subjected to 4 months of IF, as in previous studies that reported increases in adult neurogenesis. A survival assay with the thymidine analogue EdU (12‐day labelling—1‐month chase) was used to evaluate neurogenesis.B
Image of newly born neurons identified by colocalisation of EdU and the marker for mature neurons NeuN. Arrowheads indicate EdU^+^NeuN^+^ cells in this Z‐plane. Some nuclei appear faint because most of their nucleus was located in adjacent Z‐planes. The whole Z‐stack was used for quantification.C
Stereological quantification of new cells (EdU^+^ cells) in the whole DG of control and 4‐month IF C57BL6/J mice. 4 months of IF induce a decrease in EdU‐labelled neurons. *n*_control_ = 4, *n*_IF_ = 5. Two‐tailed unpaired *t*‐test, *P* = 0.0003.D
Quantification of new neurons (NeuN^+^EdU^+^) normalised to DG length per 40‐μm‐thick section. *n*_control_ = 7, *n*_IF_ = 6. Two‐tailed unpaired *t*‐test, *P* = 0.0628.E–H
Quantification of the percentage of proliferating NSCs (E), and the number of NSCs (F), neuroblasts (G) and IPCs (H) normalised to DG length per 40‐μm‐thick section. The neurogenic lineage is not affected by 4 months of IF. *n*_control_ = 7, *n*_IF_ = 6. Two‐tailed unpaired *t*‐tests; (E) *P* = 0.8863, (F) *P* = 0.4324, (G) *P* = 0.2154, (H) *P* = 0.7970.I
2‐month‐old GlastCreER^T2^;RYFP mice were subjected to 3 months of IF, after which a subset of IF mice had *ad libitum* access to food for 5 days.J, K
Quantification of IPCs (J) and neuroblasts (K) normalised to DG length per 40‐μm‐thick section in control, IF and IF mice with 5 days of *ad libitum* eating before analysis. One‐way ANOVAs; *n*_control_ = 10, *n*_IF_ = 6, *n*_IF+5dAL_ = 8; (J) *P* = 0.4440, (K) *P* = 0.5120. Data information: Bars and error bars represent average + s.d.; dots represent individual mice. Significance summary: ns, *P* > 0.05; ***, *P* < 0.001. Scale bar: 20 μm.

Lee *et al*, Kim *et al* and Dias *et al* reported an increase in neurogenesis in a cohort of cells that was labelled at 3 months of IF and analysed 1 month later, 4 months after the start of the diet (Lee *et al*, 2002a; Kim *et al*, 2015; Dias *et al*, 2021). Since we were interested in determining whether NSCs were at the origin of this increase, we performed 3‐month‐long experiments, meaning that neuronal production was also analysed after 3 months of IF. Therefore, we next asked whether extending the diet for an additional month would increase neurogenesis. We used male C57BL/6J mice and labelled a cohort of proliferating cells with EdU for 12 days, 3 months after the start of the diet (Fig 6A). We analysed newly born cells and neurons 1 month after, and observed a significant decrease in EdU^+^ but not EdU^+^NeuN^+^ cells (Figs 6B–D and EV5B). In addition, IF did not change NSC proliferation nor maintenance (Figs 6E and F, and EV2I and J). Other stages of neurogenesis were also unaffected since there were the same number of IPCs and neuroblasts in control an IF mice (Fig 6G and H).

We also noticed that in the previous work describing an increase in proliferation in the DG upon IF, a refeeding period before analysis had been introduced (Dias *et al*, 2021). To test whether this could lead to increased proliferation and neuroblast production, we performed 3 months of IF followed (or not) by an ad‐libitum feeding period of 5 days, as previously described (Fig 6I). Our results show that neither IPC nor neuroblast numbers were changed by IF respect to control mice, regardless of the refeeding period (Fig 6J and K).

Together, these results show that differences in sex, labelling method, strain, tamoxifen usage and diet length do not explain the discrepancies in the response of adult neurogenesis upon IF between our study and others. We conclude that every‐other day IF is not a robust strategy to increase adult neurogenesis in the hippocampus.

## Discussion

Fasting is a promising non‐pharmacological intervention to increase life span and counteract ageing that has been associated with increased adult neurogenesis. However, few studies have investigated the specific effects of fasting on adult NSCs and other adult stem cell populations. This is important not only because impaired adult stem cell function is regarded as one of the hallmarks of ageing, but also because alterations in the adult stem cell population will have long‐lasting consequences for tissue homeostasis. Here, we used genetic lineage tracing to determine the effects of every‐other‐day IF on adult NSCs.

We found that IF does not affect the proliferation or maintenance of adult NSCs. This suggested that NSCs are preserved upon IF and prompted us to investigate which further steps of neurogenesis might be increased by fasting. We carefully monitored the progression of the neurogenic lineage upon 1 or 3 months of IF and found no differences between control and fasted mice. This is in contrast with previous work reporting a sharp increase in proliferative cells (BrdU labelled) in the DG after 3 months of every‐other‐day IF (Dias *et al*, 2021). Alternating fasting periods with normal feeding has been proposed to be a crucial mechanism by which IF and CR exert their beneficial effects (Brandhorst *et al*, 2015). Dias *et al* analysed the mice after 5 days of a recovery period with *ad libitum* feeding, so we used the same protocol in our mice. Our data show that neither returning to *ad libitum* feeding from IF nor fasting on its own can promote cell proliferation along the neurogenic lineage. In addition, an accompanying report in this issue of *EMBO Reports* using a similar intervention consisting of two fasting days per week (known as 5:2 diet) shows no effect on proliferation in the DG, similar to our results, even after a refeeding period (Roberts *et al*, 2023).

Our results were therefore in line with other reports which did not find differences in cell proliferation upon fasting (Lee *et al*, 2002a,b; Kitamura *et al*, 2006). Those studies found instead an increased survival of newly born cells 4 weeks after fasting. We used lineage tracing to quantify the neuronal output of hippocampal NSCs and found no difference between control mice and mice fasted for 3 months. Classically, the effects of IF on adult neurogenesis were evaluated through neuronal survival assays with thymidine analogues (Lee *et al*, 2002a; Kim *et al*, 2015). Thymidine analogues (such as BrdU, CldU, IdU or EdU) are administered to the mice to label proliferating cells, with neurogenesis being evaluated after a chase period by direct quantification of the amount of newly born cells and/or comparison to the initially labelled population. This method evaluates only narrow waves of neurogenesis and, when several time points are used, can be useful to identify changes in the effects of the intervention over time. However, it also increases the variability of the results and, unlike lineage tracing, does not provide information on the total number of new neurons produced during the whole intervention. We nevertheless performed a classical label retention experiment to our mice and again found no increase in neuronal production upon IF for 3 or 4 months. Of note, we observed high variability in the effects of IF on adult neurogenesis, with an initial slight and non‐significant decrease followed by no effect and a final decrease in newly born cells. The effects we observed were small and only emerged when analysing small cohorts of new neurons, either using label retention or short (1‐month) lineage tracing. They could therefore be a consequence of the high degree of intrinsic variability in adult neurogenesis, questioning their functional importance. Since other cells proliferate in the dentate gyrus and can uptake the label (e.g. astrocytes, oligodendrocyte precursors or microglia), the interpretation of label‐retention data is challenging unless combined with neuronal markers. We indeed observed more pronounced changes in the numbers of EdU^+^ than EdU^+^NeuN^+^ cells upon IF. In addition, the thymidine analogue positive population by the end of the labelling period depends not only on uptake of the label by proliferating cells but also on their differentiation and survival (Kronenberg *et al*, 2003), hampering the interpretation of the results. By using lineage tracing combined with immunofluorescence and thymidine analogues in label‐retention experiments, we could separately evaluate different steps along the neurogenic lineage and faithfully quantify changes—or the lack of them—in neurogenic output.

To perform lineage tracing of adult NSCs, we used GlastCre mice that have a mixed genetic background. However, C57BL6/J is the most commonly used strain in fasting studies. In accordance with different strains presenting different levels of adult neurogenesis (Goodrick *et al*, 1990; Kempermann *et al*, 2006; Koehl, 2015; Kim *et al*, 2017; Wiget *et al*, 2017), we did observe differences in the number of neuroblasts between control GlastCre mice and control C57BL/6J at 5 months of age (Appendix Fig S2). Therefore, we repeated our experiments using C57BL/6J mice. Our results again showed that 3 or 4 months of fasting did not increase neurogenesis in the hippocampal niche (Figs 5F–I and 6A–H). However, since variations can occur even within the C57BL/6J strain and the response to dietary interventions is strain and even substrain‐specific (Hwang *et al*, 2008; Mitchell *et al*, 2016; Siersbæk *et al*, 2020; Bachmann *et al*, 2022), we cannot rule out that small differences in the animals' background could have contributed to the discrepancies between previous studies and ours.

Differences in the metabolic response to dietary interventions have also been observed between male and female mice (Mitchell *et al*, 2016; Bachmann *et al*, 2022), making it crucial to include both sexes when analysing the effects of any potential clinically relevant interventions. Although increased neurogenesis upon fasting has been reported for both sexes, we wondered whether differences in basal levels and response to the diet could be masking the effects of fasting in our data. Splitting our data by sex showed no increase in adult neurogenesis upon fasting in either male of female mice. We observed one parameter that was affected by sex: the number of new neurons after 1 month of IF (Figs 5A and B, and EV4). Since sex differences are particularly important for stress‐related factors (Yagi & Galea, 2019), we hypothesise this discrepancy could be linked to metabolic or stress‐related differences in handling the change from *ad libitum* feeding to fasting between male and female mice. Of note, while female mice are often excluded arguing higher variability in the results, variability was comparable in both sexes in our data (Fig EV4).

Although some reports indicate that weight loss is not necessarily linked to the benefits of IF and CR, it remains plausible that the degree of caloric restriction and weight loss induced by IF could influence its subsequent effects on adult neurogenesis. Both Lee *et al* and Kim *et al* reported weight loss upon every‐other‐day IF. Our protocol, in contrast, had little effect on the final weight of the mice or their growth curve. This is despite a reduction in calorie consumption of more than 20% and the presence of clear weight oscillations between fasting and feeding days. Our mice also showed signs of broad systemic changes induced by fasting (like changes in glucose and hydroxybutyrate levels, inguinal adipose tissue browning and liver remodelling), but we cannot rule out that these were not strong enough to induce changes in adult neurogenesis.

While establishing our every‐other‐day IF protocol, we found that daytime IF disrupts the circadian activity pattern of mice, introducing a confounding variable to IF that could affect adult neurogenesis (Bouchard‐Cannon *et al*, 2013; Draijer *et al*, 2019; Schouten *et al*, 2020). A very recent report supports this notion, as circadian entraining can boost the beneficial effects of caloric restriction on longevity (Acosta‐Rodríguez *et al*, 2022). This calls for a close monitoring of potential circadian disruptions during fasting interventions and for careful reporting of fasting protocols. However, circadian disruption alone does not explain the differences between our results and previous studies, as both day and night‐time IF have been shown to increase neurogenesis in young, healthy mice (M.  Mattson and S. Thuret, personal communication).

There are multiple other variables which can modulate the impact of fasting on neurogenesis. These include mice stress and overall fitness (Snyder *et al*, 2011; Toda *et al*, 2019), pathological states (Wu *et al*, 2008; Li *et al*, 2020; Cao *et al*, 2022), age (Park *et al*, 2013; Brandhorst *et al*, 2015) and even cage bedding (Gregor *et al*, 2020) and diet composition (Cryan & Dinan, 2012; Ribeiro *et al*, 2020; Wei *et al*, 2021). While we matched the age of the mice with previous reports and saw no striking changes in stress levels (measured by corticosterone), many other variables remain untested that could explain discrepancies between labs. We also did not assess differences in behavioural performance in our mice, which could be very interesting to determine the involvement of adult neurogenesis in the cognitive benefits of fasting. Of note, an accompanying study in this issue *of EMBO Reports* showing no effect on adult neurogenesis upon a 5:2 fasting intervention in mice also showed a lack of cognitive improvement in those mice (Roberts *et al*, 2023).

In summary, the specific combination of sex, strain, health status, housing conditions and fasting protocol can influence the outcome of dietary interventions on adult neurogenesis, putting a question mark on IF as a strategy to boost neurogenesis. Despite this, diet is regarded as an important regulator of adult neurogenesis (Aimone *et al*, 2014; Urbán *et al*, 2019; Denoth‐Lippuner & Jessberger, 2021), with fasting often highlighted as a positive modulator of neuronal production (Mattson, 2003; Levenson & Rich, 2007; Park & Lee, 2011; Zainuddin & Thuret, 2012; Fusco & Pani, 2013; Murphy *et al*, 2014; Bouchard & Villeda, 2015; Pani, 2015; Valero *et al*, 2016; Van Cauwenberghe *et al*, 2016; Wahl *et al*, 2016; de Lucia *et al*, 2017; Poulose *et al*, 2017; Katsimpardi & Lledo, 2018; Landry & Huang, 2021). The effects of fasting on adult neurogenesis are, however, very diverse, ranging from variable positive impact on the number of newly generated neurons (Lee et al, 2000, 2002a,b; Kitamura *et al*, 2006; Wu *et al*, 2008; Kumar *et al*, 2009; Park *et al*, 2013; Brandhorst *et al*, 2015; Kaptan *et al*, 2015; Kim *et al*, 2015; Apple *et al*, 2019; Baik *et al*, 2020; Li *et al*, 2020; Dias *et al*, 2021; Cao *et al*, 2022) to no effect or even a mild impairment (Bondolfi *et al*, 2004; Staples *et al*, 2017; Roberts *et al*, 2023, and our own study). We propose the variability is due to a combination of different factors (and most likely different combinations of factors in each case). This fits with a model in which IF can render NSCs and other cells along the neurogenic lineage more susceptible to further interventions such as circadian disruption or sudden re‐feeding (Urbán, 2022). In addition, publication bias might be playing a role in skewing the literature on fasting and neurogenesis towards reporting positive results. In some reviews, even studies reporting no effect are cited as evidence for improved neurogenesis upon IF. Reporting of negative results, especially those challenging accepted dogmas, and a careful and rigorous evaluation of the publications cited in reviews are crucial to avoid unnecessary waste of resources and to promote the advancement of science.

## Materials and Methods

### Animal husbandry

Mice were housed in groups in ventilated cages (TECNIPLAST 1285L NEXT) provided with HEPA filtered air covered with Aspen‐wood‐derived bedding (Kliba Nafag, ABEDD 4063 OM G10). They were kept under a 12:12 h light/dark cycle (light period 0600–1800 h CET), unless stated otherwise. Mice had *ad libitum* access to food (Ssniff, V1184‐300) and water. To generate a mouse line that allowed lineage tracing of adult NSCs with a fluorescent reporter (Andersen *et al*, 2014), we crossed Glast‐CreER^T2^ (Slc1a3^tm1(cre/ERT2)Mgoe^, Mori *et al*, 2006) mice and RYFP (Gt(ROSA)26Sor^tm1(EYFP)Cos^, Srinivas *et al*, 2001) mice. All Glast‐CreER^T2^;RYFP mice were of mixed genetic background. 4‐ to 6‐week‐old C57BL/6J (Charles River) mice were obtained from the Comparative Medicine facility at IMBA/IMP. Experimental groups were formed by randomly assigning littermates that were kept housed in groups of two to four mice per cage (except for the metabolic profiling of night‐time IF mice, where mice were single housed). To reach the desired number of mice per experiment, multiple litters were used. All experiments were conducted in male and female mice, except stated otherwise.

All animals were bred and maintained in accordance with ethical animal licence protocols complying with Austrian and European legislation.

### Intermittent fasting

Mice were randomly divided into Control or IF groups at postnatal day 65 (P65). Control mice had *ad libitum* access to food. Mice in the IF group were subjected to an every‐other‐day fasting regime, comprising 1 day of fasting followed by 1 day of feeding. For night‐time IF, food was removed at 1800 h CET coinciding with the time at which the lights went off (ZT12) and reintroduced at 1800 h CET on the following day (after 24 h). For daytime IF, food change happened every day at 0900 h CET (ZT3). Mouse weight was recorded every 3 days in both Control and IF groups and leftover food weighted once per week. Weight gain was calculated as the percentage of the weight difference to the weight at the beginning of the diet (P65). Total food intake was calculated by the difference between supplied and leftover food over the treatment in each cage and divided by the number of animals per cage. The length of each experiment is specified in the text and figure captions. For the 4‐month‐long experiment and the refeeding experiment depicted in Fig 6, mice were fed automatically on the defined schedule through an internally developed automated system.

### Automated home cage mouse phenotyping

Locomotor and metabolic activity were measured using the PhenoMaster system TSE Systems every 15 min during a month. Mice were acclimated to PhenoMaster water bottles for at least 6 days and to the complete PhenoMaster housing (TECNIPLAST GreenLine GM 500) for 2 days. Mice in the daytime IF experiment were housed in groups (two to three mice per cage) in a 14:10 h light/dark cycle (light period 0600–2000 h CET). Mice in the night‐time IF experiment were housed individually in a 12:12 h light/dark cycle (light period 0600–1800 h CET). Cages were kept in a climate chamber in the same environmental conditions as the general animal rooms (24°C and 50% humidity). The PhenoMaster cages were programmed to automatically grant or restrict access to the food basket at the designated time.

Before each run, CO_2_ and O_2_ sensors were calibrated against a defined mix of CO_2_ and O_2_. The respiratory exchange ratio (RER) was calculated as the ratio of VCO_2_ produced to the VO_2_ consumed by the PhenoMaster software. Mouse locomotor activity was monitored using the ActiMot2 Activity module equipped with 3‐dimensional infrared sensors. An estimate of movement (DistD, cm) was calculated using the PhenoMaster software. Data acquired in 15‐min intervals are expressed as means of 48 h cycles. For grouped housed mice (daytime IF), DistD was divided by the number of mice in each cage. Total mouse locomotor activity was calculated as the sum of cm throughout the whole intervention and segregated by time periods to depict differences between diurnal and nocturnal behaviour during fasting and feeding days. For daytime IF, where the mice were kept in a 14:10 LD cycle, the data was divided in ZT 0–14–24–38–48 h. For night‐time IF, where the mice were kept in a 12:12 LD cycle, the data were divided in ZT 0–12–24–36–48 h.

The automated home cage mouse phenotyping was performed by the Preclinical Phenotyping Facility at Vienna BioCenter Core Facilities (VBCF), member of the Vienna BioCenter (VBC), Austria.

### Tamoxifen and EdU administration

To induce activation of CreER^T2^ recombinase, Glast‐CreER^T2^;RYFP mice were intraperitoneally injected with 2 mg (75–100 mg/kg) of Tamoxifen (Sigma, T5648) diluted in corn oil (Sigma, C8267) at postnatal day 60 (P60) for 5 consecutive days. Recombination levels were evaluated by comparing YFP^+^ neuroblasts (DCX^+^ cells) or NSCs to their total respective numbers (Fig EV2). Recombination was very high in both experiments (1 and 3 months—above 90%) and levels were comparable in Control and IF groups.

To label cells in S‐phase, 5‐ethynyl‐2′‐deoxyuridine (EdU, Carl Roth, 7845) was administered in drinking water (0.1 mg/ml) *ad libitum*. The length of EdU administration and chase period is specified in the text and figures.

### Tissue preparation and immunohistochemistry

Mice were transcardially perfused with phosphate buffered saline (PBS) for 3 min, followed by 4% paraformaldehyde (PFA, Sigma, P6148) in PBS for 15 min.

Brains were post‐fixed in 4% PFA for 2–6 h at 4°C with rocking, washed with PBS and stored in PBS with 0.02% Sodium Azide (ITW Reagents, A1430). Brains were coronally sectioned at a thickness of 40 μm using a vibratome (Leica, VT1000S). Immunofluorescence of brain tissue was performed in free‐floating sections. Sections were blocked in 1% Triton‐PBS with 10% normal donkey serum (DS, Jackson Immuno Research, JAC0170001210) for 2 h at room temperature. Primary and secondary antibody solutions were prepared in 0.1% Triton‐PBS with 10% DS. Sections were incubated in primary antibody solution at 4°C overnight, washed 3 × 15 min with 0.1% Triton‐PBS and incubated with secondary antibody solution at room temperature for 2 h. Following 1× 0.1% Triton‐PBS and 2× PBS 15 min washes, sections were incubated with 1 μg/ml DAPI (Sigma, D9542) in 1:1 PBS:H_2_O at room temperature for 30 min. EdU was detected before DAPI incubation using Click‐iT™ Plus Alexa Fluor™ Picolyl Azide tool kits with 488 and 647 dyes (Invitrogen, C10641 and C10643 respectively). All incubations and washes were performed under gentle rocking.

Inguinal adipose tissue and livers were dissected and post‐fixed in 4% PFA at room temperature with rocking for 24 h prior to paraffin embedding. Eight random mice per condition were chosen from the original cohort of mice for immunohistochemical analysis. Fixed tissue was process in a Donatello Automatic Sample Processor (Diapath) and embedded in paraffin. 2 μm thick sections were collected on glass slides. For staining of the inguinal adipose tissue antigen retrieval with tri‐sodium citrate (pH = 6) was carried out for 30 min prior to a 1‐h‐incubation with the primary antibody at room temperature. The two steps HRP polymer Kit with DAB (DCX, PD000POL‐K) was used for detection followed by haematoxylin counterstaining. Liver sections were stained with haematoxylin and eosin in an Erpedia Gemini AS strainer (Fisher Scientific).

Primary and secondary antibodies and concentrations are listed in Table EV1. Processing and staining of adipose tissue and liver was performed by the Histology facility at the VBCF.

### Imaging

Images of brain immunofluorescence were acquired using an inverted Axio Observer microscope (ZEISS) equipped with a CSU X1 confocal scanning unit (ZEISS) and an EM‐CCD camera (Hamamatsu, C9100‐13). 40× and 63× oil Plan‐Apochromat objective lenses (ZEISS) were used to image through whole 40‐μm sections with a z‐step of 1 μm. Images were stitched using ZEN Blue software (ZEISS).

Images of adipose tissue and liver were acquired using a Pannoramic FLASH 250 II (3DHISTECH) equipped with a CIS VVC FC60FR19CL camera (Vital Vision Technology) and a 20× Plan‐Apochromat objective. Representative images were cropped using the CaseViewer software.

### Image quantifications

Quantifications of cells in the neurogenic lineage were performed using the Fiji software (Schindelin *et al*, 2012) with the Cell Counter plugin. NSCs were identified by the localisation of a DAPI^+^ nucleus in the SGZ, the extension of a single GFAP^+^ vertical projection towards the molecular layer and the help of the YFP lineage tracing reporter that is brighter in NSCs than other cells. In Fig EV2, NSCs were identified by a DAPI^+^Sox2^+^ nucleus in the SGZ and the extension of a single GFAP^+^Nestin^+^ vertical projection towards the molecular layer. In C57BL/6J mice, NSCs were identified by GFAP, localisation and morphology. For the IPCs were identified as Ki67^+^ cells in the SGZ. Neuroblasts were identified as DCX^+^ cells in the SGZ. Newly born neurons were identified as NeuN^+^ cells that colocalised with YFP (in GlastCreER^T2^;RYFP mice) or EdU (in C57BL/6J mice) in the SGZ and granule cell layer of the DG. In GlastCreER^T2^;RYFP mice, NeuN counts were normalised to the recombination rate. New cells in the DG were identified by EdU labelling. For cell numbers, 1 dorsal section of DG per sample was quantified (two for NSCs) between bregma −1.82 and −2.18. The freehand line tool was used to measure the length of the SGZ, to which the total cell counts were normalised. For percentage of proliferating or EdU‐labelled NSCs, at least 120 cells from two DG were counted in young mice, and 70 in older mice. All data were counted with group blinding within each experiment.

For stereological counts of EdU^+^ cells in the whole DG (Figs 5G and 6C), one in six 40‐μm‐thick serial sections containing the DG were stained and analyzed. Because of the low number of EdU^+^ cells, the quantification was performed in the whole DG area of each section (consisting of SGZ and granule cell layer). The average of both hemispheres was used to calculate the number of cells in one DG per section and the total number of cells in one whole DG (counted sections * 6). Because of occasional tissue damage in the most caudal DG areas due to vibratome glue, some samples were not complete along the rostro‐caudal axis. Only those with all sections were used for whole DG counts, while all samples are displayed in the rostro‐caudal axis representation.

The appropriate sample size was based on previous publications studying NSC behaviour (Andersen *et al*, 2014; Urbán *et al*, 2016; Blomfield *et al*, 2019; Austin *et al*, 2021), accounting for the increased variability of dietary interventions compared to genetic ones and considering female and male mice as separate groups. For the quantifications based on the YFP reporter, samples with a recombination rate lower than 80% were excluded. Samples with poor staining quality were also excluded from the quantifications.

### Serological analysis

Blood was collected at ZT12 (time of lights OFF) through cardiac puncture into serum separating tubes (SST™ tubes, BD Microcontainer, 365968) and allowed to clot for 30 min at room temperature. Samples were centrifuged at 2,000 *g* for 20 min at 4°C and the serum transferred to a fresh tube and stored at −20°C until subsequent analysis.

Metabolite extracts were generated by adding 60 μl of methanol to 20 μl of serum in a 1.5 ml tube. Following addition of 60 μl acetonitrile, tubes were centrifuged at 20,000 *g* and the supernatants were used for both hydrophilic interaction liquid chromatography (HILIC), and reversed phase analysis (RP) directly coupled to tandem mass spectrometry (LC–MS/MS). RP‐LC–MS/MS was performed using an Ultimate 3000 HPLC system (Dionex, Thermo Fisher Scientific) and a TSQ Altis mass spectrometer (Thermo Fisher Scientific). 1 μl of each sample was injected onto a Kinetex (Phenomenex) C18 column (100 Å, 150 × 2.1 mm) connected with the respective guard column, and employing a 5‐min‐long linear gradient from 97% A (1% acetonitrile, 0.1% formic acid in water) to 95% B (0.1% formic acid in acetonitrile) at a flow rate of 80 μl/min. Employing selected reaction monitoring (SRM), corticosterone was quantified with the transition 347.1–121.1 *m*/*z*, in the positive ion mode. In HILIC, 1 μl of each extract was injected onto a polymeric iHILIC‐(P) Classic HPLC column (HILICON, 100 × 2.1 mm; 5 μm) and the respective guard column, operated at a flow rate of 100 μl/min. A linear gradient (A: 95% acetonitrile 5%, 5 mM aqueous ammonium bicarbonate; B: 5 mM aqueous ammonium bicarbonate, supplemented with 0.1 μg/ml medronic acid) starting with 13% B and ramping up to 90% B in 8 min was used for separation. Using a TSQ Quantiva mass spectrometer (Thermo Fisher Scientific), the following SRM transitions were used for quantitation in the negative ion mode: 103.1–59 *m*/*z* (hydroxybutyrate) and 179–59 *m*/*z* (glucose).

Authentic standards (Supelco Corticosterone #46148 VETRANAL^®^; glucose #G8270 and 3‐hydroxybutyric acid #166898 Merck, Sigma‐Aldrich) were used for determining optimal collision energies of the SRM transitions and for validating experimental retention times via standard addition to a pooled quality control sample. The data interpretation was performed using TraceFinder (Thermo Fisher Scientific).

### Statistical analysis

All statistical analyses were conducted using GraphPad Prism version 9.3.1 for macOS (GraphPad Software, San Diego, California, USA). Two‐tailed unpaired student *t*‐tests were used for the comparison of two conditions. Whenever the samples did not pass a Shapiro–Wilk normality test (*P* < 0.05), the non‐parametric Mann–Whitney test was conducted (Fig EV2A–D). A two‐way ANOVA for repeated measures and multiple comparisons with a Šídák correction was performed to compare the weight of control and IF mice over time in Fig 1C. In Fig EV1L, the weight gain data had to be fitted into a mixed effects model because of a missing value. For sex comparisons, a two‐way ANOVA followed by Tuckey's multiple comparisons test was performed. Error bars represent average + standard deviation (s.d.) unless stated otherwise. *P*‐values are specified in figure captions or Appendix Tables S1–S7. Significance is summarised as follows: ns, *P* > 0.05; *, *P* < 0.05; **, *P* < 0.01; ***, *P* < 0.001; ****, *P* < 0.0001. In graphs displaying weight (Figs 1C and EV1G) and in Figs EV1A and B, and EV4 *P* > 0.05 is indicated by absence of significance symbol. Independent biological replicates are represented as dots in the bar plots.

## Author contributions

**Rut Gabarró‐Solanas:** Conceptualization; data curation; formal analysis; funding acquisition; validation; investigation; visualization; methodology; writing – original draft; writing – review and editing. **Amarbayasgalan Davaatseren:** Data curation; formal analysis; investigation; writing – review and editing. **Justus Kleifeld:** Data curation; formal analysis; investigation; writing – review and editing. **Tatjana Kepčija:** Data curation; formal analysis; investigation; writing – review and editing. **Thomas Köcher:** Data curation; formal analysis; writing – review and editing. **Albert Giralt:** Data curation; formal analysis; writing – review and editing. **Iván Crespo‐Enríquez:** Data curation; formal analysis; investigation; writing – review and editing. **Noelia Urbán Avellaneda:** Conceptualization; resources; formal analysis; supervision; funding acquisition; investigation; writing – original draft; project administration; writing – review and editing.

## Disclosure and competing interests statement

The authors declare that they have no conflict of interest.

## Supporting information



Appendix S1

Expanded View Figures PDF

Table EV1

Dataset EV1

Review Process File

PDF+

Source Data for Figure 1

Source Data for Figure 2

Source Data for Figure 3

Source Data for Figure 4

Source Data for Figure 4

## Data Availability

This study includes no data deposited in external repositories.
