# Supplementary material for: Adult neural stem cells and neurogenesis are resilient to intermittent fasting
Source: EMBO Rep. 2023 Nov 21;24(12):e57268. doi: 10.15252/embr.202357268 (PMC10702802; doi:10.15252/embr.202357268)
Supplement: Supplementary file 1 — Appendix S1 [file EMBR-24-e57268-s003.pdf]

Appendix

Adult neural stem cells and neurogenesis are resilient to intermittent fasting

Rut Gabarró-Solanas[1,2], Amarbayasgalan Davaatseren[1†], Justus Kleifeld[1,2†], Tatjana Kepčija[1], Thomas Köcher[3], Albert Giralt[4,5,6,7], Iván Crespo-Enríquez[1] and Noelia Urbán[1,*]

[1]Institute of Molecular Biotechnology of the Austrian Academy of Sciences (IMBA), Vienna BioCenter (VBC), Dr. Bohr-Gasse 3, 1030 Vienna, Austria.
[2]Vienna BioCenter PhD Program, Doctoral School of the University of Vienna and Medical University of Vienna, A-1030, Vienna, Austria.
[3]Vienna BioCenter Core Facilities, Campus-Vienna-Biocenter 1, Vienna, Austria.
[4]Departament de Biomedicina, Facultat de Medicina, Institut de Neurociències, Universitat de Barcelona, Casanova 143, 08036 Barcelona, Spain.
[5]Institut d'Investigacions Biomèdiques August Pi i Sunyer (IDIBAPS), 08036 Barcelona, Spain.
[6]Centro de Investigación Biomédica en Red Sobre Enfermedades Neurodegenerativas (CIBERNED), 08036 Barcelona, Spain.
[7]Production and Validation Center of Advanced Therapies (Creatio), Faculty of Medicine and Health Science, University of Barcelona, 08036 Barcelona, Spain.
[†]Equal contribution.
*Correspondence: noelia.urban@imba.oeaw.ac.at

Table of Contents

Appendix Figures……………………………………………………………………………..2
Appendix Figure S1…………………………………………………………………2
Appendix Figure S2…………………………………………………………………3
Appendix Figure S3…………………………………………………………………3
Appendix Tables…………………………………………………………………………..4
Appendix Table S1……………………………………………………………………4
Appendix Table S2……………………………………………………………………4
Appendix Table S3……………………………………………………………………4
Appendix Table S4……………………………………………………………………4
Appendix Table S5……………………………………………………………………5
Appendix Table S6……………………………………………………………………5
Appendix Table S7……………………………………………………………………5

Appendix Figure S1

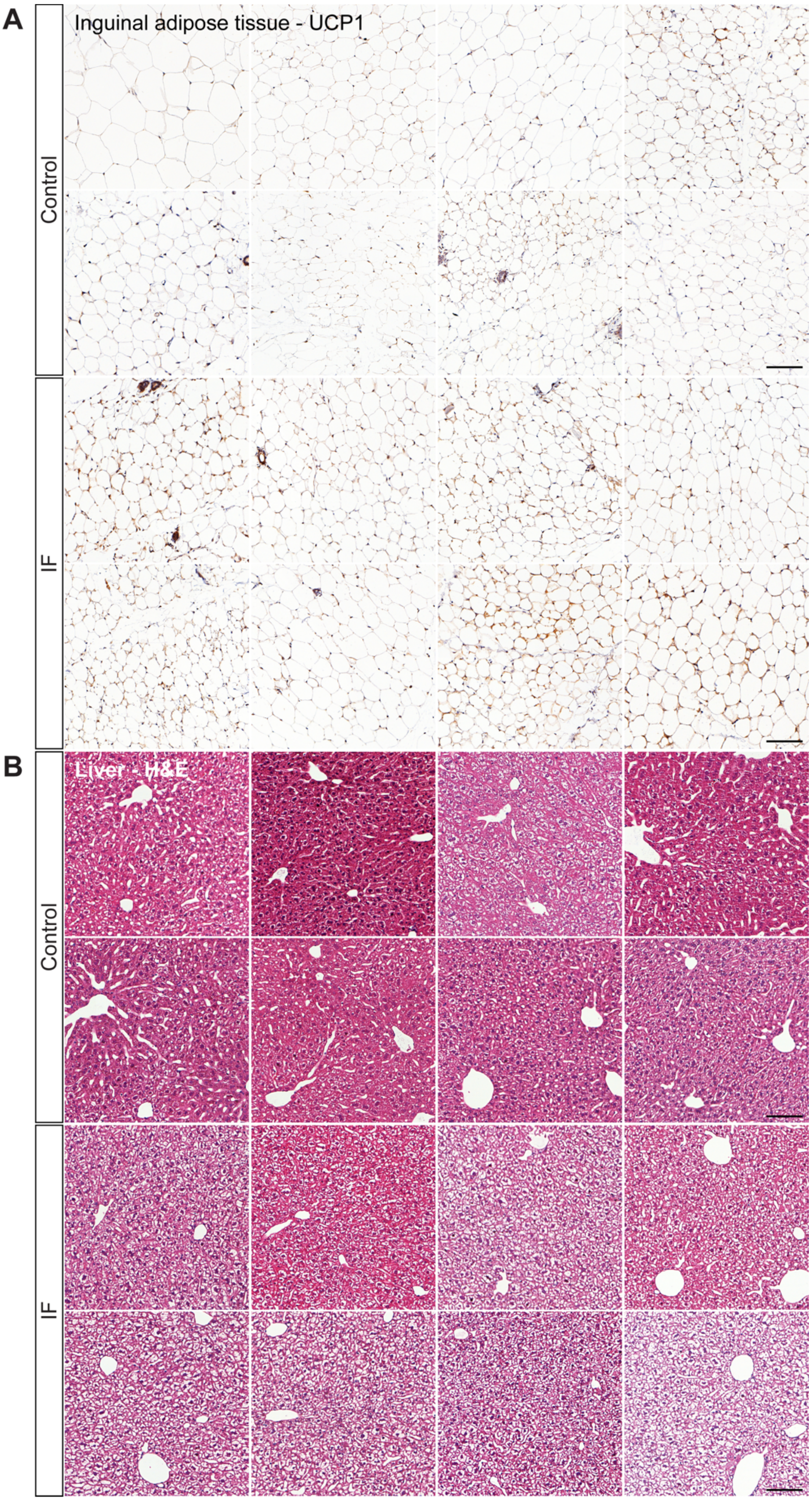

Appendix Figure S1. Images of all samples used for evaluation of adipose browning and liver remodelling. (**A**) Night-time IF promotes mild and variable browning of inguinal adipose tissue. Images of inguinal adipose tissue stained for uncoupling protein 1 (UCP1) of control and mice that underwent 3 months of IF.

Legend continues on next page.

(**B**) Hepatocytes display edematous morphology upon night-time IF. Images of H&E-stained liver of control and mice that underwent 3 months of IF.

Data information: 8 mice per condition were randomly chosen from the experiment shown in **Figure 3** for immunohistochemical evaluation. Each image corresponds to an individual mouse including those shown in **Figure 1J, K**. Scale bars: 100µm.

Appendix Figure S2

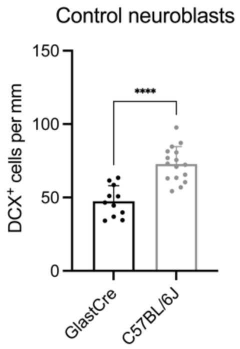

Appendix Figure S2. Differences in basal levels of neurogenesis between mouse strains.

Neuroblasts (DCX+ cells) normalised to DG length per 40 µm thick section in Glast-CreERT2;RYFP and C57BL/6J mice. The neurogenic rate (shown as number of neuroblasts) is a 40% lower in 5-month-old Glast-CreER^{T2};RYFP mice with a mixed genetic background than in C57BL/6J mice of matched age. $n_{GlastCre}$=11, $n_{C57BL6/J}$=16. Two-tailed unpaired t-test, p<0.0001. Bars and error bars represent average + s.d., dots represent individual mice.

Appendix Figure S3

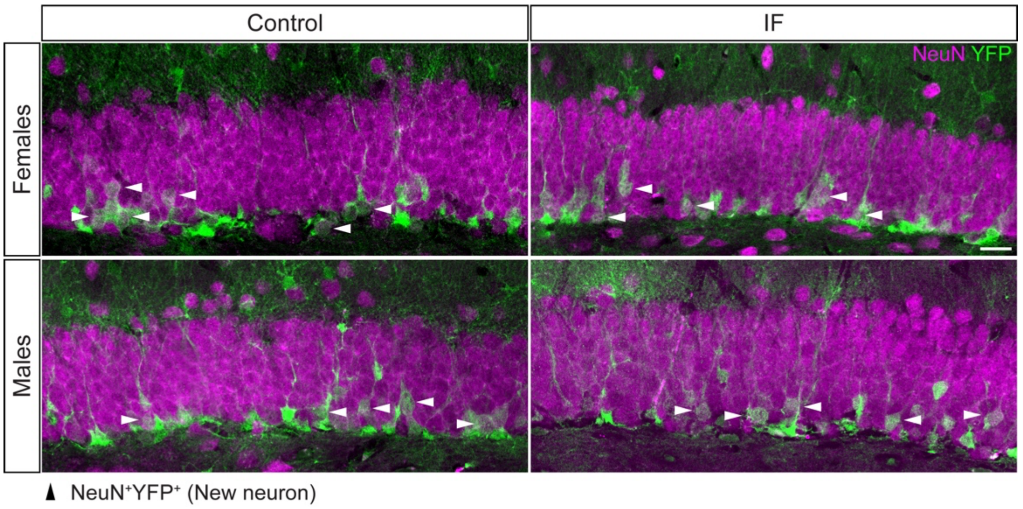

Appendix Figure S3. New neurons segregated by sex.

Images of newly born neurons (arrowheads) in male and female mice after 3 months of IF. Neurons were identified by the colocalization of the mature neuronal marker NeuN and the YFP reporter, indicating that these neurons were generated from the population of NSCs labelled with YFP at the beginning of the diet. Images from female mice correspond to those shown in **Figure 4D**. Scale bar: 20µm.

Appendix Table S1: Statistics (p-values) for Figure 4

Comparison	Test	Figure 4B	Figure 4D	Figure 4F
1 month:Control vs. 1 month:IF	Tuckey's multiple comparisons	0.3419	0.8764	0.7228
1 month:Control vs. 3 months:Control		<0.0001	<0.0001	<0.0001
1 month:Control vs. 3 months:IF		<0.0001	<0.0001	<0.0001
1 month:IF vs. 3 months:Control		<0.0001	<0.0001	<0.0001
1 month:IF vs. 3 months:IF		0.0007	<0.0001	<0.0001
3 months:Control vs. 3 months:IF		0.4195	>0.9999	0.9993

Appendix Table S2: Statistics (p-values) for Figure 5

Comparison	Test	Figure 5A	Figure 5B
Females:Control vs. Females:IF	Tuckey's multiple comparisons	0.5085	0.9875
Females:Control vs. Males:Control		0.7384	0.7961
Females:Control vs. Males:IF		0.0159	0.9129
Females:IF vs. Males:Control		0.9862	0.9188
Females:IF vs. Males:IF		0.1952	0.9829
Males:Control vs. Males:IF		0.1243	0.9942

Appendix Table S3: Statistics (p-values) for Figure EV1A and B

Comparison	Test	Figure EV1A	Figure EV1B
Interaction	Two-way ANOVA	0.0001	0.1794
Time/day		0.0085	<0.0001
Diet		0.3591	0.1794
C vs. IF: Feeding day	Šídák's multiple comparisons	0.9852	0.9994
C vs. IF: Fasting night		0.0764	0.2036
C vs. IF: Fasting day		0.0002	0.9417
C vs. IF: Feeding night		0.6015	0.3798

Appendix Table S4: Statistics (p-values) for Figure EV1G

Comparison	Test	Day 3	Day 6	Day 9	Day 12	Day 15
C vs. IF	Šídák's multiple comparisons	0.0001	0.9879	0.0001	0.7987	0.0302
		**Day 18**	**Day 21**	**Day 24**	**Day 27**	**Day 30**
		0.8353	0.3365	0.1114	0.5805	0.4729
		**Day 33**	**Day 36**	**Day 39**	**Day 42**	**Day 45**
		0.1083	0.9999	0.0104	0.9999	0.0288
		**Day 48**	**Day 51**	**Day 54**	**Day 57**	**Day 60**
		0.9999	0.2874	0.9999	0.0046	0.9999
		**Day 63**	**Day 66**	**Day 69**	**Day 72**	**Day 75**
		0.0002	0.9999	0.0023	0.9999	0.0007
		**Day 78**	**Day 81**	**Day 84**		
		0.9952	0.0001	0.9999		

Appendix Table S5: Statistics (p-values) for Figure EV4

Comparison	Test	Figure EV4A	Figure EV4B	Figure EV4C	Figure EV4D	Figure EV4E
Interaction	Two-way ANOVA	0.1449	0.0086	0.5967	0.623	0.9395
Sex		0.7684	0.4551	0.659	0.7919	0.4346
Diet		0.8517	0.9871	0.4492	0.5975	0.2039
		**Figure EV4F**	**Figure EV4G**	**Figure EV4H**	**Figure EV4I**	**Figure EV4J**
Interaction		0.5182	0.5568	0.5747	0.6541	0.6496
Sex		0.3228	0.3819	0.977	0.3495	0.6781
Diet		0.1024	0.0685	0.4906	0.9623	0.7145
		**Figure EV4K**	**Figure EV4L**			
Interaction		0.7581	0.5355			
Sex		0.8321	0.7421			
Diet		0.4295	0.9447			

Appendix Table S6: Additional statistics (p-values) for Figure EV4B

Comparison	Test	Figure EV4B
Females:Control vs. Females:IF	Tuckey's multiple comparisons	0.1392
Females:Control vs. Males:Control		0.0503
Females:Control vs. Males:IF		0.9552
Females:IF vs. Males:Control		0.9417
Females:IF vs. Males:IF		0.5246
Males:Control vs. Males:IF		0.2829

Appendix Table S7: Statistics (p-values) for Figure EV5

Comparison	Test	Figure EV5A	Figure EV5B
Interaction	Two-way ANOVA	0.0134	0.1296
Section		<0.0001	<0.0001
Diet		<0.0001	<0.0001
C vs. IF: Section 1	Šídák's multiple comparisons	>0.9999	0.1696
C vs. IF: Section 2		>0.9999	<0.0001
C vs. IF: Section 3		<0.0001	0.0002
C vs. IF: Section 4		0.0024	0.0002
C vs. IF: Section 5		0.0591	0.0002
C vs. IF: Section 6		0.0023	0.013
C vs. IF: Section 7		0.251	0.0167
C vs. IF: Section 8		0.0337	0.0374
C vs. IF: Section 9		0.9998	0.8
C vs. IF: Section 10		0.4996	0.9522
C vs. IF: Section 11		0.3671	
