# Supplementary material for: Adult neural stem cells and neurogenesis are resilient to intermittent fasting
Source: EMBO Rep. 2023 Nov 21;24(12):e57268. doi: 10.15252/embr.202357268 (PMC10702802; doi:10.15252/embr.202357268)
Supplement: Supplementary file 2 — Expanded View Figures PDF [file EMBR-24-e57268-s008.pdf]

Expanded View Figures

Figure EV1. Systemic effects of IF.

A, B Mouse locomotor activity in control (*ad libitum*, $n_{control\text{-}daytime} = 3$, $n_{control\text{-}night\text{-}time} = 6$), daytime IF (A, $n = 4$), and night-time IF (B, $n = 6$) mice displayed as sum of activity during 1 month of IF segregated by time of the day (grouped according to light phase). Black and white boxes under the graphs indicate respectively the dark and light phases of the light:dark (LD) cycle with zeitgeber time (ZT) 0 being the time of lights ON. Yellow/blue and white boxes indicate the presence and absence of food respectively. Expected differences between light and dark phases were observed. Daytime IF increased locomotor activity during the feeding day while night-time IF preserved the activity pattern. Two-way ANOVA followed by Šídák's multiple comparisons test. See significance values in Appendix Table S3.

C, D Total mouse locomotor activity during 1 month of daytime (C) or night-time (D) IF. Two-tailed unpaired *t*-test; (C) $n_{control\text{-}daytime} = 3$, $n_{IF\text{-}daytime} = 4$, $P = 0.5596$, (D) $n_{control\text{-}night\text{-}time} = 6$, $n_{IF\text{-}night\text{-}time} = 6$, $P = 0.3698$.

E, F Daily mouse activity (E) and energy expenditure (F) over time in control and night-time IF mice. Mice in the two conditions follow comparable trends except for daily activity during the first 3–4 days, when activity is more variable in night-time IF mice than in control mice.

G Mouse weight shown as the percentage of weight difference to the first day of the diet. Weight oscillations persist throughout the 3 months of IF and there is no weight loss on refeeding days compared to control mice. $n_{control} = 17$, $n_{IF} = 17$. Mixed-effects analysis $P_{time} < 0.0001$, $P_{diet} = 0.0215$, $P_{int} < 0.0001$; followed by Šídák's multiple comparisons test, see significance values in Appendix Table S4.

H Average total food consumed for 3 months shown as g of food per 20 g of mouse weight (weight reference of mice at the start of the diet, P65). $n_{control} = 17$, $n_{IF} = 17$. Two-tailed unpaired *t*-test, $P = 0.8333$.

I Monthly energy expenditure displayed as average heat per hour. $n_{control} = 6$, $n_{IF} = 6$. Two-tailed unpaired *t*-test, $P = 0.2141$.

J The serum of mice was obtained by cardiac puncture after 1 month of *ad libitum* eating (control) or IF. Different groups were used to collect serum after a fasting or feeding day.

K–M Targeted metabolomics were performed to determine the levels of glucose (K), hydrobutyric acid (L) and corticosterone (M) in the serum of mice. The area of ion counts is displayed. $n_{control} = 4$, $n_{IF(fasting\ day)} = 4$, $n_{IF(feeding\ day)} = 4$. One-way ANOVA, (K) $P = 0.0002$, (L) $P < 0.0001$, (M) $P = 0.0515$; followed by Tukey's multiple comparisons test when the ANOVA was significant, (K) $P_{control\text{-}IFfasting} = 0.0004$, $P_{control\text{-}IFfeeding} = 0.8032$, $P_{IFfasting\text{-}IFfeeding} = 0.0008$; (L) $P_{control\text{-}IFfasting} < 0.0001$, $P_{control\text{-}IFfeeding} = 0.7749$, $P_{IFfasting\text{-}IFfeeding} < 0.0001$.

Data information: Bars and error bars represent average + s.d., dots represent individual mice. Significance summary: ns, $P > 0.05$; *, $P < 0.05$; **, $P < 0.01$; ***, $P < 0.001$; ****, $P < 0.0001$.

© 2023 The Authors

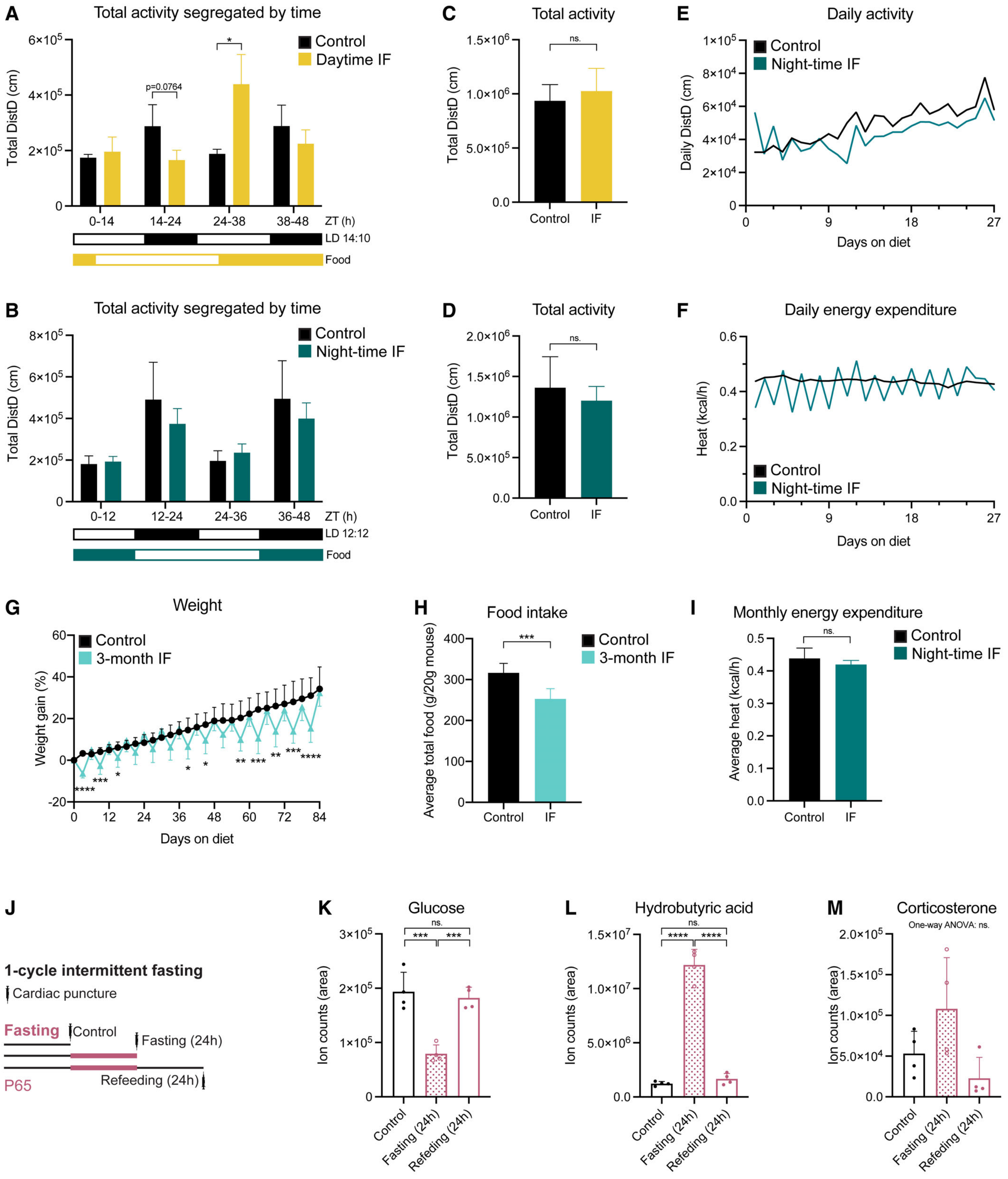

Figure EV1.

© 2023 The Authors

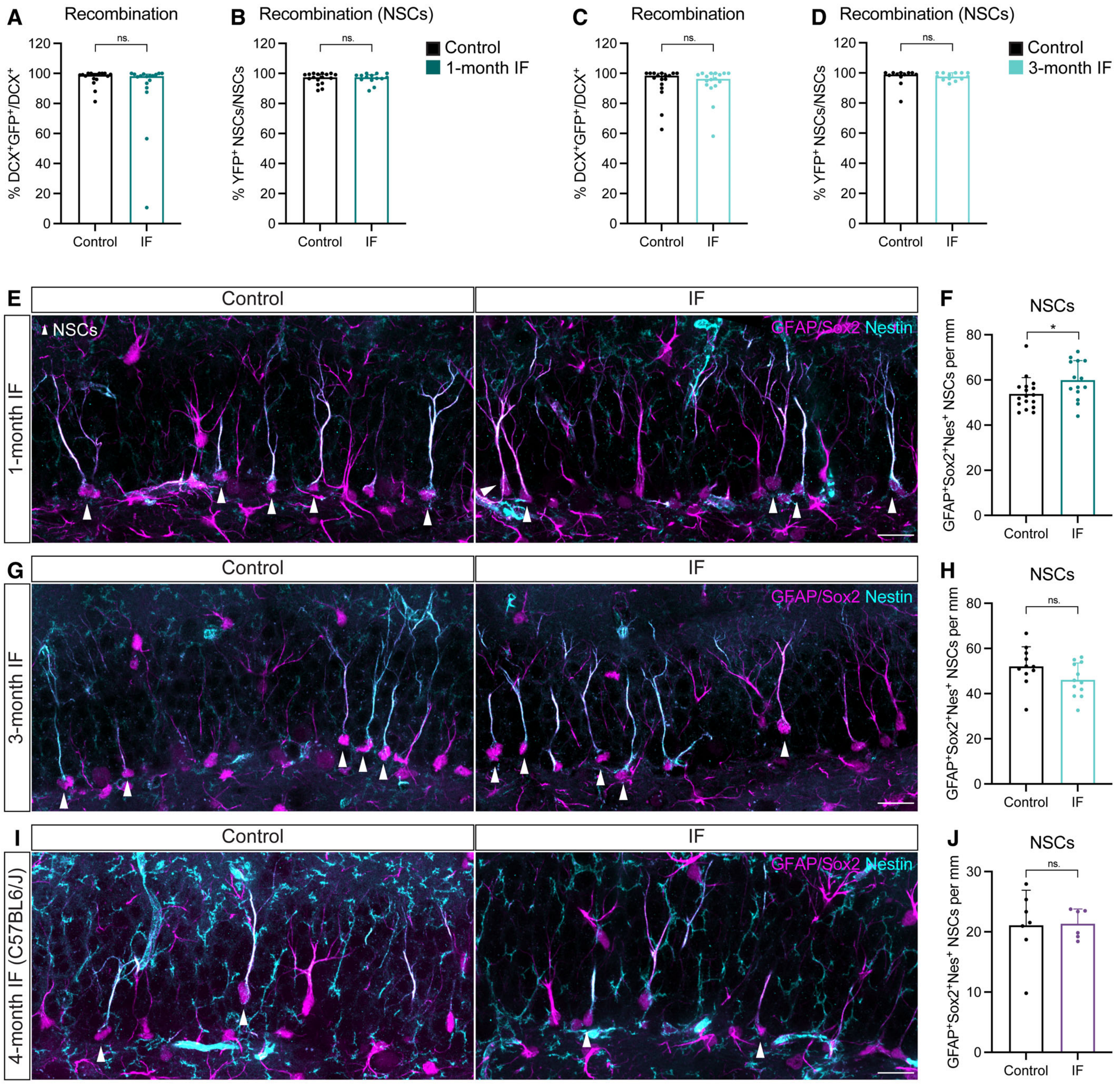

Figure EV2. High recombination in Glast-CreER^{T2};RYFP throughout conditions, and additional NSC analysis.

A–D Recombination of the YFP reporter in Glast-CreER^{T2};RYFP upon tamoxifen induction shown as YFP$^+$ neuroblasts (A, C) or NSCs (B, D) over their total numbers after 1 (A, B) or 3 (C, D) months of treatment. Only animals with a recombination rate higher than 80% were considered for further analysis. Mann–Whitney tests. (A) $n_{control} = 17$, $n_{IF} = 17$, $P = 0.3233$; (B) $n_{control} = 17$, $n_{IF} = 14$, $P = 0.7760$; (C) $n_{control} = 17$, $n_{IF} = 17$, $P = 0.7818$; (D) $n_{control} = 12$, $n_{IF} = 13$, $P = 0.5318$. Bars and error bars represent median + interquartile range.

E–J Images of NSCs (arrowheads) and quantifications normalised to DG length per 40-μm section in Glast-CreER^{T2};RYFP mice after 1 (E, F) or 3 (G, H) months of *ad libitum* (control) eating or IF, and C57BL6/J mice after 4 months of diet (I, J). NSCs were identified as cells with a Sox2$^+$ nucleus in the SGZ extending a single GFAP$^+$Nestin$^+$ radial projection to the molecular layer. (F) $n_{control} = 17$, $n_{IF} = 14$; (H) $n_{control} = 11$, $n_{IF} = 12$; (I) $n_{control} = 7$, $n_{IF} = 6$. Two-tailed unpaired *t*-tests; (F) $P = 0.0414$, (H) $P = 0.09$, (I) $P = 0.9243$. Bars and error bars represent average + s.d.

Data information: Control (*ad libitum*) for each experiment (black), 1-month IF (dark blue), 3-month IF (light blue), 4-months IF C57BL6/J (purple). Dots represent individual mice. Significance summary: ns, $P > 0.05$; *, $P < 0.05$.

© 2023 The Authors

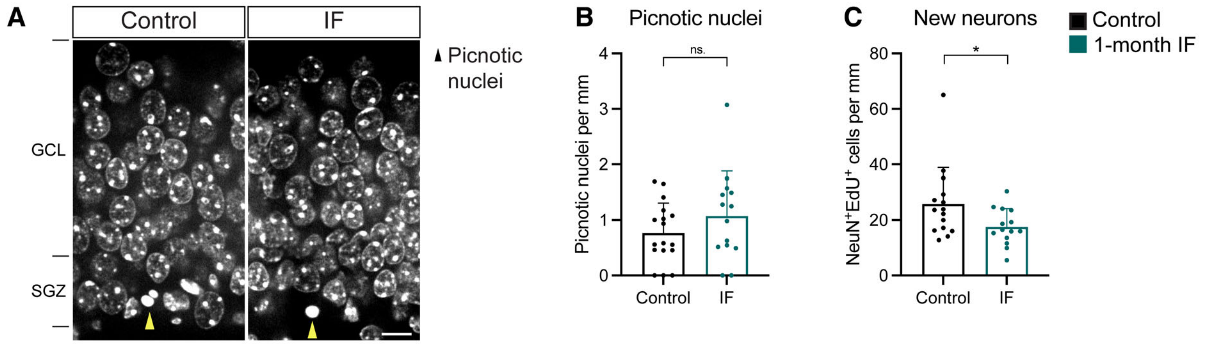

Figure EV3. Transient decrease in the generation of new neurons upon 1 month of IF. See experimental design in Fig 2A.

A Images of picnotic nuclei (yellow arrowheads) in the SGZ of the DG in control and IF mice after 1 month of diet.

B Quantification of picnotic nuclei normalised to DG length per 40-μm-thick section as a proxy for cell death, which was not affected by IF. $n_{control}$ = 17, n_{IF} = 14. Two-tailed unpaired t-test, P = 0.2172.

C Number of EdU-labelled neurons (as NeuN$^+$ cells) normalised to DG length per 40-μm-thick section after a 10-day chase, showing a decrease in the number of new neurons generated. Note that 10 days is shorter than the conventional 1 month of chase, which is why neuron numbers are still very low. $n_{control}$ = 15, n_{IF} = 14. Two-tailed unpaired t-test, P = 0.0427.

Data information: Bars and error bars represent average + s.d.; dots represent individual mice. Significance summary: ns, $P > 0.05$; *, $P < 0.05$. Scale bar: 10 μm.

© 2023 The Authors

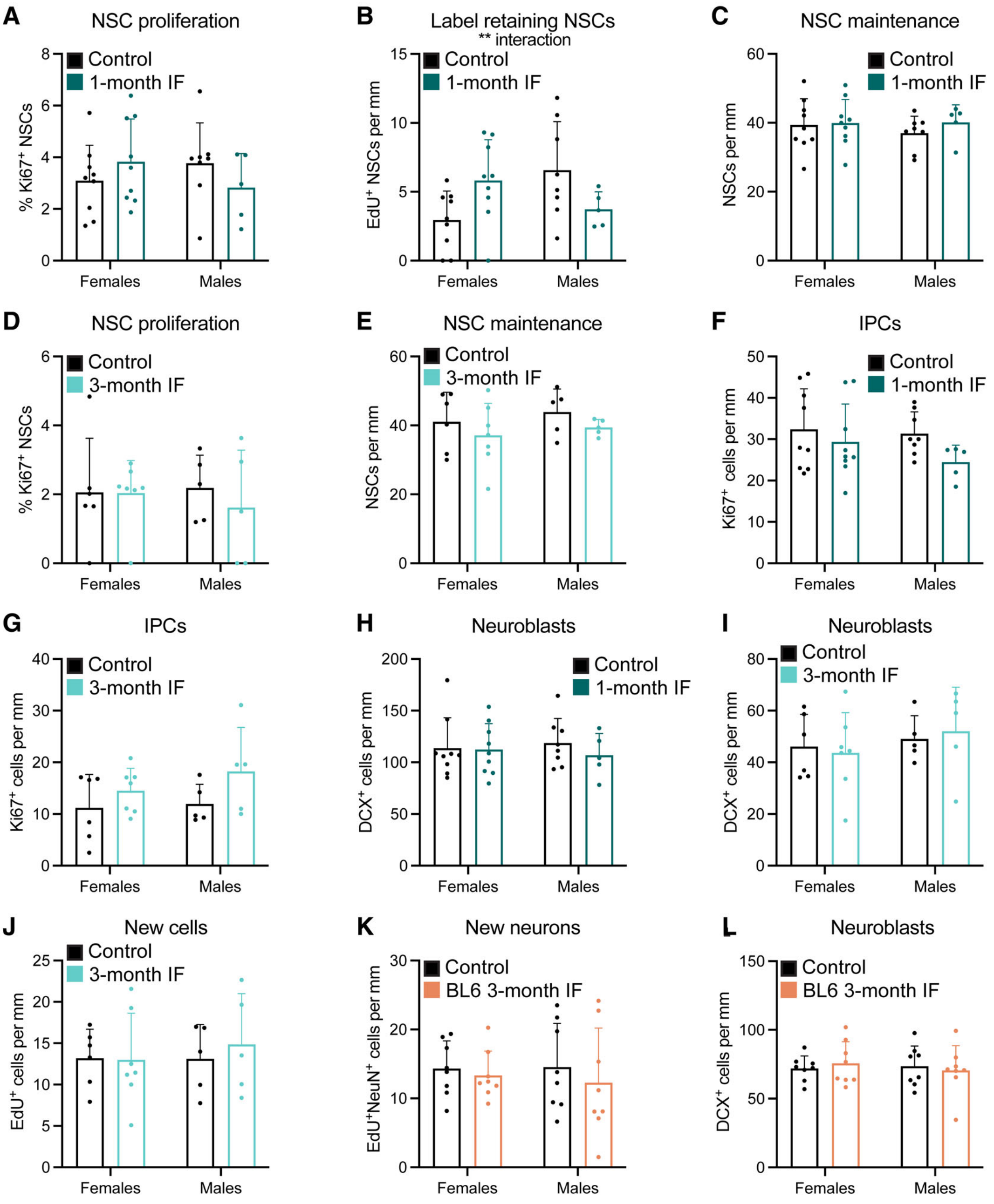

Figure EV4. Male and female mice show similar responses to IF.

A–L Data of graphs in the main figures segregated by sex showing that the response of adult neurogenesis to IF is sex-independent. The percentage of label retaining NSCs after 1 month of IF (B) shows an interaction between diet and sex that is not translated in the following steps of the neurogenic lineage.

Data information: Graphs are displayed in order of appearance on the text. Bars and error bars represent average + s.d.; dots represent individual mice. (A–C, F, H) $n_{control♀} = 9$, $n_{control♂} = 8$, $n_{IF♀} = 9$, $n_{IF♂} = 5$; (D, E, G, I, J) $n_{control♀} = 6$, $n_{control♂} = 5$, $n_{IF♀} = 7$, $n_{IF♂} = 5$; (K, L) $n = 8$ in all conditions. Two-way ANOVA (see significance values in Appendix Tables S5 and S6). Significance summary: absence of sign, $P > 0.05$; **, $P < 0.01$.

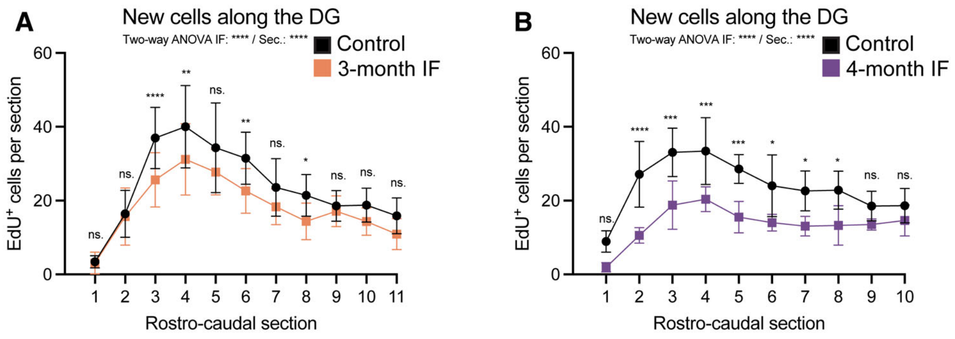

Figure EV5. Response to IF along the rostro-caudal axis of the DG.

A, B Number of EdU+ cells along the rostro-caudal axis of the DG in C57BL6/J mice after 3 or 4 months of IF. See Figs 5 and 6 for experimental design. The average of the two hemispheres in each section was used to calculate the number of cells in a 40-µm-thick DG section. The distance between sections is 240 µm. (A) $n_{control} = 16$, $n_{IF} = 16$; (B) $n_{control} = 7$, $n_{IF} = 6$. Two-way ANOVA followed by Šídák's multiple comparisons test (see significance values in Appendix Table S7).

Data information: Dots and error bars represent average ± s.d. Significance summary: ns, $P > 0.05$; *, $P < 0.05$; **, $P < 0.01$; ***, $P < 0.001$, ****, $P < 0.0001$. Sec. = rostro-caudal section.

© 2023 The Authors
