# Supplementary figures and images for: Adult neural stem cells and neurogenesis are resilient to intermittent fasting
Source: EMBO Rep. 2023 Nov 21;24(12):e57268. doi: 10.15252/embr.202357268 (PMC10702802; doi:10.15252/embr.202357268)

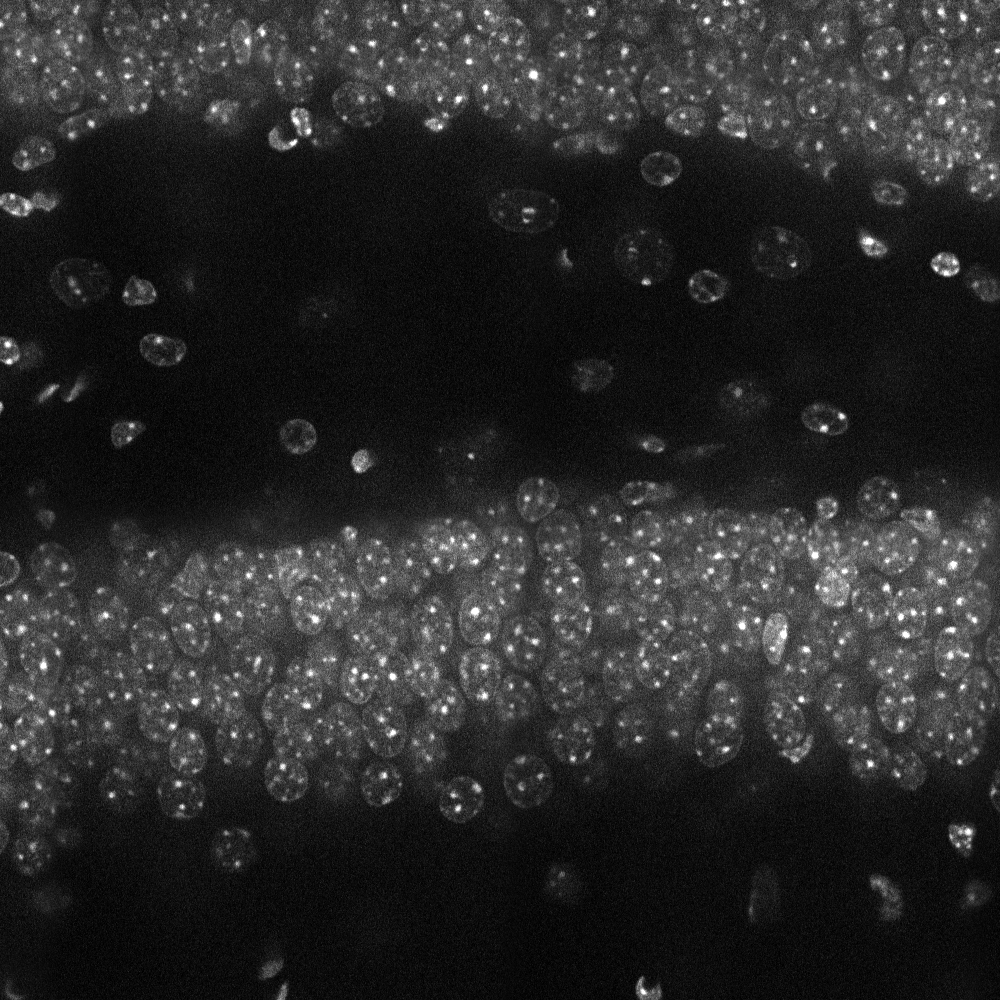

Supplement: Supplementary file 7 — Source Data for Figure 1 [file EMBR-24-e57268-s012.zip › Figure 2/2C/Figure 2C_IF.tif]

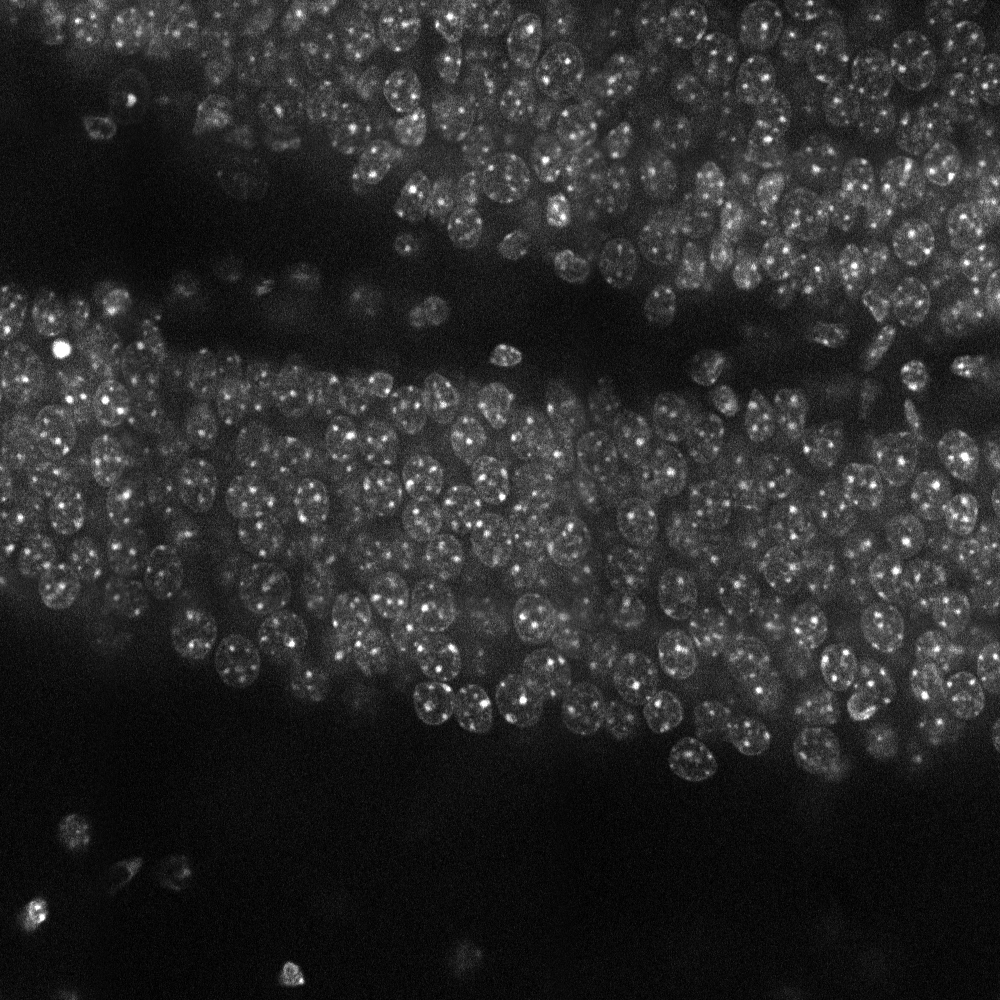

Supplement: Supplementary file 7 — Source Data for Figure 1 [file EMBR-24-e57268-s012.zip › Figure 2/2C/Figure 2C_Control.tif]
