# Supplementary material for: Adult neural stem cells and neurogenesis are resilient to intermittent fasting
Source: EMBO Rep. 2023 Nov 21;24(12):e57268. doi: 10.15252/embr.202357268 (PMC10702802; doi:10.15252/embr.202357268)
Supplement: Supplementary file 12 [file EMBR-24-e57268-s011.pdf]

EMBO reports

Adult neural stem cells and neurogenesis are resilient to intermittent fasting

Rut Gabarro-Solanas, Amarbayasgalan Davaatseren, Justus Kleifeld, Tatjana Kepcija, Thomas Koecher, Albert Giralt, Ivan Crespo-Enriquez, and Noelia Urbán
DOI: 10.15252/embr.202357268

Corresponding author(s): Noelia Urbán Avellaneda (noelia.urban@imba.oeaw.ac.at)

Review Timeline:

Transfer from Review Commons:	30th Mar 23
Editorial Decision:	11th Apr 23
Revision Received:	14th Jul 23
Editorial Decision:	30th Aug 23
Revision Received:	13th Sep 23
Accepted:	30th Oct 23

Editor: Ioannis Papaioannou / Deniz Senyilmaz Tiebe

Transaction Report: This manuscript was transferred to EMBO reports following peer review at Review Commons.

Review COMMONS

Review #1

1. Evidence, reproducibility and clarity:

Evidence, reproducibility and clarity (Required)

Summary:

In this study, mice were exposed to a specific form of so-called Intermittent Fasting (IF) and the effects of IF on adult neogenesis in the hippocampus were determined. The specific IF protocol used had no effect on activation, proliferation, or maintenance of adult Neural Stem Cells (aNSCs) and displayed a decrease in number of new neurons in the neurogenic niche but only after 1 month of the IF protocol. These results contrast previously published results from multiple studies that concluded that IF promotes survival of new neurons and by extension promote adult neurogenesis. The unresponsiveness of aNSCs or their immediate cell progeny, the Intermediate Neural Progenitors (IPCs), to IF is a novel finding. The authors make several relevant points in the discussion about the publication bias towards positive results (or omission of negative results), which may reinforce established dogmas. However, the presented results did not convincingly demonstrate that the absence of effects of IF on aNSCs or adult neurogenesis is simply not a result of a specific IF paradigm, which is not robust enough to elicit changes in adult neurogenesis. In other words, there is a lack of positive controls and alternative protocols that would rule out that the observed absence of effects is not a consequence of type II error (the error of omission), or more colloquially, a consequence of false negatives.

Major Comments:

1. Protocol-driven absence of effects: The absence of IF effects on aNSCs and IPCs observed in this study does not lend it the authority to conclude that aNSCs are resilient to IF or all IF paradigms and protocols. The absence of IF effects on aNSCs and neurogenesis could be specifically related to the chosen IF paradigm. Indeed, not all previous studies that observed IF-driven effects on adult neurogenesis used the same "night-time every-other-day fasting" protocol chosen in this study. For example, Brandhorst et al., 2015 (cited in this paper) used 4 days of IF 2x per month and observed an increase of DCX+BrdU+ cells. On the other hand, certain previous studies used the same or similar IF protocol used here, but often with longer duration or with a post-fasting ad libitum feeding period, which may be responsible for the pro-neurogenic or pro-survival effects. In fact, the authors acknowledge this in the discussion (page 7, lines 289-290 and 292-294). Why would the authors then not

include similar feeding/IF paradigm in their study and determine if these would generate effects on survival of new neurons but also on aNSCs and/or IPCs? In addition, the authors acknowledge that the chosen IF paradigm may have affected the stress levels or behaviour of mice (page 9, lines 372-378). Why did they not test if their IF protocol does not increase stress or anxiety of mice by simple behaviour tests such as open field or elevated T maze? Alarmingly, the used IF protocol does not result in changes in final weight or growth curves (S.Fig.2), which is surprising and raises a question the used IF protocol is robust enough or appropriate. Finally, the authors acknowledge that their own results do not support well-established findings such as aging-related reduction in number of aNSCs (page 4, lines 177-179). This again questions whether the selected protocols and treatments are appropriate.

2. Lack of topic-specific positive controls: The authors successfully demonstrated that the used IF protocol differentially impacts the adipose tissue and liver, while also inducing body weight fluctuations synchronized with the fasting periods. However, these peripheral effects outside the CNS do not directly imply that the chosen IF protocol is robust enough to elicit cellular or molecular changes in the hippocampus. The authors need to demonstrate that their IF protocol affects previously well-established CNS parameters associated with fasting such as astrocyte reactivity, inflammation or microglia activation, among other factors. In fact, they acknowledge this systemic problem in the discussion (page 8, lines 359-360).

3. Problematic cell analyses: Cell quantification should be performed under stereological principles. However, the presented results did not adhere to stereological quantification. Instead, the authors chose to quantify specific cell phenotypes only in subjectively selected subsets of regions of interest, i.e., the Subgranular Zone (SGZ). This subjective pre-selection may have been responsible for the absence of effects, especially if these are either relatively small or dependent on anatomical sections of SGZ. For example, IF may exert effects on caudal SGZ more than on rostral SGZ. But if the authors quantified only (or predominantly) rostral SGZ, they may have missed these effects by biasing one segment of SGZ versus other. The authors should apply stereological quantification at least to the quantification of new neurons and test if this approach replicated previously observed pro-survival effects of IF. Also, the authors should describe how they pre-selected the ROI for cell quantification in greater details.

4. Alarming exclusion of data points: There appears to be different number of data points in different graphs that are constructed from same data sets. For example, in the 3-month IF data set in Figure 4, there are 14 data points for the graph of Ki67+ cells (Fig.4B), but 16 (or 17) data points for the graph of DCX+ cells (Fig.4D). How is that possible? If data points were excluded, what objective and statistical criteria were applied to make sure that such exclusion is not subjective and biased? In fact, the authors state that "Samples with poor staining quality were also excluded from quantifications" (page 12, line 528-529). Poor preparation of tissue is not only

suboptimal but not a valid objective reason for data point exclusion. This major issue needs to be explained and corrected.
5. Different pulse-and-chase time-points: One of the reasons why this study has found that aNSCs may not be responsive to IF could be the use of less appropriate pulse-and-chase time-points either after EdU or after Tamoxifen for cell lineage tracing. The authors observed that IF has negative effects on new neurons initially (Fig.4F). Similarly, it is well established that voluntary physical exercise affects SGZ adult neurogenesis only during the first 2 weeks. After this period, the neurogenic effects of exercise are diminished beyond observational detection (i.e., van Praag's and Kempermann's papers in the past 25 years). These two arguments suggest that the observed absence of aNSC responsiveness might be a consequence of the chosen EdU administration and the EdU pulse should not be administered 15 days after Tamoxifen/IF protocol start but earlier, in the first week of the IF protocol. In fact, the decreased number of new neurons during the initial IF phase may not be only a consequence of reduced survival but of higher aNSC quiescence during the first week of the IF protocol.
6. Discussion needs more specificity and clarity: The authors claim that the absence of IF effects on neurogenesis is multi-layered (including the influence of age, sex, specific cell labelling protocols etc.) but they do not specifically address why certain studies did find IF-driven neurogenic effects while they did not. In addition, some statements and points in the discussion are not clear. For example, when the authors refer to their own experiments (page 8, lines 331-334), it is not clear, which experiments they have in mind.

Minor comments:

1. Change in the title: The authors have shown that a very specific IF protocol does not affect aNSCs but initially decreases number of new neurons in SGZ. The title should reflect this. For example, it could state "Specific (night-time every-other-day) fasting does not affect aNSCs but initially decreases survival of new neurons in the SGZ".
2. Data depiction: Data in 3 datasets were found not normally distributed (Fig. S5A, B and S6A) and were correctly analysed with non-parametric tests. However, the corresponding graphs wrongly depict the data as mean +/- SD while they should depict median +/- IQR (or similar adequate value) because non-parametric statistical tests do not compare means but medians.
3. Statistical analysis: For ANOVA, the F and p values are not listed anywhere. The presented asterisks in the graphs are only for non-ANOVA or ANOVA post-hoc tests. This does not allow to judge statistical significance well and should be corrected.
4. Asymmetric vs Symmetric cell divisions: Representative images in Fig.2B suggest that IF may affect the plane of cell division for the Type-1 aNSCs. The plane of cell

division is an indirect indicator of symmetric vs asymmetric (exhaustive vs maintaining) modes of cell division. Is it possible, IF influences this, especially during the first week of IF (see major comment 5)?
5. Improved and more accurate citations: Some references are not properly formatted (e.g., "Dias", page 7, line 288). Some references are included in generalizing statements when they do not contain data to support such statements. For example, Kitamura et al., 2006 did not determine the number of new neurons (only BrdU+ cells) in the SGZ, yet this reference is included among sources supporting that IF "promote survival of newly born neurons" (page 2, line 60). Authors should be more careful how the cite the references.
6. How do the authors explain that they observe 73-80% caloric restriction and yet the final body weight is not different between IF and control animals? Would it suggest that the selected IF protocol or selected diet are not appropriate (see major point 4)?
7. Given that aNSCs rely more on de novo lipogenesis and fatty acids for their metabolism as shown by Knobloch et al., Nature 2013 and given the interesting changes in RER with the IF shown in this study, it would be interesting to see whether there are differences in Fasn expression in aNSCs between control and IF animals (see minor point 4).
8. Determining apoptosis in the SGZ by picnotic nuclei (Figure S6A) should be supplemented by determining the number and/or proportion of YFP+ cells positive for the Activated Caspase 3.

2. Significance:

Significance (Required)

General assessment:

This study concludes that aNSCs do not respond to the intermittent fasting. This expands and supplements previous findings that suggest that the intermittent fasting promotes adult neurogenesis by increasing survival and/or proliferation in the Subgranural Zone. The study is well designed, however, over-extends its conclusions beyond a specific fasting paradigm and does not acknowledge serious limitations in the experimental design and analyses. In fact, until major revision is done, which would rule out that the absence of effects of fasting on aNSCs is not due to false negative results, many conclusions from this study cannot be accepted as valid.

Advance:

As mentioned above, the study has a potential to advance our understanding of how fasting affects neurogenesis and fills the knowledge gap of how fasting specifically

affects the stem cells. However, unless the study addresses its limitations, its conclusions are not convincing.

Audience:

This study would be particularly interesting for the niche readers from the neurogenesis field. However, the study can also be interesting for researchers in metabolomics and dietology.

My expertise:

adult neurogenesis, neural stem cells, dietology, metabolism

3. How much time do you estimate the authors will need to complete the suggested revisions:

Estimated time to Complete Revisions (Required)

(Decision Recommendation)

More than 6 months

4. *Review Commons* values the work of reviewers and encourages them to get credit for their work. Select 'Yes' below to register your reviewing activity at Web of Science Reviewer Recognition Service (formerly Publons); note that the content of your review will not be visible on Web of Science.

Web of Science Reviewer Recognition

Yes

Review #2

1. Evidence, reproducibility and clarity:

Evidence, reproducibility and clarity (Required)

In this manuscript, Gabarro-Solanas et al. question the suitability of IF (Intermittent fasting - non-pharmacological strategy to counteract ageing, which has been previously shown to increase the number of adult-born neurons in the dentate gyrus of mice) as a pro-neurogenic intervention, since IF treatment did not stimulate adult hippocampal neurogenesis, neither at the stem cell level nor on immature and/or dividing neurons. The Authors used a tamoxifen inducible transgenic model (Glast-CreERT2;RYFP mice) to trace neural stem cell lineage and found that IF did not enhance neural stem cell proliferation, nor the abundance of immature, DCX+ neurons. Three-months of IF failed to increase the number of new adult-born neurons (NeuN+/YFP+), while one month of IF significantly reduced the number of new adult-born neurons.

The study appers technically sound, including many different approaches in order to reach its conclusions.
For instance, tamoxifen has been reported to impair various physiological processes, including neurogenesis (Smith et al., 2022), and most studies on adult hippocampal neurogenesis use the C57BL/6J strain of mice; hence, the use of Tamoxifen or that of the GlastCreERT2;RYFP model may have underscored these observations. However, to account for this potentially confounding factor, the Authors characterised the effect of their IF treatment in C57BL/6j mice, also reporting no evident effects of IF as a pro-neurogenic intervention.
I think the study was carefully planned and the analyses well done. Several possible variables were considered, including sex, labelling method, strain, tamoxifen usage or diet length. Several controls were performed in other organs and tissues (liver, fat) to establish the fasting protocol and to check its effects.
Data are presented in a clear way. Quality of images is high level.
In general, it appears as a highly reliable paper reaching an authoritative conclusion for the absence of effect of IF on adult neurogenesis.

Major comments:

I think that the key conclusions are convincing and no further experiments are required.
The methods are presented in such a way that they can be reproduced, and the

experiments adequately replicated with proper statistical analysis.

Minor comments:

Prior studies are referenced appropriately, both regarding the IF protocols and the adult neurogenesis modulation.
Line 288 - a reference is incomplete (Dias); integrate with: (Dias et al., 2021)
There is one concept that is not expressed in the manuscript. Maybe it is not strictly necessary, but I think can be useful to mention it here. It is the fact that most information currently available strongly indicates that adult neurogenesis in humans is not present after adolescence. Of course the research described here is carried out on mice, and in the manuscript it is stated many times that adult hippocampal neurogenesis is strongly decreasing with age, also due to age-related stem cell depletion. Yet, it seems that in humans the exhaustion of such a process can start after adolescence. We know that a sort of controversy is currently present on this subjects, because DCX+ neurons can be detected in adult and old human hippocampi. Yet, it is also clear that there is no substantial cell division (stem cells are depleted) to sustain such hypothetical neurogenesis. Hence, it has been hypothesized that non-newlyborn, "immature" neurons can persist in the absence of cell division, as it has been well demonstrated in the cerebral cortex (see La Rosa et al., 2020 Front Neurosci; Rotheneichner et al., 2018, Cereb Cortex).
This point can be important in the case someone want to use dietary approached such as IF (or any other pharmacological treatment) to stimulate neurogenesis in humans.

2. Significance:

Significance (Required)

The significance of this study relies on the fact that adult neurogenesis field (AN) has been often damaged by the search of "positive" results, aiming at showing that AN does occur "always and everywhere" and that most internal/external stimuli do increase it. This attitude created a bias in the field, persuading many scientists that a result in AN is worthy of publication (or of high impact factor publication) only when a positive result is found.

Personally, I found particularly meaninful the last sentences of the Discussion (reported below), which might seem "off topic" in a research paper, while - I think - underline the real significance of the manuscript:
"In addition, publication bias might be playing a role in skewing the literature on fasting and neurogenesis towards reporting positive results.

In some reviews, even studies reporting no effect are cited as evidence for improved neurogenesis upon IF. Reporting of negative results, especially those challenging accepted dogmas, and a careful and rigorous evaluation of the publications cited in reviews are crucial to avoid unnecessary waste of resources and to promote the advancement of science."

Reviewer field of expertise - keywords: adult neurogenesis, brain structural plasticity, non-newly born immature neurons, comparative neuroplasticity.

3. How much time do you estimate the authors will need to complete the suggested revisions:

Estimated time to Complete Revisions (Required)

(Decision Recommendation)

Less than 1 month

4. *Review Commons* values the work of reviewers and encourages them to get credit for their work. Select 'Yes' below to register your reviewing activity at Web of Science Reviewer Recognition Service (formerly Publons); note that the content of your review will not be visible on Web of Science.

Web of Science Reviewer Recognition

No

Review #3

1. Evidence, reproducibility and clarity:

Evidence, reproducibility and clarity (Required)

In this manuscript, Gabarro-Solanas et al. investigate the effects of intermittent fasting (IF) on adult hippocampal neurogenesis in young adult mice. IF has been reported to increase the number of adult-born neuron in the hippocampus, a region that is important for learning and memory. However, it is not well understood what stages of adult neurogenesis are regulated by IF. To address this, the authors utilized lineage tracing and label retention assays in mice undergoing an IF diet. The authors used 2 months old Glast-CreERT2;RYFP mice in combination with Edu label retention to characterize adult NSCs and placed these mice on 1 and 3 months of IF. Despite seeing a decrease in neural stem cell proliferation with age, the authors did not observe a change due to diet. The authors then used immunohistochemistry to characterize changes in cell proliferation, neuroblasts, and new neurons following 1 month and 3 months of IF. Only 1 month of IF seemed to decrease the number of new neurons; however, by 3 months the neuronal output was the same. There were no differences in neuroblasts or cell proliferation due to diet. Gabarro-Solanas et al. conclude that IF transiently and mildly inhibits neurogenesis. Due to contradicting results, the authors then try to determine what variables (sex, labeling method, strain, tamoxifen usage, or diet length) could be affecting their data. The authors saw no substantial differences due to any of their variables.

Major Points

1. The authors analyze NSCs homeostasis and neurogenesis in young adult mice and do not observe any significant changes with their chosen alternate day intermittent fasting paradigm. However, a lot of the data and cell counts appears to be highly variable between animals in the same group. At times, there is an order of magnitude difference between the highest and lowest counts (e.g. Figure 2C,E). According to the method section, it appears that the authors predominantly analyzed a single DG (section?) for most immunostainings, which may explain the large variability in their data. If this is indeed the case, it is insufficient to quantify only a single section for each animal. The authors should quantify several DG sections for each mouse from a pre-defined range along the rostral-caudal axis of the hippocampus in accordance with a standard brain reference atlas. There are also several quantifications, especially of Ki67 where several individuals appear to have no Ki67+ (Figure 3B, 6D) NSCs. These findings are surprising given the still young age of these mice and may be

another reflection of the limited brain sections that were analyzed.
2. There appear to be significant cutting or imaging artifacts across most fluorescent images further raising concerns regarding the accuracy of the quantifications (e.g. Figure 3D, 4C,E, 6B) and publication quality of the images and data. Importantly, uneven section thickness, either from cutting artifacts or imaging issues, may lead to inaccurate cell quantifications a could, possibly, account for the high variability. This issue would further exacerbate concerns regarding the quantification of a single DG section for each animal.
3. It is unclear how NSCs were counted in the B6 mice (Fig 6D,E). The authors only provide a description for the Glast-CRE mice, where they used YFP labeling and GFAP. We assume they performed Sox2/GFAP or Nestin labeling, however, this is not clear at all. The authors should describe their methodology and provide representative images.
4. NSC populations represent a heterogenous group of stem cells with different replicative properties. As such, the Glast-Cre approach used for the majority of this study may represent a specific subset of NSCs. In line with the previous point, we recommend the authors complement their NSC counts with Sox2/GFAP and Nestin immunostainings.
5. Stress is a significant negative regulator of neurogenesis. Is it possible that the IF mice display higher stress level which could counteract any beneficial effects of the IF intervention. The authors should provide some measures of stress markers to rule out this potential confounding factor in their IF paradigm.

Minor Point

1. The authors state that "Experimental groups were formed by randomly assigning mice from different litters within each mouse strain and all experiments were conducted in male and female mice". Given that neurogenesis, especially at young ages, is highly sensitive to the exact age of the mice, the authors should provide a rationale why animals from different litters instead of littermate controls were used in these experiments.
2. Currently, the statistical tests are only described in the method section, however it would be helpful if this information to be integrated into the figure legend as well. Additionally, the authors provide individual data points for some but not all bar graphs (eg Figure 1D).
3. Cell counts per AU is a rather unorthodox unit. With a representative selection of tissue for each animal, the authors could avoid the need to normalize to the DG length and may be able to extrapolate an estimate of cell counts for the entire DG instead.
4. In Figure 4D, the authors highlight a few NSC with arrowheads. At a quick glance this is rather confusing as it appears that the authors only counted 3 NSCs in each picture. It may be a better option to show a zoomed in picture to highlight an example

of a representative NSC.
5. In Supplementary Figure S6, the authors should complement the quantification of the nuclei with representative images.
6. For the daytime IF, did the authors assess weights, food intake, RER as well liver/fat measurements similar to night-time IF? If so, this data should be provided in the supplement.

2. Significance:

Significance (Required)

The authors are commended for compiling a manuscript on what is commonly considered 'negative data', that, at the same time, are also contradicting independent reports on the effects of IF on neurogenesis. The studies outlined in this manuscript are comprehensive and mostly well designed. Given the broad, growing interest in dietary restriction as an aging intervention the study is timely.

3. How much time do you estimate the authors will need to complete the suggested revisions:

Estimated time to Complete Revisions (Required)

(Decision Recommendation)

Between 1 and 3 months

4. *Review Commons* values the work of reviewers and encourages them to get credit for their work. Select 'Yes' below to register your reviewing activity at Web of Science Reviewer Recognition Service (formerly Publons); note that the content of your review will not be visible on Web of Science.

Web of Science Reviewer Recognition

Yes

Review #4

1. Evidence, reproducibility and clarity:

Evidence, reproducibility and clarity (Required)

Summary:

In this manuscript, Gabarró-Solanas et al. tested the effect of intermitted fasting (IF, every-other-day fasting) on adult neural stem cells and neurogenesis. They demonstrate that the paradigm they have used does not affect NSC activation or maintenance, and does also not promote neurogenesis. As previous reports showed increased neurogenesis with IF, the authors controlled for various parameters such as mouse strain, sex, and diet length. They also used different methods of identification of newborn neurons, such as tamoxifen-induced lineage-tracing versus birth-dating with thymidine-analogues to substantiate their findings.

Major comments:

This study is very well done with carefully designed and controlled experiments. The manuscript reads nicely and the data are presented in a clear way, making it easy to follow. The authors have done a "tour-de force" to rule out confounding factors that might influence their findings that IF does not affect NSCs nor neurogenesis.
The claims and conclusions are supported by the data. The methods are clearly described and should allow to reproduce the data independently. The number of replicates (i.e. the number of mice analyzed) is impressive and statistical analysis is adequate.

The major findings, namely that the chosen IF does not affect NSCs and neurogenesis is not in line with some previous studies. Despite a careful ruling out of potentially confounding factors (see also "significance" below), it remains unclear why other studies have found an increase in neurogenesis with IF. As each of these studies has some specific experimental design, it is difficult to judge these data in the context of previous data without going through all the details of the other studies. It would thus be a great help for the reader if the authors could provide a table or schematic, which lists the major parameters of each of these studies, such as detailed paradigm of IF, age of mice at start, sex, duration of the intervention, method of identification of NSCs and neurogenesis etc.

Two points that the authors have not discussed might also be worth mentioning in the

discussion part:

1. The mice in the night-time IF were single caged, could there be a potential negative effect on neurogenesis that would mask the presumably beneficial effect of IF? Although the controls were also single caged, the stress of social isolation might play a role?
2. The IF mice gained the same weight over time (Fig. S2), but had a ~20% reduction in overall calory intake. This would be explainable by a reduction in energy expenditure, but the overall activity was also not significantly changed (Fig. S1). Can the authors speculate why they reach the same weight with less calories?

Minor comments:

1. It would be nice to replace the arbitrary units (AU) in the graphs were this is used (e.g. Fig. 2F, 3C, 4B, D and F etc) to the actual number of cells per a certain µm DG, so that the number of cells can be put in context and compared between the figures.
2. Fig 3 D: can the authors also show the Ki67 channel to illustrate how it looks after a 3 month IF?
3. Fig.4E: the NeuN staining looks strangely interrupted, this might be due to tile-stitching? In that case, it would be better to either only show one segment or to try to get a better stitching algorhythm.
4. Fig.6 D shows a minus axis in Y-axis, this should only been shown from 0 to positive values, as it is a percentage of cells and cannot be negative.
5. Fig.6 B: the same problem with the NeuN staining as mentioned under point 3. This should be improved.
6. Fig. S6B: maybe add a comment in the result part or in the figure legend that a 10 day chase after an EdU pulse is not the classical protocol to look at mature NeuN positive neurons. But apparently enough newborn neurons were already NeuN positive for this quantification.
7. The authors refer to personal communications with M. Mattson and S. Thuret to underline that circadian disruption is not enough to explain the differences (line 367 onwards). Can they refer the reader to published data instead?

2. Significance:

Significance (Required)

Given the great interest in the seemingly positive effects on health of IF in general, and also for increasing neurogenesis, it is important to better understand the mechanism of this intervention. The study by Gabarró-Solanas et al. clearly demonstrates that IF is not a universal, "works all the time" way of increasing

neurogenesis. The study is very well done, with well controlled and measured parameters. It shows that a physiological interference such as IF might depend on many factors and might be less robust across laboratories than anticipated. This study is a very good example that all the details of the experimental settings need to be taken into consideration and are ideally reported with every IF study. It is also a good example how to follow up "no effect" data in a way that they are conclusive.

The significance of this study is to point out that IF as a strategy to increase neurogenesis needs to be reconsidered. It raises the questions how IF can be beneficial in some studies and not in others, asking for more experiments to better understand the detailed mechanisms of IF action. In a systematic approach, this study rules out some of the potentially confounding factors and shows that at least with the chosen IF paradigm, these factors are not the reason for not seeing increased neurogenesis. The study is thus of clear interest for the neurogenesis field and will also need to be considered by the broader field of IF research, although it speaks against the beneficial effects of IF. It might have the potential to bring together the different study authors who did or did not see increased neurogenesis with IF and discuss together the non-published details of their study design to advance the field.

3. How much time do you estimate the authors will need to complete the suggested revisions:

Estimated time to Complete Revisions (Required)

(Decision Recommendation)

Less than 1 month

4. *Review Commons* values the work of reviewers and encourages them to get credit for their work. Select 'Yes' below to register your reviewing activity at Web of Science Reviewer Recognition Service (formerly Publons); note that the content of your review will not be visible on Web of Science.

Web of Science Reviewer Recognition

No

Revision Plan

Manuscript number: RC-221212
Corresponding author(s): Noelia, Urbán

1. General Statements [optional]

This section is optional. Insert here any general statements you wish to make about the goal of the study or about the reviews.

2. Description of the planned revisions

We provide here a point-by-point response to the reviewer's comments, with our answers and comments in blue.

Reviewer #1 (Evidence, reproducibility and clarity (Required)):
Summary:
In this study, mice were exposed to a specific form of so-called Intermittent Fasting (IF) and the effects of IF on adult neogenesis in the hippocampus were determined. The specific IF protocol used had no effect on activation, proliferation, or maintenance of adult Neural Stem Cells (aNSCs) and displayed a decrease in number of new neurons in the neurogenic niche but only after 1 month of the IF protocol. These results contrast previously published results from multiple studies that concluded that IF promotes survival of new neurons and by extension promote adult neurogenesis. The unresponsiveness of aNSCs or their immediate cell progeny, the Intermediate Neural Progenitors (IPCs), to IF is a novel finding. The authors make several relevant points in the discussion about the publication bias towards positive results (or omission of negative results), which may reinforce established dogmas. However, the presented results did not convincingly demonstrate that the absence of effects of IF on aNSCs or adult neurogenesis is simply not a result of a specific IF paradigm, which is not robust enough to elicit changes in adult neurogenesis. In other words, there is a lack of positive controls and alternative protocols that would rule out that the observed absence of effects is not a consequence of type II error (the error of omission), or more colloquially, a consequence of false negatives.
We thank the reviewer for acknowledging the importance and novelty of our findings. On them being the result of a specific IF paradigm, we must point out that we used the same IF paradigm as in previous studies that had shown changes in neurogenesis upon IF. We do not claim that IF is unable to increase neurogenesis in all conditions, but report that IF is not a reliable method to increase adult neurogenesis (in particular, every-other-day intermittent fasting with food re-administration in the evening). We have repeated the experiment multiple times in different strains, always with enough animals to make our experiments conclusive and we never observed an increase in adult neurogenesis, effectively ruling out that our results are a false negative. Of note, even if other protocols might indeed increase neurogenesis (which we never claimed cannot) that would not make our results a false negative.

Major Comments:

1. Protocol-driven absence of effects: The absence of IF effects on aNSCs and IPCs observed in this study does not lend it the authority to conclude that aNSCs are resilient to IF or all IF paradigms and protocols. The absence of IF effects on aNSCs and neurogenesis could be specifically related to the chosen IF paradigm. Indeed, not all previous studies that observed IF-driven effects on adult neurogenesis used the same "night-time every-other-day fasting" protocol chosen in this study. For example, Brandhorst et al., 2015 (cited in this paper) used 4 days of IF 2x per month and observed an increase of DCX+BrdU+ cells. On the other hand, certain previous studies used the same or similar IF protocol used here, but often with longer duration or with a post-fasting ad libitum feeding period, which may be responsible for the pro-neurogenic or pro-survival effects. In fact, the authors acknowledge this in the discussion (page 7, lines 289-290 and 292-294). Why would the authors then not include similar feeding/IF paradigm in their study and determine if these would generate effects on survival of new neurons but also on aNSCs and/or IPCs?
As just stated above, we never claimed that aNSCs are resilient to all IF paradigms. We refer to fasting in general in the introduction but quickly focus on every-other-day fasting throughout the paper and directly compare our results only to similar IF paradigms. We chose the most commonly used IF paradigm that had been shown to increase adult neurogenesis. As the reviewer points out, we speculate in the discussion that a refeeding period may explain the differences between our results and others. This is because a post-fasting ad libitum period was introduced in the study published in Dias et al. 2021. We are currently analysing a new experiment in which we replicate the IF protocol in that study, which we will include in our revised version.

In addition, the authors acknowledge that the chosen IF paradigm may have affected the stress levels or behaviour of mice (page 9, lines 372-378). Why did they not test if their IF protocol does not increase stress or anxiety of mice by simple behaviour tests such as open field or elevated T maze?
While testing all possible causes for the lack of positive results in our experiments is not viable, we do agree with the reviewer that stress levels might indeed influence the outcome of the experiments. We will collect blood from ad libitum-fed and fasted mice to analyse the levels of stress hormones (e.g. corticosterone). The results will be included in our revised version. These measurements will give us a more accurate reading of stress levels than behavioural tests. Of note, regardless of the outcome of this experiment, our conclusions will remain identical. We will not be able to compare stress levels with previous publications, as they were not tested. And if the protocol did increase stress levels, it would still argue that IF is not a reliable method to increase neurogenesis (as presumably might or might not increase stress to levels that affect neurogenesis).

Alarmingly, the used IF protocol does not result in changes in final weight or growth curves (S.Fig.2), which is surprising and raises a question the used IF protocol is robust enough or appropriate.
We were also surprised by the lack of change in the final weight our IF mice respect to control. Differences in final weight between different labs despite using the exact same protocol are one of the reasons why we conclude that this IF paradigm is not a robust intervention. However, we

are not the first ones to report little or no difference in weight upon IF in C57BL6/J mice (Goodrick et al., 1990 and Anson et al., 2003) and this would not be a reason to dismiss the experiment since the benefits in crucial circulating factors induced by IF seem to be independent of weight loss (Anson et al., 2003).

Finally, the authors acknowledge that their own results do not support well-established findings such as aging-related reduction in number of aNSCs (page 4, lines 177-179). This again questions whether the selected protocols and treatments are appropriate.
As we already discuss, we believe this might be due to a difference between strains in the time when aNSC numbers decline. Nevertheless, we will complement our current data by counting the number of aNSCs at 1 and 3 months post-tamoxifen (3 and 5 month old mice) using GFAP, Sox2 and Nestin triple stainings (as suggested by another reviewer).

2. Lack of topic-specific positive controls: The authors successfully demonstrated that the used IF protocol differentially impacts the adipose tissue and liver, while also inducing body weight fluctuations synchronized with the fasting periods. However, these peripheral effects outside the CNS do not directly imply that the chosen IF protocol is robust enough to elicit cellular or molecular changes in the hippocampus. The authors need to demonstrate that their IF protocol affects previously well-established CNS parameters associated with fasting such as astrocyte reactivity, inflammation or microglia activation, among other factors. In fact, they acknowledge this systemic problem in the discussion (page 8, lines 359-360).
We fully agree with the reviewer in that even though the chosen IF protocol induces peripheral effects, it is not robust enough to elicit cellular or molecular changes in the hippocampus, and this is precisely the message of our paper. We have looked for references showing the influence of IF on astrocyte reactivity or microglia activation, but the studies we found so far look at the effects of IF and other forms of fasting in the CNS in combination with pathologies such as Alzheimer's disease, Multiple Sclerosis, physical insults or aging (Anson et al., 2003; Chignarella et al., 2018; Rangan et al., 2022; Dai et al., 2022. Reviewed in Bok et al., 2019 and Gudden et al., 2021). Fasting seems to reduce astrocyte reactivity, inflammation or microglia activation in these pathological situations respect to the same pathology in ad libitum mice, but its effect in control, healthy mice is far less clear. In fact, the only reference that we could find where healthy mice were included in the analysis showed that these benefits only happened in the context of the injury (Song et al., 2022).

3. Problematic cell analyses: Cell quantification should be performed under stereological principles. However, the presented results did not adhere to stereological quantification. Instead, the authors chose to quantify specific cell phenotypes only in subjectively selected subsets of regions of interest, i.e., the Subgranular Zone (SGZ). This subjective pre-selection may have been responsible for the absence of effects, especially if these are either relatively small or dependent on anatomical sections of SGZ. For example, IF may exert effects on caudal SGZ more than on rostral SGZ. But if the authors quantified only (or predominantly) rostral SGZ, they may have missed these effects by biasing one segment of SGZ versus other. The authors should apply stereological quantification at least to the quantification of new neurons and test if this approach

replicated previously observed pro-survival effects of IF. Also, the authors should describe how they pre-selected the ROI for cell quantification in greater details.

We did analyse only the more septal region of the hippocampus, which we will make clear in the text. As also suggested by other reviewers, we will include stereological counts of the neuronal output of aNSCs in the revised version. As for selecting the SGZ for aNSC counts, this is the standard in the field, as one of the criteria to identify aNSCs is precisely the location of their nucleus in the SGZ. Neuroblasts and new neurons were counted both in the SGZ and the granule cell layer. There was no subjective pre-selection of areas of interest since we counted the whole DG in each section and not a specific random region.

4. Alarming exclusion of data points: There appears to be different number of data points in different graphs that are constructed from same data sets. For example, in the 3-month IF data set in Figure 4, there are 14 data points for the graph of Ki67+ cells (Fig.4B), but 16 (or 17) data points for the graph of DCX+ cells (Fig.4D). How is that possible? If data points were excluded, what objective and statistical criteria were applied to make sure that such exclusion is not subjective and biased? In fact, the authors state that "Samples with poor staining quality were also excluded from quantifications" (page 12, line 528-529). Poor preparation of tissue is not only suboptimal but not a valid objective reason for data point exclusion. This major issue needs to be explained and corrected.

As we disclose in the methods, those stainings that did not work were excluded. This was done always before counting. Different samples were used in different counts because of the variability of staining quality between different antibodies. We will look back into the samples that failed in at least one of the stainings and exclude them from all counts, so that only samples for which all stainings worked are considered. These revised graphs will be provided in our revised version of the manuscript.

5. Different pulse-and-chase time-points: One of the reasons why this study has found that aNSCs may not be responsive to IF could be the use of less appropriate pulse-and-chase time-points either after EdU or after Tamoxifen for cell lineage tracing. The authors observed that IF has negative effects on new neurons initially (Fig.4F). Similarly, it is well established that voluntary physical exercise affects SGZ adult neurogenesis only during the first 2 weeks. After this period, the neurogenic effects of exercise are diminished beyond observational detection (i.e., van Praag's and Kempermann's papers in the past 25 years). These two arguments suggest that the observed absence of aNSC responsiveness might be a consequence of the chosen EdU administration and the EdU pulse should not be administered 15 days after Tamoxifen/IF protocol start but earlier, in the first week of the IF protocol. In fact, the decreased number of new neurons during the initial IF phase may not be only a consequence of reduced survival but of higher aNSC quiescence during the first week of the IF protocol.

We fully agree with the reviewer that BrdU or EdU pulses can give a biased view of the effects of any intervention on neurogenesis and that the EdU and Tamoxifen protocols would not allow us to detect an increase in neurogenesis during the first few days of IF. We cannot rule out that IF has a transient effect on aNSCs at some point of the treatment, but this hypothetical effect does not seem to have any consequences on neuronal output or aNSC maintenance. As for the effects

on neurogenesis in the longer IF treatments, we used the same EdU protocol as in previous publications: administration after 2/3 months of IF and analysis after one month of chase.

6. Discussion needs more specificity and clarity: The authors claim that the absence of IF effects on neurogenesis is multi-layered (including the influence of age, sex, specific cell labelling protocols etc.) but they do not specifically address why certain studies did find IF-driven neurogenic effects while they did not. In addition, some statements and points in the discussion are not clear. For example, when the authors refer to their own experiments (page 8, lines 331-334), it is not clear, which experiments they have in mind.
We will double check our discussion and improve its clarity and direct comparison to other studies.

Minor comments:
1. Change in the title: The authors have shown that a very specific IF protocol does not affect aNSCs but initially decreases number of new neurons in SGZ. The title should reflect this. For example, it could state "Specific (night-time every-other-day) fasting does not affect aNSCs but initially decreases survival of new neurons in the SGZ".
We find our title, together with the abstract, clearly and faithfully represent our findings and would rather prefer to keep our current title unmodified.

2. Data depiction: Data in 3 datasets were found not normally distributed (Fig. S5A, B and S6A) and were correctly analysed with non-parametric tests. However, the corresponding graphs wrongly depict the data as mean +/- SD while they should depict median +/- IQR (or similar adequate value) because non-parametric statistical tests do not compare means but medians.
We thank the reviewer for spotting this, we will correct the graphs in Fig. S5A, B and S6A.

3. Statistical analysis: For ANOVA, the F and p values are not listed anywhere. The presented asterisks in the graphs are only for non-ANOVA or ANOVA post-hoc tests. This does not allow to judge statistical significance well and should be corrected.
Again, thanks for spotting this, we will include them.

4. Asymmetric vs Symmetric cell divisions: Representative images in Fig.2B suggest that IF may affect the plane of cell division for the Type-1 aNSCs. The plane of cell division is an indirect indicator of symmetric vs asymmetric (exhaustive vs maintaining) modes of cell division. Is it possible, IF influences this, especially during the first week of IF (see major comment 5)?
This is an interesting hypothesis. However, since we do not see any effects on aNSC maintenance, it is unlikely that IF produces any long-lasting effects on the mode of division of aNSCs. In general, we did not notice a difference in the plane of division of aNSCs between control and IF mice, although we did not systematically test for this (would require specific short EdU pulses to capture aNSCs in M-phase). In Figure 2B, the two stem cells shown in the control are unlikely to be the two daughter cells after the division of one aNSC, as one of them is positive and the other negative for Ki67. We only pointed to the second one to show a Ki67-negative aNSC. We will emphasize this in the figure legend.

5. Improved and more accurate citations: Some references are not properly formatted (e.g., "Dias", page 7, line 288). Some references are included in generalizing statements when they do not contain data to support such statements. For example, Kitamura et al., 2006 did not determine the number of new neurons (only BrdU+ cells) in the SGZ, yet this reference is included among sources supporting that IF "promote survival of newly born neurons" (page 2, line 60). Authors should be more careful how the cite the references.
Thanks for spotting these mistakes, we will correct them and check again all our references. As for the sentence where the Kitamura paper is cited, most of the other references also use only BrdU+ cells while concluding that IF enhances the survival of new neurons. We will change new neurons for new cells to reflect this, which we already bring up in the discussion (see also extended discussion in previous BioRxiv version).

6. How do the authors explain that they observe 73-80% caloric restriction and yet the final body weight is not different between IF and control animals? Would it suggest that the selected IF protocol or selected diet are not appropriate (see major point 4)?
We also found this surprising and were expecting a change in overall activity in IF mice, which we did not observe. Many factors might play a role, like, as the reviewer suggests, changes in stress levels, which we will measure and show in the revised version.

7. Given that aNSCs rely more on de novo lipogenesis and fatty acids for their metabolism as shown by Knobloch et al., Nature 2013 and given the interesting changes in RER with the IF shown in this study, it would be interesting to see whether there are differences in Fasn expression in aNSCs between control and IF animals (see minor point 4).
This is an interesting suggestion but given that we see no effect on aNSCs, we find it’s unlikely and unnecessary to test for Fasn expression differences in our IF protocol.

8. Determining apoptosis in the SGZ by picnotic nuclei (Figure S6A) should be supplemented by determining the number and/or proportion of YFP+ cells positive for the Activated Caspase 3.
We previously found that counting picnotic nuclei is a more accurate and sensitive readout of cell death in the DG, as cells positive for caspase 3 are extremely rare due to the high efficiency of phagocytosis of apoptotic cells by microglia (see Urbán et al., 2016).

Reviewer #1 (Significance (Required)):
General assessment:
This study concludes that aNSCs do not respond to the intermittent fasting. This expands and supplements previous findings that suggest that the intermittent fasting promotes adult neurogenesis by increasing survival and/or proliferation in the Subgranural Zone. The study is well designed, however, over-extends its conclusions beyond a specific fasting paradigm and does not acknowledge serious limitations in the experimental design and analyses. In fact, until major revision is done, which would rule out that the absence of effects of fasting on aNSCs is not due to false negative results, many conclusions from this study cannot be accepted as valid.
Advance:

As mentioned above, the study has a potential to advance our understanding of how fasting affects neurogenesis and fills the knowledge gap of how fasting specifically affects the stem cells. However, unless the study addresses its limitations, its conclusions are not convincing.
Audience:
This study would be particularly interesting for the niche readers from the neurogenesis field. However, the study can also be interesting for researchers in metabolomics and dietology.
My expertise:
adult neurogenesis, neural stem cells, dietology, metabolism
We disagree with the reviewer and find our conclusions well balanced, as we acknowledge our results are to be compared only with similar IF protocols. We also do not believe our results can be attributed to a false negative, as we consistently observe the same with different strains and protocols, always with sufficient animals to make our counts conclusive.
We nevertheless thank the reviewer for assessing our paper and for the advice to improve it. We hope that the reviewer will maintain the same level of scrutiny and scepticism with all IF-related papers.

Reviewer #2 (Evidence, reproducibility and clarity (Required)):
In this manuscript, Gabarro-Solanas et al. question the suitability of IF (Intermittent fasting - non-pharmacological strategy to counteract ageing, which has been previously shown to increase the number of adult-born neurons in the dentate gyrus of mice) as a pro-neurogenic intervention, since IF treatment did not stimulate adult hippocampal neurogenesis, neither at the stem cell level nor on immature and/or dividing neurons. The Authors used a tamoxifen inducible transgenic model (Glast-CreERT2;RYFP mice) to trace neural stem cell lineage and found that IF did not enhance neural stem cell proliferation, nor the abundance of immature, DCX+ neurons. Three-months of IF failed to increase the number of new adult-born neurons (NeuN+/YFP+), while one month of IF significantly reduced the number of new adult-born neurons.
The study appears technically sound, including many different approaches in order to reach its conclusions.
For instance, tamoxifen has been reported to impair various physiological processes, including neurogenesis (Smith et al., 2022), and most studies on adult hippocampal neurogenesis use the C57BL/6J strain of mice; hence, the use of Tamoxifen or that of the GlastCreERT2;RYFP model may have underscored these observations. However, to account for this potentially confounding factor, the Authors characterised the effect of their IF treatment in C57BL/6j mice, also reporting no evident effects of IF as a pro-neurogenic intervention.
I think the study was carefully planned and the analyses well done. Several possible variables were considered, including sex, labelling method, strain, tamoxifen usage or diet length. Several controls were performed in other organs and tissues (liver, fat) to establish the fasting protocol and to check its effects.
Data are presented in a clear way. Quality of images is high level.
In general, it appears as a highly reliable paper reaching an authoritative conclusion for the absence of effect of IF on adult neurogenesis.
Major comments:
I think that the key conclusions are convincing and no further experiments are required.

The methods are presented in such a way that they can be reproduced, and the experiments adequately replicated with proper statistical analysis.
We thank the reviewer for the encouraging remarks and the appreciation of our efforts.

Minor comments:
Prior studies are referenced appropriately, both regarding the IF protocols and the adult neurogenesis modulation.
Line 288 - a reference is incomplete (Dias); integrate with: (Dias et al., 2021)
We will re-format the reference, thanks for spotting the mistake.

There is one concept that is not expressed in the manuscript. Maybe it is not strictly necessary, but I think can be useful to mention it here. It is the fact that most information currently available strongly indicates that adult neurogenesis in humans is not present after adolescence. Of course the research described here is carried out on mice, and in the manuscript it is stated many times that adult hippocampal neurogenesis is strongly decreasing with age, also due to age-related stem cell depletion. Yet, it seems that in humans the exhaustion of such a process can start after adolescence. We know that a sort of controversy is currently present on this subjects, because DCX+ neurons can be detected in adult and old human hippocampi. Yet, it is also clear that there is no substantial cell division (stem cells are depleted) to sustain such hypothetical neurogenesis. Hence, it has been hypothesized that non-newlyborn, "immature" neurons can persist in the absence of cell division, as it has been well demonstrated in the cerebral cortex (see La Rosa et al., 2020 Front Neurosci; Rotheneichner et al., 2018, Cereb Cortex).
This point can be important in the case someone want to use dietary approached such as IF (or any other pharmacological treatment) to stimulate neurogenesis in humans.
We agree with the reviewer and also find this a very interesting and timely topic. However, we find it a bit far from our results and would prefer not to comment on it in the context of the current paper.

Reviewer #2 (Significance (Required)):
The significance of this study relies on the fact that adult neurogenesis field (AN) has been often damaged by the search of "positive" results, aiming at showing that AN does occur "always and everywhere" and that most internal/external stimuli do increase it. This attitude created a bias in the field, persuading many scientists that a result in AN is worthy of publication (or of high impact factor publication) only when a positive result is found.
Personally, I found particularly meaninful the last sentences of the Discussion (reported below), which might seem "off topic" in a research paper, while - I think - underline the real significance of the manuscript:
"In addition, publication bias might be playing a role in skewing the literature on fasting and neurogenesis towards reporting positive results.
In some reviews, even studies reporting no effect are cited as evidence for improved neurogenesis upon IF. Reporting of negative results, especially those challenging accepted dogmas, and a careful and rigorous evaluation of the publications cited in reviews are crucial to avoid unnecessary waste of resources and to promote the advancement of science."

Revision Plan

Reviewer field of expertise - keywords: adult neurogenesis, brain structural plasticity, non-newly born immature neurons, comparative neuroplasticity.

We are very happy that the reviewer shares our concern with the biased publication of positive results in the field. We hope our work (and that of Roberts et al., 2022) will encourage other labs to publish their negative results.

Reviewer #3 (Evidence, reproducibility and clarity (Required)):

In this manuscript, Gabarro-Solanas et al. investigate the effects of intermittent fasting (IF) on adult hippocampal neurogenesis in young adult mice. IF has been reported to increase the number of adult-born neuron in the hippocampus, a region that is important for learning and memory. However, it is not well understood what stages of adult neurogenesis are regulated by IF. To address this, the authors utilized lineage tracing and label retention assays in mice undergoing an IF diet. The authors used 2 months old Glast-CreERT2;RYFP mice in combination with Edu label retention to characterize adult NSCs and placed these mice on 1 and 3 months of IF. Despite seeing a decrease in neural stem cell proliferation with age, the authors did not observe a change due to diet. The authors then used immunohistochemistry to characterize changes in cell proliferation, neuroblasts, and new neurons following 1 month and 3 months of IF. Only 1 month of IF seemed to decrease the number of new neurons; however, by 3 months the neuronal output was the same. There were no differences in neuroblasts or cell proliferation due to diet. Gabarro-Solanas et al. conclude that IF transiently and mildly inhibits neurogenesis. Due to contradicting results, the authors then try to determine what variables (sex, labeling method, strain, tamoxifen usage, or diet length) could be affecting their data. The authors saw no substantial differences due to any of their variables.

Major Points

1. The authors analyze NSCs homeostasis and neurogenesis in young adult mice and do not observe any significant changes with their chosen alternate day intermittent fasting paradigm. However, a lot of the data and cell counts appears to be highly variable between animals in the same group. At times, there is an order of magnitude difference between the highest and lowest counts (e.g. Figure 2C,E). According to the method section, it appears that the authors predominantly analyzed a single DG (section?) for most immunostainings, which may explain the large variability in their data. If this is indeed the case, it is insufficient to quantify only a single section for each animal. The authors should quantify several DG sections for each mouse from a pre-defined range along the rostral-caudal axis of the hippocampus in accordance with a standard brain reference atlas. There are also several quantifications, especially of Ki67 where several individuals appear to have no Ki67+ (Figure 3B, 6D) NSCs. These findings are surprising given the still young age of these mice and may be another reflection of the limited brain sections that were analyzed.

The counts are indeed very variable. The counts were made on 1 to 4 DG sections (counted in full), depending on the staining. We will more clearly disclose this information in the revised version. In addition, we will re-count the neuronal output after fasting using stereology. Regarding the very low number of Ki67+ aNSCs, our counts are lower than those in other publications because we are much more stringent with our aNSC identification. Instead of using merely Sox2 (which also labels IPCs), we rely on the presence of a radial GFAP+ process.

2. There appear to be significant cutting or imaging artifacts across most fluorescent images further raising concerns regarding the accuracy of the quantifications (e.g. Figure 3D, 4C,E, 6B) and publication quality of the images and data. Importantly, uneven section thickness, either from cutting artifacts or imaging issues, may lead to inaccurate cell quantifications a could, possibly, account for the high variability. This issue would further exacerbate concerns regarding the quantification of a single DG section for each animal.
We only processed those samples that passed our QC after sectioning, meaning any unevenly cut brains were never considered (or stained). The stitched images do show artifacts (lower signal in the image junctions), particularly in the NeuN staining. However, this did not affect quantifications, as the measured levels were always clearly above the threshold to consider a cell positive, regardless of the position within the image. The images were cropped to improve the visualisation of NSCs, and to avoid the display of empty tiles. A low magnification image will be provided in the revised version to show that there were no staining artifacts.

3. It is unclear how NSCs were counted in the B6 mice (Fig 6D,E). The authors only provide a description for the Glast-CRE mice, where they used YFP labeling and GFAP. We assume they performed Sox2/GFAP or Nestin labeling, however, this is not clear at all. The authors should describe their methodology and provide representative images.
We used GFAP, location and morphology to count aNSCs in non-YFP mice. We will make this clear in the text and will also add one more count using Sox2, GFAP and Nestin to identify aNSCs.

4. NSC populations represent a heterogenous group of stem cells with different replicative properties. As such, the Glast-Cre approach used for the majority of this study may represent a specific subset of NSCs. In line with the previous point, we recommend the authors complement their NSC counts with Sox2/GFAP and Nestin immunostainings.
aNSCs labelled with Glast-Cre are the great majority of aNSCs (>90%) in both ad libitum fed and fasted mice. The data will be included in the revised version. Nevertheless, we will add counts using Sox2, GFAP and Nestin for key experiments.

5. Stress is a significant negative regulator of neurogenesis. Is it possible that the IF mice display higher stress level which could counteract any beneficial effects of the IF intervention. The authors should provide some measures of stress markers to rule out this potential confounding factor in their IF paradigm.
This is a great suggestion. We will collect blood from control and fasted mice and measure the levels of stress factors (e.g. corticosterone). We will include the data in our revised version.

Minor Point
1. The authors state that "Experimental groups were formed by randomly assigning mice from different litters within each mouse strain and all experiments were conducted in male and female mice". Given that neurogenesis, especially at young ages, is highly sensitive to the exact age of the mice, the authors should provide a rationale why animals from different litters instead of littermate controls were used in these experiments.

Littermate controls were always used in the experiments. But also, more than one litter was used for each experiment, since one litter was never generating enough mice for the experiments. We will clarify this point in text.

2. Currently, the statistical tests are only described in the method section, however it would be helpful if this information to be integrated into the figure legend as well. Additionally, the authors provide individual data points for some but not all bar graphs (eg Figure 1D).
We will consider including the statistical information in the figure legend, provided there is not a maximum length for figure legends. In the case of figure 1D, data points are not shown because of how the food intake was calculated: as an average per cage instead of per animal (included in the materials and methods). We therefore do not consider it useful to show the datapoints in the final version of the manuscript, but will provide them for the reviewer.

3. Cell counts per AU is a rather unorthodox unit. With a representative selection of tissue for each animal, the authors could avoid the need to normalize to the DG length and may be able to extrapolate an estimate of cell counts for the entire DG instead.
Thanks for the suggestion. Our arbitrary units (AU) were in fact already equivalent to cells per mm of DG, and we have updated our graphs to reflect this.

4. In Figure 4D, the authors highlight a few NSC with arrowheads. At a quick glance this is rather confusing as it appears that the authors only counted 3 NSCs in each picture. It may be a better option to show a zoomed in picture to highlight an example of a representative NSC.
Examples of representative NSCs are already shown in Fig 2. With this image, we intended to show a larger number of NSCs. We realise the arrows only pointed to some of them, making the message confusing. We will consider removing them from the figure in the revised version.

5. In Supplementary Figure S6, the authors should complement the quantification of the nuclei with representative images.
We will include representative images in Figure S6.

6. For the daytime IF, did the authors assess weights, food intake, RER as well liver/fat measurements similar to night-time IF? If so, this data should be provided in the supplement.
We do have data for the daytime IF in the metabolic cages, which was taken from mice housed in groups (during the preliminary phase of our study). We also have the weight and data on neurogenesis, which we will show as a supplement.

Reviewer #3 (Significance (Required)):
The authors are commended for compiling a manuscript on what is commonly considered 'negative data', that, at the same time, are also contradicting independent reports on the effects of IF on neurogenesis. The studies outlined in this manuscript are comprehensive and mostly well designed. Given the broad, growing interest in dietary restriction as an aging intervention the study is timely.
We thank the reviewer for the positive assessment of the significance of our work.

Revision Plan

Reviewer #4 (Evidence, reproducibility and clarity (Required)):

Summary:

In this manuscript, Gabarró-Solanas et al. tested the effect of intermitted fasting (IF, every-other-day fasting) on adult neural stem cells and neurogenesis. They demonstrate that the paradigm they have used does not affect NSC activation or maintenance, and does also not promote neurogenesis. As previous reports showed increased neurogenesis with IF, the authors controlled for various parameters such as mouse strain, sex, and diet length. They also used different methods of identification of newborn neurons, such as tamoxifen-induced lineage-tracing versus birth-dating with thymidine-analogues to substantiate their findings.

Major comments:

This study is very well done with carefully designed and controlled experiments. The manuscript reads nicely and the data are presented in a clear way, making it easy to follow. The authors have done a "tour-de force" to rule out confounding factors that might influence their findings that IF does not affect NSCs nor neurogenesis.

The claims and conclusions are supported by the data. The methods are clearly described and should allow to reproduce the data independently. The number of replicates (i.e. the number of mice analyzed) is impressive and statistical analysis is adequate.

The major findings, namely that the chosen IF does not affect NSCs and neurogenesis is not in line with some previous studies. Despite a careful ruling out of potentially confounding factors (see also "significance" below), it remains unclear why other studies have found an increase in neurogenesis with IF. As each of these studies has some specific experimental design, it is difficult to judge these data in the context of previous data without going through all the details of the other studies. It would thus be a great help for the reader if the authors could provide a table or schematic, which lists the major parameters of each of these studies, such as detailed paradigm of IF, age of mice at start, sex, duration of the intervention, method of identification of NSCs and neurogenesis etc.

This is a very good suggestion, and we had already created such a table. We, however, consider that it might be better suited for a review on the effects of IF on neurogenesis than for this work. We will include the table in our response to the reviewers together with our revised version.

Two points that the authors have not discussed might also be worth mentioning in the discussion part:

1.) The mice in the night-time IF were single caged, could there be a potential negative effect on neurogenesis that would mask the presumably beneficial effect of IF? Although the controls were also single caged, the stress of social isolation might play a role?

The mice were only single caged for the metabolic phenotyping, but not for the neurogenic counts. We will make this clearer in the text. In any case, we do agree that stress might play a role and we will measure stress levels in the control and fasted mice and will include this data in the revised version.

2.) The IF mice gained the same weight over time (Fig. S2), but had a ~20% reduction in overall calory intake. This would be explainable by a reduction in energy expenditure, but the overall

activity was also not significantly changed (Fig. S1). Can the authors speculate why they reach the same weight with less calories?
We also found this surprising and were expecting a reduction in the overall activity of the fasted mice. We do not have an explanation for this discrepancy, but perhaps stress levels might explain part of it (we will check stress levels in the revised version). We will also look at whether energy expenditure and activity levels changed over time.

Minor comments:
1.) It would be nice to replace the arbitrary units (AU) in the graphs were this is used (e.g. Fig. 2F, 3C, 4B, D and F etc) to the actual number of cells per a certain μm DG, so that the number of cells can be put in context and compared between the figures.
Yes, our AU already corresponded to mm and we will update our figures accordingly.

2.) Fig 3 D: can the authors also show the Ki67 channel to illustrate how it looks after a 3 month IF?
We find it does not help much, as Ki67+ cells are mostly IPCs and that data is already shown in Fig. 4A. We will nevertheless include the image in our response to the reviewers together with our revised version.

3.) Fig.4E: the NeuN staining looks strangely interrupted, this might be due to tile-stitching? In that case, it would be better to either only show one segment or to try to get a better stitching algorhythm.
It is indeed because of the tile-stitching and uneven illumination. However, this did not affect the counts, as already discussed in the response to reviewer #3 (major point #2).

4.) Fig.6 D shows a minus axis in Y-axis, this should only been shown from 0 to positive values, as it is a percentage of cells and cannot be negative.
True, thanks for spotting this. We will correct the graphs in the revised version.

5.) Fig.6 B: the same problem with the NeuN staining as mentioned under point 3. This should be improved.
As with point 3, the stitching did not affect the quantification. We find it more accurate to show the image with the stitching, as that was the one used for quantification. We will provide a new picture with lower magnification to better show the quality of the staining.

6.) Fig. S6B: maybe add a comment in the result part or in the figure legend that a 10 day chase after an EdU pulse is not the classical protocol to look at mature NeuN positive neurons. But apparently enough newborn neurons were already NeuN positive for this quantification.
We fully agree 10 days is not the standard for neuronal identification. We did find neurons after the 10-day chase but in low numbers. We will add a comment in the text of the revised version to clarify this.

7.) The authors refer to personal communications with M. Mattson and S. Thuret to underline that circadian disruption is not enough to explain the differences (line 367 onwards). Can they refer the reader to published data instead?
While the results are published in their papers, the methods did not specify the time at which the food was added/removed for the IF protocol. That is why we refer to personal communication. Further showing that disruption of circadian rhythms is not enough to explain the difference in outcome of the IF protocol, we will show the data for the 1-month daytime IF, which again does not increase adult neurogenesis (reviewer #3, minor point #6).

Reviewer #4 (Significance (Required)):
Given the great interest in the seemingly positive effects on health of IF in general, and also for increasing neurogenesis, it is important to better understand the mechanism of this intervention. The study by Gabarró-Solanas et al. clearly demonstrates that IF is not a universal, "works all the time" way of increasing neurogenesis. The study is very well done, with well controlled and measured parameters. It shows that a physiological interference such as IF might depend on many factors and might be less robust across laboratories than anticipated. This study is a very good example that all the details of the experimental settings need to be taken into consideration and are ideally reported with every IF study. It is also a good example how to follow up "no effect" data in a way that they are conclusive.
The significance of this study is to point out that IF as a strategy to increase neurogenesis needs to be reconsidered. It raises the questions how IF can be beneficial in some studies and not in others, asking for more experiments to better understand the detailed mechanisms of IF action. In a systematic approach, this study rules out some of the potentially confounding factors and shows that at least with the chosen IF paradigm, these factors are not the reason for not seeing increased neurogenesis. The study is thus of clear interest for the neurogenesis field and will also need to be considered by the broader field of IF research, although it speaks against the beneficial effects of IF. It might have the potential to bring together the different study authors who did or did not see increased neurogenesis with IF and discuss together the non-published details of their study design to advance the field.
We thank the reviewer for the positive assessment of our work and for acknowledging its importance for the broader field of IF research.

3. Description of the revisions that have already been incorporated in the transferred manuscript

Please insert a point-by-point reply describing the revisions that were *__already carried out and included__* *in the transferred manuscript. If no revisions have been carried out yet, please leave this section empty.*

4. Description of analyses that authors prefer not to carry out

Please include a point-by-point response explaining why some of the requested data or additional analyses might not be necessary or cannot be provided within the scope of a revision. This can be due to time or resource limitations or in case of disagreement about the necessity of such additional data given the scope of the study. Please leave empty if not applicable.

Please, find more detailed reasons in the main response to each reviewer.

- Behavioural tests such as open field or elevated T maze to assess stress and anxiety levels: we think that a more accurate way of assessing stress would be through the analysis of stress hormone levels in blood. This alternative measurement will be included in the revised version of the manuscript.
- Analysis of parameters such as astrocyte reactivity, inflammation or microglia activation to evaluate whether the used IF protocol elicits cellular or molecular changes in the CNS: after revising the literature, we found no evidence that IF alone influences any of those parameters.
- Changing the title: we think that our title and abstract faithfully reflect our data and conclusions.
- Assessing potential effects on symmetric vs. asymmetric NSC divisions: the images in Fig 2B cannot be taken as evidence of a change in plane of cell division upon IF. Additionally, there are no effects on NSC maintenance which would be expected after a change in the mode of cell division, so we consider this analysis unnecessary.
- Quantifying differences in Fasn expression in NSCs from control and IF animals: we think there is no need to test for Fasn expression since there is no effect on NSCs.
- Determining apoptosis by activated Caspase 3 staining: we have seen in previous work that picnotic nuclei reflect cell death in the DG more accurately (see Urbán et al., 2016).
- Discussing human neurogenesis: we find this is out of the scope of our paper.
- A table summarising previous publications: we think that this would be more suited for a review article.
- Including the Ki67 channel in Fig. 3D: we think it does not help the visualisation of the NSCs.

List of references used in the response to reviewers:

Anson, R. M. *et al.* Intermittent fasting dissociates beneficial effects of dietary restriction on glucose metabolism and neuronal resistance to injury from calorie intake. *Proceedings of the National Academy of Sciences* **100**, 6216–6220 (2003).

Bok, E. *et al.* Dietary Restriction and Neuroinflammation: A Potential Mechanistic Link. *International Journal of Molecular Sciences* **20**, 464 (2019).

Cignarella, F. *et al.* Intermittent Fasting Confers Protection in CNS Autoimmunity by Altering the Gut Microbiota. *Cell Metabolism* **27**, 1222-1235.e6 (2018).

Dai, S. *et al.* Intermittent fasting reduces neuroinflammation in intracerebral hemorrhage through the Sirt3/Nrf2/HO-1 pathway. *Journal of Neuroinflammation* **19**, 122 (2022).

Dias, G. P. *et al.* Intermittent fasting enhances long-term memory consolidation, adult hippocampal neurogenesis, and expression of longevity gene Klotho. *Mol Psychiatry* 1–15 (2021).

Goodrick, C. L., Ingram, D. K., Reynolds, M. A., Freeman, J. R. & Cider, N. Effects of intermittent feeding upon body weight and lifespan in inbred mice: interaction of genotype and age. *Mechanisms of Ageing and Development* **55**, 69–87 (1990).

Gudden, J., Arias Vasquez, A. & Bloemendaal, M. The Effects of Intermittent Fasting on Brain and Cognitive Function. *Nutrients* **13**, 3166 (2021).

Lee, J., Seroogy, K. B. & Mattson, M. P. Dietary restriction enhances neurotrophin expression and neurogenesis in the hippocampus of adult mice. *Journal of Neurochemistry* **80**, 539–547 (2002).

Rangan, P. *et al.* Fasting-mimicking diet cycles reduce neuroinflammation to attenuate cognitive decline in Alzheimer's models. *Cell Reports* **40**, 111417 (2022).

Roberts, L. D. *et al.* The 5:2 diet does not increase adult hippocampal neurogenesis or enhance spatial memory in mice. 2022.10.03.510613 BioRxiv Preprint (2022).

Song, M.-Y. *et al.* Energy restriction induced SIRT6 inhibits microglia activation and promotes angiogenesis in cerebral ischemia via transcriptional inhibition of TXNIP. *Cell Death Dis* **13**, 449 (2022).

Urbán, N. *et al.* Return to quiescence of mouse neural stem cells by degradation of a proactivation protein. *Science* **353**, 292–295 (2016).

1st Editorial Decision 11th Apr 2023

Dear Dr. Urbán Avellaneda,

Thank you for the transfer of your research manuscript to EMBO reports from Review Commons. I apologize for the delayed response, but I have now read and discussed your manuscript with the other members of our editorial team, along with the reports of the referees who evaluated your manuscript, as well as your revision plan.

Given the constructive comments of the referees, who acknowledge that the findings are potentially interesting but also identify a number of limitations in the study, we would like to invite you to revise your manuscript with the understanding that the referee concerns (as detailed in their reports) must be fully addressed and their suggestions taken on board. Please address all referee concerns in a complete point-by-point response. Acceptance of the manuscript will depend on a positive outcome of a second round of review. It is EMBO reports policy to allow a single round of revision only and acceptance or rejection of the manuscript will therefore depend on the completeness of your responses included in the next, final version of the manuscript. If you have any questions or comments, we can also discuss the revisions in a video chat, if you like.

We realize that it is difficult to revise to a specific deadline. In the interest of protecting the conceptual advance provided by the work, we usually recommend a revision within 3 months (July 10th). Please discuss with me the revision progress ahead of this time if you require more time to complete the revisions.

*****
IMPORTANT NOTE:
We perform an initial quality control of all revised manuscripts before re-review. Your manuscript will FAIL this control and the handling will be DELAYED if the following APPLIES:

1) If a data availability section providing access to data deposited in public databases is missing. If you have not deposited any data, please add a sentence to the data availability section that explains that (see below for more information).

2) If your manuscript contains statistics and error bars based on n=2. Please use scatter plots in these cases. No statistics should be calculated if n=2.

When submitting your revised manuscript, please carefully review the instructions that follow below. Failure to include requested items will delay the evaluation of your revision.
*****

When submitting your revised manuscript, we will require:

1) A .docx formatted version of the manuscript text (including legends for main figures, EV figures and tables). Please make sure that the changes are highlighted to be clearly visible.

2) Individual production quality figure files as .eps, .tif, .jpg (one file per figure).
Please download our Figure Preparation Guidelines (figure preparation pdf) from our Author Guidelines pages https://www.embopress.org/page/journal/14693178/authorguide for more info on how to prepare your figures.

3) A .docx formatted letter INCLUDING the reviewers' reports and your detailed point-by-point responses to their comments. As part of the EMBO Press transparent editorial process, the point-by-point response is part of the Review Process File (RPF), which will be published alongside your paper unless you opt out of this (please see below for further information).

4) A complete author checklist, which you can download from our author guidelines (). Please insert information in the checklist that is also reflected in the manuscript. The completed author checklist will also be part of the RPF (please see below for more information).

5) Please note that all corresponding authors are required to supply an ORCID ID for their name upon submission of a revised manuscript (). Please find instructions on how to link your ORCID ID to your account in our manuscript tracking system in our Author guidelines
()

6) We replaced Supplementary Information with Expanded View (EV) Figures and Tables that are collapsible/expandable online. A maximum of 5 EV Figures can be typeset. EV Figures should be cited as 'Figure EV1, Figure EV2" etc... in the text and their respective legends should be included in the main text after the legends of regular figures.

- For the figures that you do NOT wish to display as Expanded View figures, they should be bundled together with their legends

in a single PDF file called *Appendix*, which should start with a short Table of Content. Appendix figures should be referred to in the main text as: "Appendix Figure S1, Appendix Figure S2" etc. See detailed instructions regarding expanded view here:

- Additional Tables/Datasets should be labeled and referred to as Table EV1, Dataset EV1, etc. Legends have to be provided in a separate tab in case of .xls files. Alternatively, the legend can be supplied as a separate text file (README) and zipped together with the Table/Dataset file.

7) Please note that a "Data availability" section at the end of Materials and Methods is now mandatory. In case you have no data that require deposition in a public database, please state so instead of refereeing to the database: "Our study includes no data deposited in public repositories." under the heading "Data availability".
See also). Please note that the Data availability statement is restricted to new primary data that are part of this study.

8) We request authors to consider both actual and perceived competing interests. Please review the new policy () and update your competing interests statement if necessary. Please name this section 'Disclosure and competing interests statement' and place it after the Acknowledgements section.

9) Figure legends and data quantification:
The following points must be specified in each figure legend:

- the name of the statistical test used to generate error bars and P values,
- the number (n) of independent experiments (please specify technical or biological replicates) underlying each data point,
- the nature of the bars and error bars (s.d., s.e.m.)
- If the data are obtained from n {less than or equal to} 2, use scatter plots showing the individual data points.

Discussion of statistical methodology can be reported in the Materials and Methods section, but figure legends should contain a basic description of n, P and the test applied.

See also the guidelines for figure legend preparation:
https://www.embopress.org/page/journal/14693178/authorguide#figureformat

- Please also include scale bars in all microscopy images.

10) We now request publication of original source data with the aim of making primary data more accessible and transparent to the reader. Our source data coordinator will contact you to discuss which figure panels we would need source data for and will also provide you with helpful tips on how to upload and organize the files.

11) Our journal encourages inclusion of *data citations in the reference list* to directly cite datasets that were re-used and obtained from public databases. Data citations in the article text are distinct from normal bibliographical citations and should directly link to the database records from which the data can be accessed. In the main text, data citations are formatted as follows: "Data ref: Smith et al, 2001" or "Data ref: NCBI Sequence Read Archive PRJNA342805, 2017". In the Reference list, data citations must be labeled with "[DATASET]". A data reference must provide the database name, accession number/identifiers and a resolvable link to the landing page from which the data can be accessed at the end of the reference. Further instructions are available at .

12) Please also note our reference format:
.

13) We now use CRediT to specify the contributions of each author in the journal submission system. CRediT replaces the author contribution section, which should be removed from the manuscript. Please use the free text box to provide more detailed descriptions. See also guide to authors:
.

14) As part of the EMBO publications' Transparent Editorial Process, EMBO reports publishes online a Review Process File to accompany accepted manuscripts. This File will be published in conjunction with your paper and will include the referee reports, your point-by-point response and all pertinent correspondence relating to the manuscript.

You can opt out of this by letting the editorial office know (emboreports@embo.org). If you do opt out, the Review Process File link will point to the following statement: "No Review Process File is available with this article, as the authors have chosen not to make the review process public in this case."

We would also welcome the submission of cover suggestions, or motifs to be used by our Graphics Illustrator in designing a cover.

I look forward to seeing a revised version of your manuscript when it is ready. Please let me know if you have any questions or comments regarding the revision.

Yours sincerely,

Ioannis Papaioannou, PhD
Editor
EMBO reports

Authors' Response

Manuscript number: RC-221212
Corresponding author(s): Noelia, Urbán

1. General Statements [optional]

This section is optional. Insert here any general statements you wish to make about the goal of the study or about the reviews.

2. Description of the planned revisions

We provide here a point-by-point response to the reviewer's comments, with our answers and comments in blue. Updated responses after full revision are shown in green. Updated text is highlighted in blue in the manuscript.

Reviewer #1 (Evidence, reproducibility and clarity (Required)):
Summary:
In this study, mice were exposed to a specific form of so-called Intermittent Fasting (IF) and the effects of IF on adult neogenesis in the hippocampus were determined. The specific IF protocol used had no effect on activation, proliferation, or maintenance of adult Neural Stem Cells (aNSCs) and displayed a decrease in number of new neurons in the neurogenic niche but only after 1 month of the IF protocol. These results contrast previously published results from multiple studies that concluded that IF promotes survival of new neurons and by extension promote adult neurogenesis. The unresponsiveness of aNSCs or their immediate cell progeny, the Intermediate Neural Progenitors (IPCs), to IF is a novel finding. The authors make several relevant points in the discussion about the publication bias towards positive results (or omission of negative results), which may reinforce established dogmas. However, the presented results did not convincingly demonstrate that the absence of effects of IF on aNSCs or adult neurogenesis is simply not a result of a specific IF paradigm, which is not robust enough to elicit changes in adult neurogenesis. In other words, there is a lack of positive controls and alternative protocols that would rule out that the observed absence of effects is not a consequence of type II error (the error of omission), or more colloquially, a consequence of false negatives.
We thank the reviewer for acknowledging the importance and novelty of our findings. On them being the result of a specific IF paradigm, we must point out that we used the same IF paradigm as in previous studies that had shown changes in neurogenesis upon IF. We do not claim that IF is unable to increase neurogenesis in all conditions, but report that IF is not a reliable method to increase adult neurogenesis (in particular, every-other-day intermittent fasting with food re-administration in the evening). We have repeated the experiment multiple times in different strains, always with enough animals to make our experiments conclusive and we never observed an increase in adult neurogenesis, effectively ruling out that our results are a false negative. Of note, even if other protocols might indeed increase neurogenesis (which we never claimed cannot) that would not make our results a false negative.

Authors' Response

Major Comments:

1. Protocol-driven absence of effects: The absence of IF effects on aNSCs and IPCs observed in this study does not lend it the authority to conclude that aNSCs are resilient to IF or all IF paradigms and protocols. The absence of IF effects on aNSCs and neurogenesis could be specifically related to the chosen IF paradigm. Indeed, not all previous studies that observed IF-driven effects on adult neurogenesis used the same "night-time every-other-day fasting" protocol chosen in this study. For example, Brandhorst et al., 2015 (cited in this paper) used 4 days of IF 2x per month and observed an increase of DCX+BrdU+ cells. On the other hand, certain previous studies used the same or similar IF protocol used here, but often with longer duration or with a post-fasting ad libitum feeding period, which may be responsible for the pro-neurogenic or pro-survival effects. In fact, the authors acknowledge this in the discussion (page 7, lines 289-290 and 292-294). Why would the authors then not include similar feeding/IF paradigm in their study and determine if these would generate effects on survival of new neurons but also on aNSCs and/or IPCs?
As just stated above, we never claimed that aNSCs are resilient to all IF paradigms. We refer to fasting in general in the introduction but quickly focus on every-other-day fasting throughout the paper and directly compare our results only to similar IF paradigms. We chose the most commonly used IF paradigm that had been shown to increase adult neurogenesis. As the reviewer points out, we speculate in the discussion that a refeeding period may explain the differences between our results and others. This is because a post-fasting ad libitum period was introduced in the study published in Dias et al. 2021. We are currently analysing a new experiment in which we replicate the IF protocol in that study, which we will include in our revised version.
The analysis of the experiment where we add a re-feeding period of 5 days of ad libitum access to food (as in Dias et al. 2021) shows no difference between control and fasted mice in proliferating cells or neuroblasts, regardless of refeeding (Figure 6 I-K, lines 306-311). Regarding the longer duration, we also want to point out that we had already analysed mice after 4 months of IF to match previous reports (instead of the 3 that were our initial observation). This data can be found in Figure 6 A-H and lines 293-305.

In addition, the authors acknowledge that the chosen IF paradigm may have affected the stress levels or behaviour of mice (page 9, lines 372-378). Why did they not test if their IF protocol does not increase stress or anxiety of mice by simple behaviour tests such as open field or elevated T maze?
While testing all possible causes for the lack of positive results in our experiments is not viable, we do agree with the reviewer that stress levels might indeed influence the outcome of the experiments. We will collect blood from ad libitum-fed and fasted mice to analyse the levels of stress hormones (e.g. corticosterone). The results will be included in our revised version. These measurements will give us a more accurate reading of stress levels than behavioural tests. Of note, regardless of the outcome of this experiment, our conclusions will remain identical. We will not be able to compare stress levels with previous publications, as they were not tested. And if the protocol did increase stress levels, it would still argue that IF is not a reliable method to

increase neurogenesis (as presumably might or might not increase stress to levels that affect neurogenesis).
We have now measured corticosterone levels in mice after one cycle of IF as well as after one full month of IF (Figures 1I and EV1M, lines 155-158). Our results show a transient increase in stress on the first day of fasting (EV1M) and no differences between control mice and mice on IF on either fasting or refeeding days after one month of IF. Since corticosterone levels measure acute stress, we conclude that mice adapt to IF after the initial cycle. We also never observed differences in grooming behaviour or repetitive behaviours between IF and control mice during our routine monitoring of the mice.

Alarmingly, the used IF protocol does not result in changes in final weight or growth curves (S.Fig.2), which is surprising and raises a question the used IF protocol is robust enough or appropriate.
We were also surprised by the lack of change in the final weight our IF mice respect to control. Differences in final weight between different labs despite using the exact same protocol are one of the reasons why we conclude that this IF paradigm is not a robust intervention. However, we are not the first ones to report little or no difference in weight upon IF in C57BL6/J mice (Goodrick et al., 1990 and Anson et al., 2003) and this would not be a reason to dismiss the experiment since the benefits in crucial circulating factors induced by IF seem to be independent of weight loss (Anson et al., 2003).
In addition to the above, we want to point out to further previous and new evidence that our IF protocol indeed works as intended. We had already shown that the RER ratio is shifted in fasted mice and that the IF protocol elicits the expected changes in fat browning and hepatocyte morphology (Figure 1E, J, K and Appendix 1). We now present further evidence of the effectivity of the IF protocol, as we observed both immediate and long-lasting changes in glucose levels (Figures EV1K and 1G) and an increase in ketogenic metabolism (shown as higher levels of Hydrobutyric acid) on fasting days both after one cycle and one month of IF (Figures EV1L and 1H). All found in lines 134-167.

Finally, the authors acknowledge that their own results do not support well-established findings such as aging-related reduction in number of aNSCs (page 4, lines 177-179). This again questions whether the selected protocols and treatments are appropriate.
As we already discuss, we believe this might be due to a difference between strains in the time when aNSC numbers decline. Nevertheless, we will complement our current data by counting the number of aNSCs at 1 and 3 months post-tamoxifen (3 and 5 month old mice) using GFAP, Sox2 and Nestin triple stainings (as suggested by another reviewer).
The quantification of NSCs with another method (as above) confirmed the lack of a sharp decline in NSC number between 3 and 5 months in this particular strain (Figure EV2E-H, lines 210-218).

2. Lack of topic-specific positive controls: The authors successfully demonstrated that the used IF protocol differentially impacts the adipose tissue and liver, while also inducing body weight fluctuations synchronized with the fasting periods. However, these peripheral effects outside the

CNS do not directly imply that the chosen IF protocol is robust enough to elicit cellular or molecular changes in the hippocampus. The authors need to demonstrate that their IF protocol affects previously well-established CNS parameters associated with fasting such as astrocyte reactivity, inflammation or microglia activation, among other factors. In fact, they acknowledge this systemic problem in the discussion (page 8, lines 359-360).

We fully agree with the reviewer in that even though the chosen IF protocol induces peripheral effects, it is not robust enough to elicit cellular or molecular changes in the hippocampus, and this is precisely the message of our paper. We have looked for references showing the influence of IF on astrocyte reactivity or microglia activation, but the studies we found so far look at the effects of IF and other forms of fasting in the CNS in combination with pathologies such as Alzheimer's disease, Multiple Sclerosis, physical insults, or aging (Anson et al., 2003; Chignarella et al., 2018; Rangan et al., 2022; Dai et al., 2022. Reviewed in Bok et al., 2019 and Gudden et al., 2021). Fasting seems to reduce astrocyte reactivity, inflammation, or microglia activation in these pathological situations respect to the same pathology in ad libitum mice, but its effect in control, healthy mice is far less clear. In fact, the only reference that we could find where healthy mice were included in the analysis showed that these benefits only happened in the context of the injury (Song et al., 2022).

For the above reasons, we did not conduct any further experiments to test for brain-specific changes upon IF.

3. Problematic cell analyses: Cell quantification should be performed under stereological principles. However, the presented results did not adhere to stereological quantification. Instead, the authors chose to quantify specific cell phenotypes only in subjectively selected subsets of regions of interest, i.e., the Subgranular Zone (SGZ). This subjective pre-selection may have been responsible for the absence of effects, especially if these are either relatively small or dependent on anatomical sections of SGZ. For example, IF may exert effects on caudal SGZ more than on rostral SGZ. But if the authors quantified only (or predominantly) rostral SGZ, they may have missed these effects by biasing one segment of SGZ versus other. The authors should apply stereological quantification at least to the quantification of new neurons and test if this approach replicated previously observed pro-survival effects of IF. Also, the authors should describe how they pre-selected the ROI for cell quantification in greater details.

We did analyse only the more septal region of the hippocampus, which we will make clear in the text. As also suggested by other reviewers, we will include stereological counts of the neuronal output of aNSCs in the revised version. As for selecting the SGZ for aNSC counts, this is the standard in the field, as one of the criteria to identify aNSCs is precisely the location of their nucleus in the SGZ. Neuroblasts and new neurons were counted both in the SGZ and the granule cell layer. There was no subjective pre-selection of areas of interest since we counted the whole DG in each section and not a specific random region.

We re-quantified our samples using stereological principles along the whole rostro-caudal length of the hippocampus. For this, we established a collaboration with the group of Albert Giralt, who are experts in brain stereology. We attempted to use stereoinvestigator®, as this program is one of the gold standards in the field. However, it proved to be unsuitable for counting adult NSCs in the hippocampus, as the subgranular zone is too narrow for the program to provide appropriate

unbiased regions to count. We also attempted to use stereoinvestigator® for our counts on newborn cells and neurons. In this case, the low number of EdU+ cells again prevented us from being able to use the program. Instead, we followed stereological guidelines to count newly born cells throughout the dentate gyrus (as most other studies had done). We counted the whole area of the DG, as we had done for previous counts, but now in one every sixth section covering the whole rostro-caudal length of the DG. Our results show a small decrease in the number of EdU+ cells in IF mice respect to control, as well as a higher number of new cells in more anterior sections). The data is displayed in Figure 5G, 6C and EV5 (lines 288-290 and 300-302), and we have updated our material and methods (lines 577-585) to include the stereological counting of newly-born cells.

4. Alarming exclusion of data points: There appears to be different number of data points in different graphs that are constructed from same data sets. For example, in the 3-month IF data set in Figure 4, there are 14 data points for the graph of Ki67+ cells (Fig.4B), but 16 (or 17) data points for the graph of DCX+ cells (Fig.4D). How is that possible? If data points were excluded, what objective and statistical criteria were applied to make sure that such exclusion is not subjective and biased? In fact, the authors state that "Samples with poor staining quality were also excluded from quantifications" (page 12, line 528-529). Poor preparation of tissue is not only suboptimal but not a valid objective reason for data point exclusion. This major issue needs to be explained and corrected.
As we disclose in the methods, those stainings that did not work were excluded. This was done always before counting. Different samples were used in different counts because of the variability of staining quality between different antibodies. We will look back into the samples that failed in at least one of the stainings and exclude them from all counts, so that only samples for which all stainings worked are considered. These revised graphs will be provided in our revised version of the manuscript.
We have excluded from all counts those samples in which stainings for some of the markers had failed. This did not change the results except for the graph in Figure 6D, which conserved the same trend but lost the significance (see our point on small differences in point 3 and the discussion. The differences between the graphs can be seen by comparing the old and new versions of the figures and in addition, we show a few examples side by side in the figure for reviewers at the end of the revision plan.

5. Different pulse-and-chase time-points: One of the reasons why this study has found that aNSCs may not be responsive to IF could be the use of less appropriate pulse-and-chase time-points either after EdU or after Tamoxifen for cell lineage tracing. The authors observed that IF has negative effects on new neurons initially (Fig.4F). Similarly, it is well established that voluntary physical exercise affects SGZ adult neurogenesis only during the first 2 weeks. After this period, the neurogenic effects of exercise are diminished beyond observational detection (i.e., van Praag's and Kempermann's papers in the past 25 years). These two arguments suggest that the observed absence of aNSC responsiveness might be a consequence of the chosen EdU administration and the EdU pulse should not be administered 15 days after Tamoxifen/IF protocol start but earlier, in the first week of the IF protocol. In fact, the decreased

number of new neurons during the initial IF phase may not be only a consequence of reduced survival but of higher aNSC quiescence during the first week of the IF protocol.

We fully agree with the reviewer that BrdU or EdU pulses can give a biased view of the effects of any intervention on neurogenesis and that the EdU and Tamoxifen protocols would not allow us to detect an increase in neurogenesis during the first few days of IF. We cannot rule out that IF has a transient effect on aNSCs at some point of the treatment, but this hypothetical effect does not seem to have any consequences on neuronal output or aNSC maintenance. As for the effects on neurogenesis in the longer IF treatments, we used the same EdU protocol as in previous publications: administration after 2/3 months of IF and analysis after one month of chase.

Further to the above, the small and transient decrease in neurogenesis after one month is now not significant (after changing our statistics from t-test between IF and control to ANOVA comparing both diet and age, Figure 4F and lines 239-241). In any case, our conclusions remain the same and still agree that small, transient changes in neurogenesis might have been lost with our specific protocols.

6. Discussion needs more specificity and clarity: The authors claim that the absence of IF effects on neurogenesis is multi-layered (including the influence of age, sex, specific cell labelling protocols etc.) but they do not specifically address why certain studies did find IF-driven neurogenic effects while they did not. In addition, some statements and points in the discussion are not clear. For example, when the authors refer to their own experiments (page 8, lines 331-334), it is not clear, which experiments they have in mind.

We will double check our discussion and improve its clarity and direct comparison to other studies.

We have now indicated where in the manuscript were the experiments we referred to in that paragraph (Appendix 3, Figure 5 F-I and Figure 6A-H). We now further compare our data to that of Dias et al., where a refeeding period is added and more explicitly acknowledge that we cannot fully explain why our results differ to those of others: “While we matched the age of the mice with previous reports and saw no changes in stress levels (measured by corticosterone), many other variables remain untested that could explain discrepancies between labs.” (lines 419-422)

Minor comments:

1. Change in the title: The authors have shown that a very specific IF protocol does not affect aNSCs but initially decreases number of new neurons in SGZ. The title should reflect this. For example, it could state "Specific (night-time every-other-day) fasting does not affect aNSCs but initially decreases survival of new neurons in the SGZ".

We find our title, together with the abstract, clearly and faithfully represent our findings and would rather prefer to keep our current title unmodified.

We have decided to keep our title as is.

2. Data depiction: Data in 3 datasets were found not normally distributed (Fig. S5A, B and S6A) and were correctly analysed with non-parametric tests. However, the corresponding graphs

wrongly depict the data as mean +/- SD while they should depict median +/- IQR (or similar adequate value) because non-parametric statistical tests do not compare means but medians.
We thank the reviewer for spotting this, we will correct the graphs in Fig. S5A, B and S6A.
This mistake has now been corrected in Figure S5A, B (now EV2A-D), and Figure EV3B (formerly S6A) is normally distributed.

3. Statistical analysis: For ANOVA, the F and p values are not listed anywhere. The presented asterisks in the graphs are only for non-ANOVA or ANOVA post-hoc tests. This does not allow to judge statistical significance well and should be corrected.
Again, thanks for spotting this, we will include them.
These values are now shown for all relevant graphs either on the figure caption or Table 1.

4. Asymmetric vs Symmetric cell divisions: Representative images in Fig.2B suggest that IF may affect the plane of cell division for the Type-1 aNSCs. The plane of cell division is an indirect indicator of symmetric vs asymmetric (exhaustive vs maintaining) modes of cell division. Is it possible, IF influences this, especially during the first week of IF (see major comment 5)?
This is an interesting hypothesis. However, since we do not see any effects on aNSC maintenance, it is unlikely that IF produces any long-lasting effects on the mode of division of aNSCs. In general, we did not notice a difference in the plane of division of aNSCs between control and IF mice, although we did not systematically test for this (would require specific short EdU pulses to capture aNSCs in M-phase). In Figure 2B, the two stem cells shown in the control are unlikely to be the two daughter cells after the division of one aNSC, as one of them is positive and the other negative for Ki67. We only pointed to the second one to show a Ki67-negative aNSC. We will emphasize this in the figure legend.
This is now explained in the figure legend (Figure 2C).

5. Improved and more accurate citations: Some references are not properly formatted (e.g., "Dias", page 7, line 288). Some references are included in generalizing statements when they do not contain data to support such statements. For example, Kitamura et al., 2006 did not determine the number of new neurons (only BrdU+ cells) in the SGZ, yet this reference is included among sources supporting that IF "promote survival of newly born neurons" (page 2, line 60). Authors should be more careful how the cite the references.
Thanks for spotting these mistakes, we will correct them and check again all our references. As for the sentence where the Kitamura paper is cited, most of the other references also use only BrdU+ cells while concluding that IF enhances the survival of new neurons. We will change new neurons for new cells to reflect this, which we already bring up in the discussion (see also extended discussion in previous BioRxiv version).
We have checked our references and made the changes suggested above.

6. How do the authors explain that they observe 73-80% caloric restriction and yet the final body weight is not different between IF and control animals? Would it suggest that the selected IF protocol or selected diet are not appropriate (see major point 4)?

We also found this surprising and were expecting a change in overall activity in IF mice, which we did not observe. Many factors might play a role, like, as the reviewer suggests, changes in stress levels, which we will measure and show in the revised version.
We measured stress levels, now shown in Figure 1I and EVM. We also provide further evidence that our IF protocol does work, as it changes glucose and hydrobutyric acid levels in the serum of the mice, as expected (Figure 1G, H and EV1K,M). Lines 145-158.

7. Given that aNSCs rely more on de novo lipogenesis and fatty acids for their metabolism as shown by Knobloch et al., Nature 2013 and given the interesting changes in RER with the IF shown in this study, it would be interesting to see whether there are differences in Fasn expression in aNSCs between control and IF animals (see minor point 4).
This is an interesting suggestion but given that we see no effect on aNSCs, we find it's unlikely and unnecessary to test for Fasn expression differences in our IF protocol.
We did not test for Fasn expression differences for the reasons above.

8. Determining apoptosis in the SGZ by picnotic nuclei (Figure S6A) should be supplemented by determining the number and/or proportion of YFP+ cells positive for the Activated Caspase 3.
We previously found that counting picnotic nuclei is a more accurate and sensitive readout of cell death in the DG, as cells positive for caspase 3 are extremely rare due to the high efficiency of phagocytosis of apoptotic cells by microglia (see Urbán et al., 2016).
For the above reasons, we did not perform a staining for activated caspase 3, but now show images of the picnotic nuclei we quantified in Figure EV3A, B.

Reviewer #1 (Significance (Required)):
General assessment:
This study concludes that aNSCs do not respond to the intermittent fasting. This expands and supplements previous findings that suggest that the intermittent fasting promotes adult neurogenesis by increasing survival and/or proliferation in the Subgranural Zone. The study is well designed, however, over-extends its conclusions beyond a specific fasting paradigm and does not acknowledge serious limitations in the experimental design and analyses. In fact, until major revision is done, which would rule out that the absence of effects of fasting on aNSCs is not due to false negative results, many conclusions from this study cannot be accepted as valid.
Advance:
As mentioned above, the study has a potential to advance our understanding of how fasting affects neurogenesis and fills the knowledge gap of how fasting specifically affects the stem cells. However, unless the study addresses its limitations, its conclusions are not convincing.
Audience:
This study would be particularly interesting for the niche readers from the neurogenesis field. However, the study can also be interesting for researchers in metabolomics and dietology.
My expertise:
adult neurogenesis, neural stem cells, dietology, metabolism

Authors' Response

We disagree with the reviewer and find our conclusions well balanced, as we acknowledge our results are to be compared only with similar IF protocols. We also do not believe our results can be attributed to a false negative, as we consistently observe the same with different strains and protocols, always with sufficient animals to make our counts conclusive.
We nevertheless thank the reviewer for assessing our paper and for the advice to improve it. We hope that the reviewer will maintain the same level of scrutiny and scepticism with all IF-related papers.

Reviewer #2 (Evidence, reproducibility and clarity (Required)):
In this manuscript, Gabarro-Solanas et al. question the suitability of IF (Intermittent fasting - non-pharmacological strategy to counteract ageing, which has been previously shown to increase the number of adult-born neurons in the dentate gyrus of mice) as a pro-neurogenic intervention, since IF treatment did not stimulate adult hippocampal neurogenesis, neither at the stem cell level nor on immature and/or dividing neurons. The Authors used a tamoxifen inducible transgenic model (Glast-CreERT2;RYFP mice) to trace neural stem cell lineage and found that IF did not enhance neural stem cell proliferation, nor the abundance of immature, DCX+ neurons. Three-months of IF failed to increase the number of new adult-born neurons (NeuN+/YFP+), while one month of IF significantly reduced the number of new adult-born neurons.
The study appears technically sound, including many different approaches in order to reach its conclusions.
For instance, tamoxifen has been reported to impair various physiological processes, including neurogenesis (Smith et al., 2022), and most studies on adult hippocampal neurogenesis use the C57BL/6J strain of mice; hence, the use of Tamoxifen or that of the GlastCreERT2;RYFP model may have underscored these observations. However, to account for this potentially confounding factor, the Authors characterised the effect of their IF treatment in C57BL/6j mice, also reporting no evident effects of IF as a pro-neurogenic intervention.
I think the study was carefully planned and the analyses well done. Several possible variables were considered, including sex, labelling method, strain, tamoxifen usage or diet length. Several controls were performed in other organs and tissues (liver, fat) to establish the fasting protocol and to check its effects.
Data are presented in a clear way. Quality of images is high level.
In general, it appears as a highly reliable paper reaching an authoritative conclusion for the absence of effect of IF on adult neurogenesis.
Major comments:
I think that the key conclusions are convincing and no further experiments are required.
The methods are presented in such a way that they can be reproduced, and the experiments adequately replicated with proper statistical analysis.
We thank the reviewer for the encouraging remarks and the appreciation of our efforts.

Minor comments:
Prior studies are referenced appropriately, both regarding the IF protocols and the adult neurogenesis modulation.

Line 288 - a reference is incomplete (Dias); integrate with: (Dias et al., 2021)
We will re-format the reference, thanks for spotting the mistake.
This mistake has been fixed.

There is one concept that is not expressed in the manuscript. Maybe it is not strictly necessary, but I think can be useful to mention it here. It is the fact that most information currently available strongly indicates that adult neurogenesis in humans is not present after adolescence. Of course the research described here is carried out on mice, and in the manuscript it is stated many times that adult hippocampal neurogenesis is strongly decreasing with age, also due to age-related stem cell depletion. Yet, it seems that in humans the exhaustion of such a process can start after adolescence. We know that a sort of controversy is currently present on this subjects, because DCX+ neurons can be detected in adult and old human hippocampi. Yet, it is also clear that there is no substantial cell division (stem cells are depleted) to sustain such hypothetical neurogenesis. Hence, it has been hypothesized that non-newlyborn, "immature" neurons can persist in the absence of cell division, as it has been well demonstrated in the cerebral cortex (see La Rosa et al., 2020 Front Neurosci; Rotheneichner et al., 2018, Cereb Cortex).
This point can be important in the case someone want to use dietary approached such as IF (or any other pharmacological treatment) to stimulate neurogenesis in humans.
We agree with the reviewer and also find this a very interesting and timely topic. However, we find it a bit far from our results and would prefer not to comment on it in the context of the current paper.
We decided not to add a comparison to human adult neurogenesis in our manuscript.

Reviewer #2 (Significance (Required)):
The significance of this study relies on the fact that adult neurogenesis field (AN) has been often damaged by the search of "positive" results, aiming at showing that AN does occur "always and everywhere" and that most internal/external stimuli do increase it. This attitude created a bias in the field, persuading many scientists that a result in AN is worthy of publication (or of high impact factor publication) only when a positive result is found.
Personally, I found particularly meaninful the last sentences of the Discussion (reported below), which might seem "off topic" in a research paper, while - I think - underline the real significance of the manuscript:
"In addition, publication bias might be playing a role in skewing the literature on fasting and neurogenesis towards reporting positive results.
In some reviews, even studies reporting no effect are cited as evidence for improved neurogenesis upon IF. Reporting of negative results, especially those challenging accepted dogmas, and a careful and rigorous evaluation of the publications cited in reviews are crucial to avoid unnecessary waste of resources and to promote the advancement of science."
Reviewer field of expertise - keywords: adult neurogenesis, brain structural plasticity, non-newly born immature neurons, comparative neuroplasticity.

We are very happy that the reviewer shares our concern with the biased publication of positive results in the field. We hope our work (and that of Roberts et al., 2022) will encourage other labs to publish their negative results.

Reviewer #3 (Evidence, reproducibility and clarity (Required)):

In this manuscript, Gabarro-Solanas et al. investigate the effects of intermittent fasting (IF) on adult hippocampal neurogenesis in young adult mice. IF has been reported to increase the number of adult-born neuron in the hippocampus, a region that is important for learning and memory. However, it is not well understood what stages of adult neurogenesis are regulated by IF. To address this, the authors utilized lineage tracing and label retention assays in mice undergoing an IF diet. The authors used 2 months old Glast-CreERT2;RYFP mice in combination with Edu label retention to characterize adult NSCs and placed these mice on 1 and 3 months of IF. Despite seeing a decrease in neural stem cell proliferation with age, the authors did not observe a change due to diet. The authors then used immunohistochemistry to characterize changes in cell proliferation, neuroblasts, and new neurons following 1 month and 3 months of IF. Only 1 month of IF seemed to decrease the number of new neurons; however, by 3 months the neuronal output was the same. There were no differences in neuroblasts or cell proliferation due to diet. Gabarro-Solanas et al. conclude that IF transiently and mildly inhibits neurogenesis. Due to contradicting results, the authors then try to determine what variables (sex, labeling method, strain, tamoxifen usage, or diet length) could be affecting their data. The authors saw no substantial differences due to any of their variables.

Major Points

1. The authors analyze NSCs homeostasis and neurogenesis in young adult mice and do not observe any significant changes with their chosen alternate day intermittent fasting paradigm. However, a lot of the data and cell counts appears to be highly variable between animals in the same group. At times, there is an order of magnitude difference between the highest and lowest counts (e.g. Figure 2C,E). According to the method section, it appears that the authors predominantly analyzed a single DG (section?) for most immunostainings, which may explain the large variability in their data. If this is indeed the case, it is insufficient to quantify only a single section for each animal. The authors should quantify several DG sections for each mouse from a pre-defined range along the rostral-caudal axis of the hippocampus in accordance with a standard brain reference atlas. There are also several quantifications, especially of Ki67 where several individuals appear to have no Ki67+ (Figure 3B, 6D) NSCs. These findings are surprising given the still young age of these mice and may be another reflection of the limited brain sections that were analyzed.

The counts are indeed very variable. The counts were made on 1 to 4 DG sections (counted in full), depending on the staining. We will more clearly disclose this information in the revised version. In addition, we will re-count the neuronal output after fasting using stereology. Regarding the very low number of Ki67+ aNSCs, our counts are lower than those in other publications because we are much more stringent with our aNSC identification. Instead of using merely Sox2 (which also labels IPCs), we rely on the presence of a radial GFAP+ process.

We re-quantified neuronal production using stereological guidelines and we find now a small decrease in the number of EdU$^+$ cells upon IF. The data can be found in Figures 5G, 6G and EV4. See response to point 3 of reviewer #1 for more details.

2. There appear to be significant cutting or imaging artifacts across most fluorescent images further raising concerns regarding the accuracy of the quantifications (e.g. Figure 3D, 4C,E, 6B) and publication quality of the images and data. Importantly, uneven section thickness, either from cutting artifacts or imaging issues, may lead to inaccurate cell quantifications a could, possibly, account for the high variability. This issue would further exacerbate concerns regarding the quantification of a single DG section for each animal.
We only processed those samples that passed our QC after sectioning, meaning any unevenly cut brains were never considered (or stained). The stitched images do show artifacts (lower signal in the image junctions), particularly in the NeuN staining. However, this did not affect quantifications, as the measured levels were always clearly above the threshold to consider a cell positive, regardless of the position within the image. The images were cropped to improve the visualisation of NSCs, and to avoid the display of empty tiles. A low magnification image will be provided in the revised version to show that there were no staining artifacts.
We have re-taken the image in Figure 4E to show a more even distribution. As stated above, this stitching artifact did not alter our counts. We also show at the end of the response letter an uncropped image of the DG stained for GFAP and YFP.

3. It is unclear how NSCs were counted in the B6 mice (Fig 6D,E). The authors only provide a description for the Glast-CRE mice, where they used YFP labeling and GFAP. We assume they performed Sox2/GFAP or Nestin labeling, however, this is not clear at all. The authors should describe their methodology and provide representative images.
We used GFAP, location and morphology to count aNSCs in non-YFP mice. We will make this clear in the text and will also add one more count using Sox2, GFAP and Nestin to identify aNSCs.
We have re-counted NSCs in the B6 mice (as well as in the Glast-CRE mice) using GFAP, Sox2 and Nestin staining, nuclear localisation and the presence of a radial process. The data can be found in Figure EV2E-J.

4. NSC populations represent a heterogenous group of stem cells with different replicative properties. As such, the Glast-Cre approach used for the majority of this study may represent a specific subset of NSCs. In line with the previous point, we recommend the authors complement their NSC counts with Sox2/GFAP and Nestin immunostainings.
aNSCs labelled with Glast-Cre are the great majority of aNSCs (>90%) in both ad libitum fed and fasted mice. The data will be included in the revised version. Nevertheless, we will add counts using Sox2, GFAP and Nestin for key experiments.
As stated for point 3, the new counts using Sox2, GFAP and Nestin are now shown in Figure EV2E-J.

5. Stress is a significant negative regulator of neurogenesis. Is it possible that the IF mice display higher stress level which could counteract any beneficial effects of the IF intervention. The authors should provide some measures of stress markers to rule out this potential confounding factor in their IF paradigm.
This is a great suggestion. We will collect blood from control and fasted mice and measure the levels of stress factors (e.g. corticosterone). We will include the data in our revised version.
We found that corticosterone levels were not significantly different between control, and IF mice on either fasting or feeding days, however, we did see a trend to higher levels after both one cycle and 1 month of IF on fasting days that was restored upon refeeding. The data is now displayed in figures 1I and EV1M and lines 155-158.

Minor Point
1. The authors state that "Experimental groups were formed by randomly assigning mice from different litters within each mouse strain and all experiments were conducted in male and female mice". Given that neurogenesis, especially at young ages, is highly sensitive to the exact age of the mice, the authors should provide a rationale why animals from different litters instead of littermate controls were used in these experiments.
Littermate controls were always used in the experiments. But also, more than one litter was used for each experiment, since one litter was never generating enough mice for the experiments. We will clarify this point in text.
We have added this information to the material and methods section (lines 465-467).

2. Currently, the statistical tests are only described in the method section, however it would be helpful if this information to be integrated into the figure legend as well. Additionally, the authors provide individual data points for some but not all bar graphs (eg Figure 1D).
We will consider including the statistical information in the figure legend, provided there is not a maximum length for figure legends. In the case of figure 1D, data points are not shown because of how the food intake was calculated: as an average per cage instead of per animal (included in the materials and methods). We therefore do not consider it useful to show the datapoints in the final version of the manuscript but will provide them for the reviewer.
We now disclose all statistical information in the figure legends or Table 1. We also include a graph with individual data points for Figure 1D at the end of the revision plan.

3. Cell counts per AU is a rather unorthodox unit. With a representative selection of tissue for each animal, the authors could avoid the need to normalize to the DG length and may be able to extrapolate an estimate of cell counts for the entire DG instead.
Thanks for the suggestion. Our arbitrary units (AU) were in fact already equivalent to cells per mm of DG, and we have updated our graphs to reflect this.
The graphs have been updated accordingly.

4. In Figure 4D, the authors highlight a few NSC with arrowheads. At a quick glance this is rather confusing as it appears that the authors only counted 3 NSCs in each picture. It may be a better option to show a zoomed in picture to highlight an example of a representative NSC.

Examples of representative NSCs are already shown in Fig 2. With this image, we intended to show a larger number of NSCs. We realise the arrows only pointed to some of them, making the message confusing. We will consider removing them from the figure in the revised version.
We have removed the arrows in the figure.

5. In Supplementary Figure S6, the authors should complement the quantification of the nuclei with representative images.
We will include representative images in Figure S6.
The images are now shown in Figure EV3.

6. For the daytime IF, did the authors assess weights, food intake, RER as well liver/fat measurements similar to night-time IF? If so, this data should be provided in the supplement.
We do have data for the daytime IF in the metabolic cages, which was taken from mice housed in groups (during the preliminary phase of our study). We also have the weight and data on neurogenesis, which we will show as a supplement.
We found including the data in the main manuscript a bit distracting from our main message, but we show it at the end of the response letter.

Reviewer #3 (Significance (Required)):
The authors are commended for compiling a manuscript on what is commonly considered 'negative data', that, at the same time, are also contradicting independent reports on the effects of IF on neurogenesis. The studies outlined in this manuscript are comprehensive and mostly well designed. Given the broad, growing interest in dietary restriction as an aging intervention the study is timely.
We thank the reviewer for the positive assessment of the significance of our work.

Reviewer #4 (Evidence, reproducibility and clarity (Required)):
Summary:
In this manuscript, Gabarró-Solanas et al. tested the effect of intermitted fasting (IF, every-other-day fasting) on adult neural stem cells and neurogenesis. They demonstrate that the paradigm they have used does not affect NSC activation or maintenance, and does also not promote neurogenesis. As previous reports showed increased neurogenesis with IF, the authors controlled for various parameters such as mouse strain, sex, and diet length. They also used different methods of identification of newborn neurons, such as tamoxifen-induced lineage-tracing versus birth-dating with thymidine-analogues to substantiate their findings.
Major comments:
This study is very well done with carefully designed and controlled experiments. The manuscript reads nicely and the data are presented in a clear way, making it easy to follow. The authors have done a "tour-de force" to rule out confounding factors that might influence their findings that IF does not affect NSCs nor neurogenesis.
The claims and conclusions are supported by the data. The methods are clearly described and should allow to reproduce the data independently. The number of replicates (i.e. the number of mice analyzed) is impressive and statistical analysis is adequate.

The major findings, namely that the chosen IF does not affect NSCs and neurogenesis is not in line with some previous studies. Despite a careful ruling out of potentially confounding factors (see also "significance" below), it remains unclear why other studies have found an increase in neurogenesis with IF. As each of these studies has some specific experimental design, it is difficult to judge these data in the context of previous data without going through all the details of the other studies. It would thus be a great help for the reader if the authors could provide a table or schematic, which lists the major parameters of each of these studies, such as detailed paradigm of IF, age of mice at start, sex, duration of the intervention, method of identification of NSCs and neurogenesis etc.

This is a very good suggestion, and we had already created such a table. We, however, consider that it might be better suited for a review on the effects of IF on neurogenesis than for this work. We will include the table in our response to the reviewers together with our revised version.

A table summarising some of the most relevant IF and caloric restriction papers can be found as a supplementary file in our resubmission (R_IFpapers).

Two points that the authors have not discussed might also be worth mentioning in the discussion part:

1.) The mice in the night-time IF were single caged, could there be a potential negative effect on neurogenesis that would mask the presumably beneficial effect of IF? Although the controls were also single caged, the stress of social isolation might play a role?

The mice were only single caged for the metabolic phenotyping, but not for the neurogenic counts. We will make this clearer in the text. In any case, we do agree that stress might play a role and we will measure stress levels in the control and fasted mice and will include this data in the revised version.

We have more clearly indicated the caging conditions for different experiments in the material and methods (lines 465-468). We now also include measurements of stress levels after one cycle and one month of IF, show in Figure EV1M and 1I (lines 155-158).

2.) The IF mice gained the same weight over time (Fig. S2), but had a ~20% reduction in overall calory intake. This would be explainable by a reduction in energy expenditure, but the overall activity was also not significantly changed (Fig. S1). Can the authors speculate why they reach the same weight with less calories?

We also found this surprising and were expecting a reduction in the overall activity of the fasted mice. We do not have an explanation for this discrepancy, but perhaps stress levels might explain part of it (we will check stress levels in the revised version). We will also look at whether energy expenditure and activity levels changed over time.

Our measurements show no long-lasting increase in corticosterone levels in IF mice (Figure 1I, lines 155-158). We also checked daily activity and energy expenditure over time and saw no obvious differences between control and fasted mice (Figure EV1E, F, lines 118-119).

Minor comments:

Authors' Response

1.) It would be nice to replace the arbitrary units (AU) in the graphs were this is used (e.g. Fig. 2F, 3C, 4B, D and F etc) to the actual number of cells per a certain µm DG, so that the number of cells can be put in context and compared between the figures.
Yes, our AU already corresponded to mm and we will update our figures accordingly.
Figures have been updated.

2.) Fig 3 D: can the authors also show the Ki67 channel to illustrate how it looks after a 3 month IF?
We find it does not help much, as Ki67+ cells are mostly IPCs and that data is already shown in Fig. 4A. We will nevertheless include the image in our response to the reviewers together with our revised version.
The image is shown at the end of the response letter.

3.) Fig.4E: the NeuN staining looks strangely interrupted, this might be due to tile-stitching? In that case, it would be better to either only show one segment or to try to get a better stitching algorhythm.
It is indeed because of the tile-stitching and uneven illumination. However, this did not affect the counts, as already discussed in the response to reviewer #3 (major point #2).
We replaced the image in Figure 4E to show that the staining was uniform.

4.) Fig.6 D shows a minus axis in Y-axis, this should only been shown from 0 to positive values, as it is a percentage of cells and cannot be negative.
True, thanks for spotting this. We will correct the graphs in the revised version.
The mistake has been corrected.

5.) Fig.6 B: the same problem with the NeuN staining as mentioned under point 3. This should be improved.
As with point 3, the stitching did not affect the quantification. We find it more accurate to show the image with the stitching, as that was the one used for quantification. We will provide a new picture with lower magnification to better show the quality of the staining.
A new image is now provided.

6.) Fig. S6B: maybe add a comment in the result part or in the figure legend that a 10 day chase after an EdU pulse is not the classical protocol to look at mature NeuN positive neurons. But apparently enough newborn neurons were already NeuN positive for this quantification.
We fully agree 10 days is not the standard for neuronal identification. We did find neurons after the 10-day chase but in low numbers. We will add a comment in the text of the revised version to clarify this.
We now clarify this in the figure legend in Figure EV3.

7.) The authors refer to personal communications with M. Mattson and S. Thuret to underline that circadian disruption is not enough to explain the differences (line 367 onwards). Can they refer the reader to published data instead?

While the results are published in their papers, the methods did not specify the time at which the food was added/removed for the IF protocol. That is why we refer to personal communication. Further showing that disruption of circadian rhythms is not enough to explain the difference in outcome of the IF protocol, we will show the data for the 1-month daytime IF, which again does not increase adult neurogenesis (reviewer #3, minor point #6).
We found this information did not add much to our conclusions but the results can be found at the end of the response letter.

Reviewer #4 (Significance (Required)):
Given the great interest in the seemingly positive effects on health of IF in general, and also for increasing neurogenesis, it is important to better understand the mechanism of this intervention. The study by Gabarró-Solanas et al. clearly demonstrates that IF is not a universal, "works all the time" way of increasing neurogenesis. The study is very well done, with well controlled and measured parameters. It shows that a physiological interference such as IF might depend on many factors and might be less robust across laboratories than anticipated. This study is a very good example that all the details of the experimental settings need to be taken into consideration and are ideally reported with every IF study. It is also a good example how to follow up "no effect" data in a way that they are conclusive.
The significance of this study is to point out that IF as a strategy to increase neurogenesis needs to be reconsidered. It raises the questions how IF can be beneficial in some studies and not in others, asking for more experiments to better understand the detailed mechanisms of IF action. In a systematic approach, this study rules out some of the potentially confounding factors and shows that at least with the chosen IF paradigm, these factors are not the reason for not seeing increased neurogenesis. The study is thus of clear interest for the neurogenesis field and will also need to be considered by the broader field of IF research, although it speaks against the beneficial effects of IF. It might have the potential to bring together the different study authors who did or did not see increased neurogenesis with IF and discuss together the non-published details of their study design to advance the field.
We thank the reviewer for the positive assessment of our work and for acknowledging its importance for the broader field of IF research.

Figures

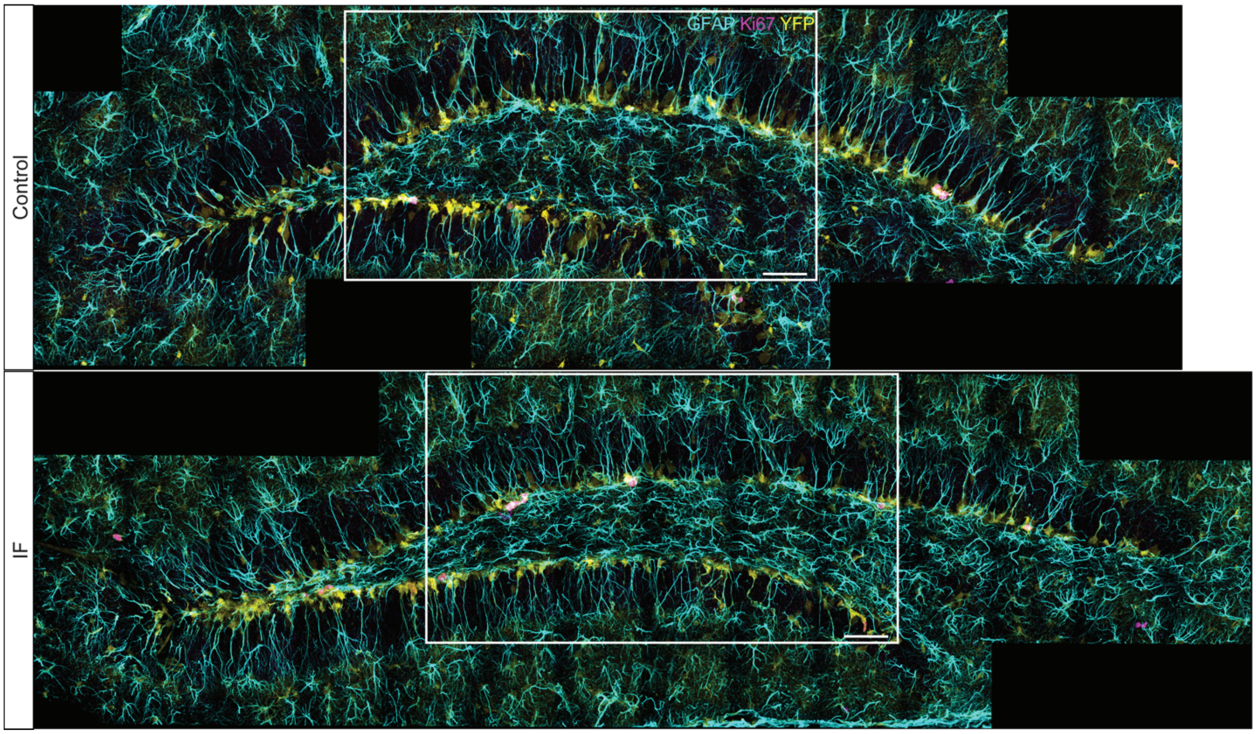

Figure 1: Uncropped images of NSCs in the DG with Ki67. Scale bar: 50μm. White rectangle indicates image area displayed in **Figure 3**.

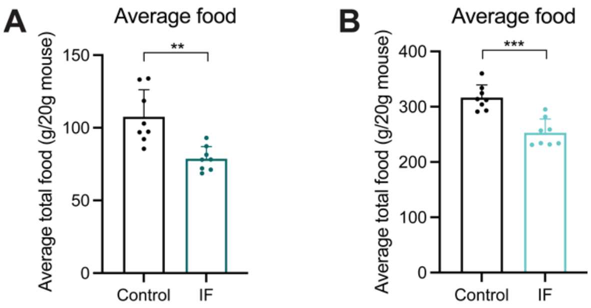

Figure 2: Graphs displaying individual data points for **Figure 1D** (**A**) and **Figure EV1M** (**B**). Bars and error bars represent average + s.d.; dots represent individual cages where mice were housed in groups.

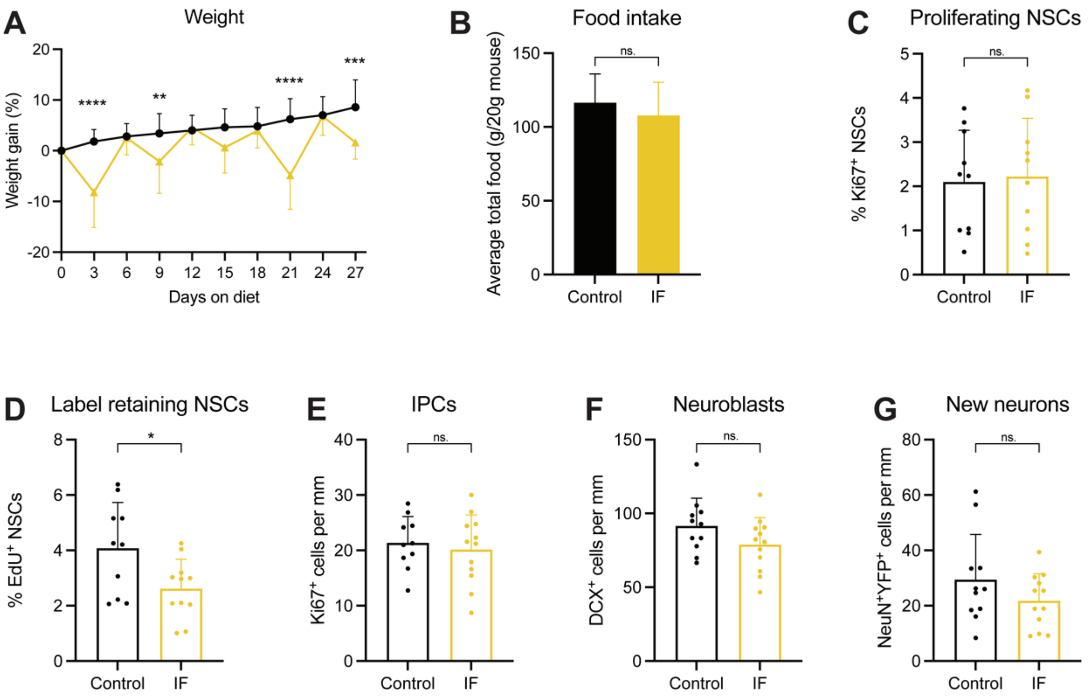

Figure 3: Effects of 1-month long daytime IF on adult neurogenesis in the DG.

(**A**) Mouse weight every 3 days shown as the percentage of weight difference to the first day of the diet. Days 3, 9, 15, 21 and 27 show weight on fasting days, and days 6, 12, 18 and 24 on feeding days.

(**B**) Average total food consumed for a month shown as g of food per 20 g of mouse weight (weight reference of mice at the beginning of the diet).

(**C**, **D**) quantification of the percentage of proliferating ($Ki67^+$, **C**) and label retaining (EdU^+, **D**) NSCs.

(**E**, **F**, **G**) Quantification of the number of IPCs (**E**), neuroblasts (**F**) and new neurons (**G**) normalised to DG length.

Bars and error bars represent average + s.d.; dots represent individual mice.

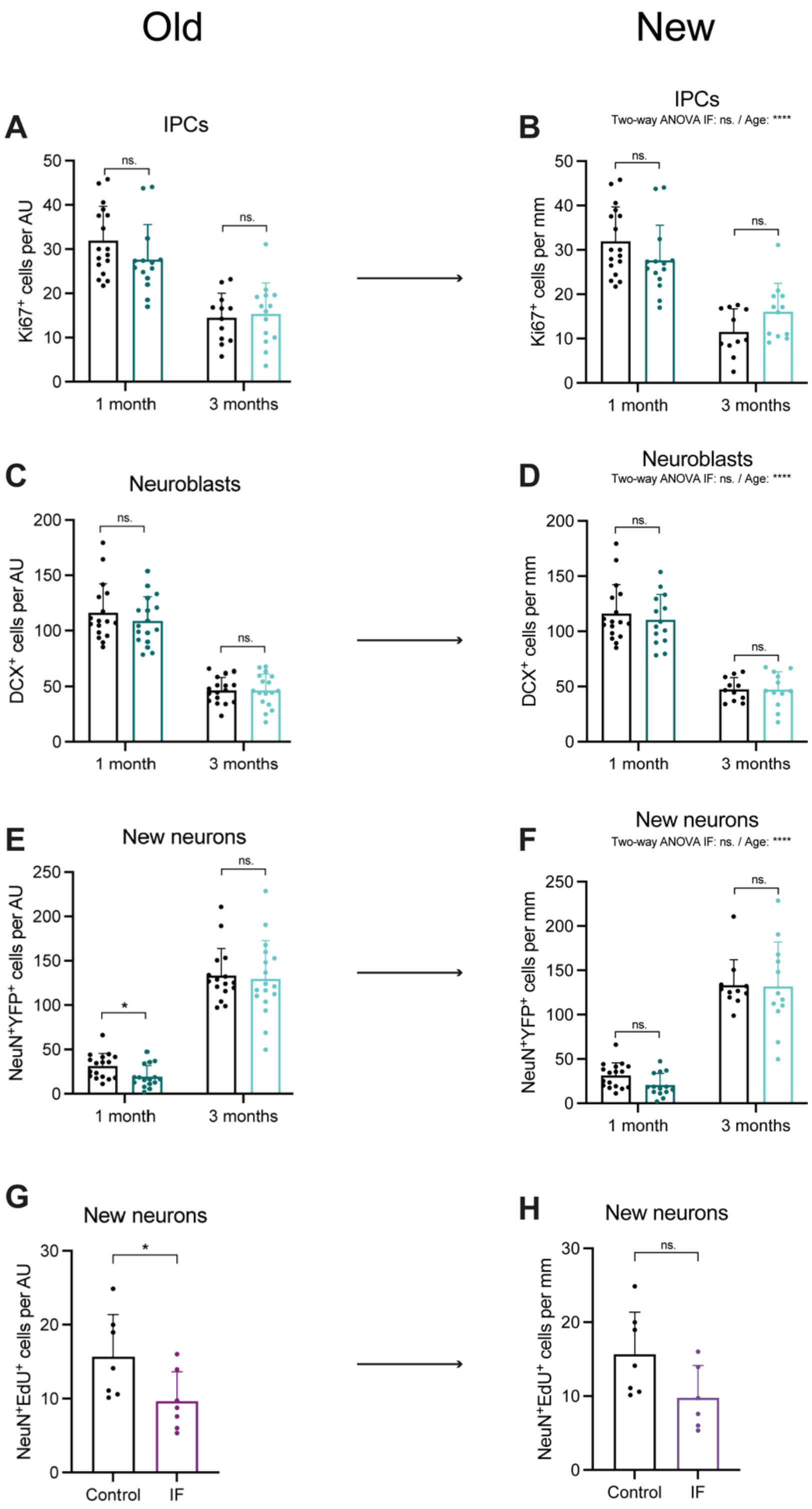

Figure 4: Original and new graphs (with same Ns in each graph) shown side by side.
(**A-F**) Graphs from Figure 4 sowing that neurogenesis does not increase upon IF. The significance change from E to F is due to the change in statistical test from t-test to Two-way ANOVA to take age into account.

(**G**, **H**) Graphs from Figure 6, where the p-value increases above 0.05 after excluding Ns that could not be counted in another staining.

3. Description of the revisions that have already been incorporated in the transferred manuscript

Please insert a point-by-point reply describing the revisions that were already carried out and included in the transferred manuscript. If no revisions have been carried out yet, please leave this section empty.

4. Description of analyses that authors prefer not to carry out

Please include a point-by-point response explaining why some of the requested data or additional analyses might not be necessary or cannot be provided within the scope of a revision. This can be due to time or resource limitations or in case of disagreement about the necessity of such additional data given the scope of the study. Please leave empty if not applicable.

Please, find more detailed reasons in the main response to each reviewer.

- Behavioural tests such as open field or elevated T maze to assess stress and anxiety levels: we think that a more accurate way of assessing stress would be through the analysis of stress hormone levels in blood. This alternative measurement will be included in the revised version of the manuscript.
- Analysis of parameters such as astrocyte reactivity, inflammation or microglia activation to evaluate whether the used IF protocol elicits cellular or molecular changes in the CNS: after revising the literature, we found no evidence that IF alone influences any of those parameters.
- Changing the title: we think that our title and abstract faithfully reflect our data and conclusions.
- Assessing potential effects on symmetric vs. asymmetric NSC divisions: the images in Fig 2B cannot be taken as evidence of a change in plane of cell division upon IF. Additionally, there are no effects on NSC maintenance which would be expected after a change in the mode of cell division, so we consider this analysis unnecessary.
- Quantifying differences in Fasn expression in NSCs from control and IF animals: we think there is no need to test for Fasn expression since there is no effect on NSCs.
- Determining apoptosis by activated Caspase 3 staining: we have seen in previous work that picnotic nuclei reflect cell death in the DG more accurately (see Urbán et al., 2016).
- Discussing human neurogenesis: we find this is out of the scope of our paper.
- A table summarising previous publications: we think that this would be more suited for a review article.
- Including the Ki67 channel in Fig. 3D: we think it does not help the visualisation of the NSCs.

List of references used in the response to reviewers:

Anson, R. M. *et al.* Intermittent fasting dissociates beneficial effects of dietary restriction on glucose metabolism and neuronal resistance to injury from calorie intake. *Proceedings of the National Academy of Sciences* **100**, 6216–6220 (2003).

Bok, E. *et al.* Dietary Restriction and Neuroinflammation: A Potential Mechanistic Link. *International Journal of Molecular Sciences* **20**, 464 (2019).

Cignarella, F. *et al.* Intermittent Fasting Confers Protection in CNS Autoimmunity by Altering the Gut Microbiota. *Cell Metabolism* **27**, 1222-1235.e6 (2018).

Dai, S. *et al.* Intermittent fasting reduces neuroinflammation in intracerebral hemorrhage through the Sirt3/Nrf2/HO-1 pathway. *Journal of Neuroinflammation* **19**, 122 (2022).

Dias, G. P. *et al.* Intermittent fasting enhances long-term memory consolidation, adult hippocampal neurogenesis, and expression of longevity gene Klotho. *Mol Psychiatry* 1–15 (2021).

Goodrick, C. L., Ingram, D. K., Reynolds, M. A., Freeman, J. R. & Cider, N. Effects of intermittent feeding upon body weight and lifespan in inbred mice: interaction of genotype and age. *Mechanisms of Ageing and Development* **55**, 69–87 (1990).

Gudden, J., Arias Vasquez, A. & Bloemendaal, M. The Effects of Intermittent Fasting on Brain and Cognitive Function. *Nutrients* **13**, 3166 (2021).

Lee, J., Seroogy, K. B. & Mattson, M. P. Dietary restriction enhances neurotrophin expression and neurogenesis in the hippocampus of adult mice. *Journal of Neurochemistry* **80**, 539–547 (2002).

Rangan, P. *et al.* Fasting-mimicking diet cycles reduce neuroinflammation to attenuate cognitive decline in Alzheimer's models. *Cell Reports* **40**, 111417 (2022).

Roberts, L. D. *et al.* The 5:2 diet does not increase adult hippocampal neurogenesis or enhance spatial memory in mice. 2022.10.03.510613 BioRxiv Preprint (2022).

Song, M.-Y. *et al.* Energy restriction induced SIRT6 inhibits microglia activation and promotes angiogenesis in cerebral ischemia via transcriptional inhibition of TXNIP. *Cell Death Dis* **13**, 449 (2022).

Urbán, N. *et al.* Return to quiescence of mouse neural stem cells by degradation of a proactivation protein. *Science* **353**, 292–295 (2016).

1st Revision - Editorial Decision — 30th Aug 2023

Dear Dr. Urbán Avellaneda,

Thank you for submitting your revised manuscript to EMBO reports and for your patience during its peer review. I apologize for the slow response, which was due to the limited availability of the referees during the summer holidays period. We have now received the full set of reports from the three referees that were asked to re-evaluate your study. Please find their comments included below.

As you will see, all three referees are satisfied with the revision, they explain that the study and the manuscript have been substantially improved, and the majority of their previous concerns satisfactorily addressed, and they now all recommend publication of the manuscript in EMBO reports. There are only three suggestions for minor improvements in Figures from referee #3, which we would like you to address in a revised version of your manuscript before we can proceed with its acceptance.

From the editorial side, there are also a few things that we need from you:

- You can increase the number of keywords up to 5, if you wish.

- Please note that a data availability statement (at the end of Materials and Methods) is mandatory. If your study does not include any datasets requiring deposition in a public database, please add the statement: "This study includes no data deposited in external repositories." under the heading "Data availability" at the end of Materials and Methods. Please also update your Author checklist accordingly.

- Please update your conflict-of-interest statement: the heading should be "Disclosure and competing interests statement".

- The author contributions statement should be removed from the manuscript file. Instead, we now use CRediT to specify the contributions of each author in the journal submission system. Please use the free text box to provide more detailed descriptions. See also our guide to authors:
.

- Please remove the bold text formatting from your references list.

- We noticed that the funding information regarding the Institute of Molecular Biotechnology of the Austrian Academy of Science is missing from our online manuscript handling system. Please provide the full funding information in the system and make sure that it matches that included in the Acknowledgements section of the manuscript.

- Callouts for Fig. EV3 are missing; please make sure that all Figure panels are called out (in alphabetical order) in your revised manuscript.

- Please add the heading "Expanded View Figure Legends".

- In checking your Figures, we noticed possible re-use of images. Please note that re-use must be mentioned in (both) figure legends. Re-use is indeed mentioned for Appendix Figure S1B and Figure 1K (which is fine), but not for Appendix Figure 1A and Figure 1J. Please check the possible re-use and mention this clearly in the respective figure legends.

- Your Figure legends have been inspected by our data editors for completeness and accuracy. Please see the required changes in the attached Word file and address all comments in your revised manuscript (with tracked changes).

- Please remove the Appendix legends from the main manuscript file. They should be included in the Appendix separate pdf file (please see the next point below).

- If Table 1 does not fit into an A4 page, it should be provided as an Expanded View (EV) Table. Please see our guide for authors for more information: https://www.embopress.org/page/journal/14693178/authorguide#expandedview

- The Appendix should be uploaded as a single pdf file (the 2 figures are now uploaded separately) starting with a brief Table of Contents including page numbers on its first page. The names of the figures should be "Appendix Figure S#"; please also correct the in-text callouts accordingly.

- The manuscript sections are in the wrong order. Please follow the order: Title page, Abstract, Keywords, Introduction, Results, Discussion, Materials and Methods, Data availability, Acknowledgements, Disclosure and competing interests statement, References, Figure legends, Expanded View Figure legends.

- Please note that EMBO press papers are accompanied online by:
A) a short (1-2 sentences) summary of the findings and their significance,

B) 2-4 short bullet points highlighting the key results, and
C) a synopsis image that is exactly 550 pixels wide and 300-600 pixels high (the height is variable). You can either show a model or key data in the synopsis image. Please note that the text needs to be readable at the final size.
Please upload this information along with your revised manuscript (the text for A and B should be provided in a separate Word file).

Please also note that as part of the EMBO publications' Transparent Editorial Process, EMBO reports publishes online a Review Process File to accompany accepted manuscripts. This File will be published in conjunction with your paper and will include the referee reports, your point-by-point response and all pertinent correspondence relating to the manuscript.

You can opt out of this by letting the editorial office know (emboreports@embo.org). If you do opt out, the Review Process File link will point to the following statement: "No Review Process File is available with this article, as the authors have chosen not to make the review process public in this case."

We would also welcome the submission of cover suggestions or motifs to be used by our Graphics Illustrator in designing a cover.

We look forward to seeing a final version of your manuscript as soon as possible.

Yours sincerely,

Ioannis Papaioannou, PhD
Editor
EMBO reports

Referee #1:

In this manuscript, entitled "Adult neural stem cells and neurogenesis are resilient to intermittent fasting", submitted to EMBO reports, Gabarrò-Solanas et al. question the suitability of IF (Intermittent fasting - non-pharmacological strategy to counteract ageing, which has been previously shown to increase the number of adult-born neurons in the dentate gyrus of mice) as a pro-neurogenic intervention, since IF treatment did not stimulate adult hippocampal neurogenesis, neither at the stem cell level nor on immature and/or dividing neurons. The Authors used a tamoxifen inducible transgenic model (Glast-CreERT2;RYFP mice) to trace neural stem cell lineage and found that IF did not enhance neural stem cell proliferation, nor the abundance of immature, DCX+ neurons. Three-months of IF failed to increase the number of new adult-born neurons (NeuN+/YFP+), while one month of IF significantly reduced the number of new adult-born neurons.
The study apperas technically sound, including many different approaches in order to reach its conclusions.
For instance, tamoxifen has been reported to impair various physiological processes, including neurogenesis (Smith et al., 2022), and most studies on adult hippocampal neurogenesis use the C57BL/6J strain of mice; hence, the use of Tamoxifen or that of the GlastCreERT2;RYFP model may have underscored these observations. However, to account for this potentially confounding factor, the Authors characterised the effect of their IF treatment in C57BL/6j mice, also reporting no evident effects of IF as a pro-neurogenic intervention.
I think the study was carefully planned and the analyses well done. Several possible variables were considered, including sex, labelling method, strain, tamoxifen usage or diet length. Several controls were performed in other organs and tissues (liver, fat) to establish the fasting protocol and to check its effects.
Data are presented in a clear way. Quality of images is high level.
In general, it appears as a highly reliable paper reaching an authoritative conclusion for the absence of effect of IF on adult neurogenesis.

I extensively reviewed a previous version of this manuscript in a preprint form. I gave a substantially positive evaluation (reported above) and asked for some changes/integrations. Here, in the new version, I see that most of the requests (including those made by other Reviewers), were taken into consideration and substantial changes have been made throughout the text. The manuscript is substantially improved in its content, strength, and clarity.

On these bases I think that is now suitable for publication in EMBO reports.

Referee #2:

In their revision, Gabarro-Solanas et al. significantly improved their manuscript. They addressed majority of my concerns by additional experiments, analyses, or appropriate statistical tests. Particularly, upon my suggestions, they monitored the stress of mice undergoing the intermittent fasting, analyzed selected data sets by stereological principles, added a post-fasting refeeding period and confirmed that their fasting protocol elicits expected metabolic changes. These experiments refined and improved interpretation of their data. On the other hand, they did not sufficiently address whether the fasting protocol changes quiescence of neural stem cells (by additional EdU/BrdU protocols) or their metabolic state (by determining Fasn activity or expression). Nevertheless, their revision produced more convincing and better-controlled manuscript that will substantially contribute to the discussion about the role of fasting and metabolism in neurogenesis. Because their robust data contradict some recently published results that suggest intermittent fasting promotes neurogenesis, their paper has a potential to stimulate further research in the field. Therefore, I am pleased to suggest the revised manuscript for publication in EMBO Reports without any additional revision or changes.

Referee #3:

In this revised version of the manuscript, the authors continue to explore the effects of intermittent fasting on hippocampus neurogenesis in young mice. The authors have adequately addressed previously raised concerns regarding NSC quantification methodology, image quality, and stress as a confounding factor. A few minor points are listed for the author to consider:

Minor.
1. In Figure 4E, include the arrow legend to specify what cell types the arrows are pointing to similar to Figure 2C.
2. In Figure 6B, in the representative image use arrows to point out Edu+ positive cells for quantification.
3. In Figure EV4, include representative images for quantification of neurogenesis in male and female mice.

***
Rev_Com_number: RC-2022-01713
New_manu_number: EMBOR-2023-57268V2
Corr_author: Urbán Avellaneda
Title: Adult neural stem cells and neurogenesis are resilient to intermittent fasting

From the editorial side, there are also a few things that we need from you:

- You can increase the number of keywords up to 5, if you wish.
We have now 5 keywords, as we added IF and NSCs.

- Please note that a data availability statement (at the end of Materials and Methods) is mandatory. If your study does not include any datasets requiring deposition in a public database, please add the statement: "This study includes no data deposited in external repositories." under the heading "Data availability" at the end of Materials and Methods. Please also update your Author checklist accordingly.
We added the data availability statement

- Please update your conflict-of-interest statement: the heading should be "Disclosure and competing interests statement".
It's updated

- The author contributions statement should be removed from the manuscript file. Instead, we now use CRediT to specify the contributions of each author in the journal submission system. Please use the free text box to provide more detailed descriptions. See also our guide to authors: https://www.embopress.org/page/journal/14693178/authorguide#authorshipguidelines.
We removed the author contributions from the manuscript text. We had already indicated the contributions through CRediT when submitting the paper.

- Please remove the bold text formatting from your references list.
Done

- We noticed that the funding information regarding the Institute of Molecular Biotechnology of the Austrian Academy of Science is missing from our online manuscript handling system. Please provide the full funding information in the system and make sure that it matches that included in the Acknowledgements section of the manuscript.
Will do when uploading. Institute of Molecular Biotechnology is not necessary, as the agency supporting the lab is the Austrian Academy of Sciences directly.

- Callouts for Fig. EV3 are missing; please make sure that all Figure panels are called out (in alphabetical order) in your revised manuscript.
We double checked this.

- Please add the heading "Expanded View Figure Legends".
Done.

- In checking your Figures, we noticed possible re-use of images. Please note that re-use must be mentioned in (both) figure legends. Re-use is indeed mentioned for Appendix Figure S1B and Figure 1K (which is fine), but not for Appendix Figure 1A and Figure 1J. Please check the possible re-use and mention this clearly in the respective figure legends.
We now mention the re-use of the figures in all cases.

- Your Figure legends have been inspected by our data editors for completeness and accuracy. Please see the required changes in the attached Word file and address all comments in your revised manuscript (with tracked changes).

- Please remove the Appendix legends from the main manuscript file. They should be included in the

Appendix separate pdf file (please see the next point below).
They are now in the appendix.

- If Table 1 does not fit into an A4 page, it should be provided as an Expanded View (EV) Table. Please see our guide for authors for more information:
https://www.embopress.org/page/journal/14693178/authorguide#expandedview
We re-arranged the tables and Table 1 (with the statistics) was transferred to the Appendix, while Table 2 became Table EV1.

- The Appendix should be uploaded as a single pdf file (the 2 figures are now uploaded separately) starting with a brief Table of Contents including page numbers on its first page. The names of the figures should be "Appendix Figure S#"; please also correct the in-text callouts accordingly.
Done.

- The manuscript sections are in the wrong order. Please follow the order: Title page, Abstract, Keywords, Introduction, Results, Discussion, Materials and Methods, Data availability, Acknowledgements, Disclosure and competing interests statement, References, Figure legends, Expanded View Figure legends.
We re-organised the sections.

- Please note that EMBO press papers are accompanied online by:
A) a short (1-2 sentences) summary of the findings and their significance,
B) 2-4 short bullet points highlighting the key results, and
C) a synopsis image that is exactly 550 pixels wide and 300-600 pixels high (the height is variable). You can either show a model or key data in the synopsis image. Please note that the text needs to be readable at the final size.
Please upload this information along with your revised manuscript (the text for A and B should be provided in a separate Word file).
We have prepared A, B and C.

We have also addressed reviewer 3's minor concerns:
1. In Figure 4E, include the arrow legend to specify what cell types the arrows are pointing to similar to Figure 2C.
Figure 4E has been updated accordingly. Also, we made the arrows in 2C bigger.

2. In Figure 6B, in the representative image use arrows to point out Edu+ positive cells for quantification.
Figure 6B has been updated to include the arrows.

3. In Figure EV4, include representative images for quantification of neurogenesis in male and female mice.
We have added appendix figure S3 showing representative images for the neurogenesis quantification (NeuN+YFP+) for both males and females.

2nd Revision - Editorial Decision 30th Oct 2023

Dear Dr. Urbán Avellaneda,

Thank you for submitting your revised manuscript. My colleague Ioannis has moved over to The EMBO Journal, I have thus stepped in as the handling editor of your manuscript. I have now looked at everything and all is fine. Therefore, I am very pleased to accept your manuscript for publication in EMBO Reports.

Congratulations on a nice work!

Kind regards,

Deniz Senyilmaz Tiebe
--
Deniz Senyilmaz Tiebe, PhD
Editor
EMBO Reports

At the end of this email I include important information about how to proceed. Please ensure that you take the time to read the information and complete and return the necessary forms to allow us to publish your manuscript as quickly as possible.

As part of the EMBO publication's Transparent Editorial Process, EMBO reports publishes online a Review Process File to accompany accepted manuscripts. As you are aware, this File will be published in conjunction with your paper and will include the referee reports, your point-by-point response and all pertinent correspondence relating to the manuscript.

If you do NOT want this File to be published, please inform the editorial office within 2 days, if you have not done so already, otherwise the File will be published by default [contact: emboreports@embo.org]. If you do opt out, the Review Process File link will point to the following statement: "No Review Process File is available with this article, as the authors have chosen not to make the review process public in this case."

Should you be planning a Press Release on your article, please get in contact with emboreports@wiley.com as early as possible, in order to coordinate publication and release dates.

Thank you again for your contribution to EMBO reports and congratulations on a successful publication. Please consider us again in the future for your most exciting work.

****

THINGS TO DO NOW:

You will receive proofs by e-mail approximately 2-3 weeks after all relevant files have been sent to our Production Office; you should return your corrections within 2 days of receiving the proofs.

EMBO Press participates in many Publish and Read agreements that allow authors to publish Open Access with reduced/no publication charges. Check your eligibility: https://authorservices.wiley.com/author-resources/Journal-Authors/open-access/affiliation-policies-payments/index.html

Please inform us if there is likely to be any difficulty in reaching you at the above address at that time. Failure to meet our deadlines may result in a delay of publication, or publication without your corrections.

All further communications concerning your paper should quote reference number EMBOR-2023-57268V3 and be addressed to emboreports@wiley.com.

Should you be planning a Press Release on your article, please get in contact with emboreports@wiley.com as early as possible, in order to coordinate publication and release dates.

***
Rev_Com_number: RC-2022-01713
New_manu_number: EMBOR-2023-57268V3
Corr_author: Urbán Avellaneda
Title: Adult neural stem cells and neurogenesis are resilient to intermittent fasting

EMBO Press Author Checklist

Corresponding Author Name: Noelia Urbán Avellaneda	
Journal Submitted to: EMBO reports	
Manuscript Number: EMBOR-2023-57268V1	

USEFUL LINKS FOR COMPLETING THIS FORM
The EMBO Journal - Author Guidelines
EMBO Reports - Author Guidelines
Molecular Systems Biology - Author Guidelines
EMBO Molecular Medicine - Author Guidelines

Reporting Checklist for Life Science Articles (updated January
This checklist is adapted from Materials Design Analysis Reporting (MDAR) Checklist for Authors. MDAR establishes a minimum set of requirements in transparent reporting in the life sciences (see Statement of Task: 10.31222/osf.io/9sm4x). Please follow the journal's guidelines in preparing your
Please note that a copy of this checklist will be published alongside your article.

Abridged guidelines for figures
1. Data
The data shown in figures should satisfy the following conditions:
- the data were obtained and processed according to the field's best practice and are presented to reflect the results of the experiments in an accurate and unbiased manner.
- ideally, figure panels should include only measurements that are directly comparable to each other and obtained with the same assay.
- plots include clearly labeled error bars for independent experiments and sample sizes. Unless justified, error bars should not be shown for technical
- if n<5, the individual data points from each experiment should be plotted. Any statistical test employed should be justified.
- Source Data should be included to report the data underlying figures according to the guidelines set out in the authorship guidelines on Data

2. Captions
Each figure caption should contain the following information, for each panel where they are relevant:
- a specification of the experimental system investigated (eg cell line, species name).
- the assay(s) and method(s) used to carry out the reported observations and measurements.
- an explicit mention of the biological and chemical entity(ies) that are being measured.
- an explicit mention of the biological and chemical entity(ies) that are altered/varied/perturbed in a controlled manner.
- the exact sample size (n) for each experimental group/condition, given as a number, not a range;
- a description of the sample collection allowing the reader to understand whether the samples represent technical or biological replicates (including how many animals, litters, cultures, etc.).
- a statement of how many times the experiment shown was independently replicated in the laboratory.
- definitions of statistical methods and measures:
  - common tests, such as t-test (please specify whether paired vs. unpaired), simple χ2 tests, Wilcoxon and Mann-Whitney tests, can be unambiguously identified by name only, but more complex techniques should be described in the methods section;
  - are tests one-sided or two-sided?
  - are there adjustments for multiple comparisons?
  - exact statistical test results, e.g., P values = x but not P values < x;
  - definition of 'center values' as median or average;
  - definition of error bars as s.d. or s.e.m.

Please complete ALL of the questions below.
Select "Not Applicable" only when the requested information is not relevant for your study.

Materials

Newly Created Materials	Information included in the manuscript?	In which section is the information available? (Reagents and Tools Table, Materials and Methods, Figures, Data Availability Section)
New materials and reagents need to be available; do any restrictions apply?	Not Applicable	

Antibodies	Information included in the manuscript?	In which section is the information available? (Reagents and Tools Table, Materials and Methods, Figures, Data Availability Section)
For **antibodies** provide the following information:<br>- Commercial antibodies: RRID (if possible) or supplier name, catalogue number and or/clone number<br>- Non-commercial: RRID or citation	Yes	Materials and Methods and Table 2

DNA and RNA sequences	Information included in the manuscript?	In which section is the information available? (Reagents and Tools Table, Materials and Methods, Figures, Data Availability Section)
**Short novel DNA or RNA including primers, probes**: provide the sequences.	Not Applicable	

Cell materials	Information included in the manuscript?	In which section is the information available? (Reagents and Tools Table, Materials and Methods, Figures, Data Availability Section)
**Cell lines:** Provide species information, strain. Provide accession number in repository **OR** supplier name, catalog number, clone number, and/**OR** RRID.	Not Applicable	
**Primary cultures:** Provide species, strain, sex of origin, genetic modification status.	Not Applicable	
Report if the cell lines were recently **authenticated** (e.g., by STR profiling) and tested for mycoplasma contamination.	Not Applicable	

Experimental animals	Information included in the manuscript?	In which section is the information available? (Reagents and Tools Table, Materials and Methods, Figures, Data Availability Section)
**Laboratory animals or Model organisms:** Provide species, strain, sex, age, genetic modification status. Provide accession number in repository **OR** supplier name, catalog number, clone number, **OR** RRID.	Yes	Materials and Methods
**Animal observed in or captured from the field:** Provide species, sex, and age where possible.	Not Applicable	
Please detail **housing and husbandry conditions**.	Yes	Materials and Methods

Plants and microbes	Information included in the manuscript?	In which section is the information available? (Reagents and Tools Table, Materials and Methods, Figures, Data Availability Section)
**Plants:** provide species and strain, ecotype and cultivar where relevant, unique accession number if available, and source (including location for collected wild specimens).	Not Applicable	
**Microbes:** provide species and strain, unique accession number if available, and source.	Not Applicable	

Human research participants	Information included in the manuscript?	In which section is the information available? (Reagents and Tools Table, Materials and Methods, Figures, Data Availability Section)
If collected and within the bounds of privacy constraints report on age, sex and gender or ethnicity for all study participants.	Not Applicable	

Core facilities	Information included in the manuscript?	In which section is the information available? (Reagents and Tools Table, Materials and Methods, Figures, Data Availability Section)
If your work benefited from core facilities, was their service mentioned in the acknowledgments section?	Yes	Acknowledgements

Design

Study protocol	Information included in the manuscript?	In which section is the information available? (Reagents and Tools Table, Materials and Methods, Figures, Data Availability Section)

If study protocol has been **pre-registered, provide DOI in the manuscript**. For clinical trials, provide the trial registration number **OR** cite DOI.	Not Applicable	
Report the **clinical trial registration number** (at ClinicalTrials.gov or equivalent), where applicable.	Not Applicable	

Laboratory protocol	Information included in the manuscript?	In which section is the information available? (Reagents and Tools Table, Materials and Methods, Figures, Data Availability Section)
Provide DOI OR other citation details if **external detailed step-by-step protocols** are available.	Not Applicable	

Experimental study design and statistics	Information included in the manuscript?	In which section is the information available? (Reagents and Tools Table, Materials and Methods, Figures, Data Availability Section)
Include a statement about **sample size** estimate even if no statistical methods were used.	Yes	Figure captions
Were any steps taken to minimize the effects of subjective bias when allocating animals/samples to treatment (e.g. **randomization procedure**)? If yes, have they been described?	Yes	Materials and Methods
Include a statement about **blinding** even if no blinding was done.	Yes	Materials and Methods
Describe **inclusion/exclusion criteria** if samples or animals were excluded from the analysis. Were the criteria pre-established?<br><br>If sample or data points were omitted from analysis, report if this was due to attrition or intentional exclusion and provide justification.	Yes	Materials and Methods
For every figure, are **statistical tests** justified as appropriate? Do the data meet the assumptions of the tests (e.g., normal distribution)? Describe any methods used to assess it. Is there an estimate of variation within each group of data? Is the variance similar between the groups that are being statistically compared?	Yes	Figure captions, Materials and Methods

Sample definition and in-laboratory replication	Information included in the manuscript?	In which section is the information available? (Reagents and Tools Table, Materials and Methods, Figures, Data Availability Section)
In the figure legends: state number of times the experiment was **replicated** in laboratory.	Not Applicable	
In the figure legends: define whether data describe **technical or biological replicates**.	Yes	Figure captions

Ethics

Ethics	Information included in the manuscript?	In which section is the information available? (Reagents and Tools Table, Materials and Methods, Figures, Data Availability Section)
Studies involving **human participants**: State details of **authority granting ethics approval** (IRB or equivalent committee(s), provide reference number for approval.	Not Applicable	
Studies involving **human participants**: Include a statement confirming that **informed consent** was obtained from all subjects and that the experiments conformed to the principles set out in the WMA Declaration of Helsinki and the Department of Health and Human Services Belmont Report.	Not Applicable	
Studies involving **human participants:** For publication of **patient photos**, include a statement confirming that consent to publish was obtained.	Not Applicable	
Studies involving experimental **animals**: State details of **authority granting ethics approval** (IRB or equivalent committee(s), provide reference number for approval. Include a statement of compliance with ethical regulations.	Yes	Materials and Methods
Studies involving **specimen and field samples:** State if relevant **permits** obtained, provide details of authority approving study; if none were required, explain why.	Not Applicable	

Dual Use Research of Concern (DURC)	Information included in the manuscript?	In which section is the information available? (Reagents and Tools Table, Materials and Methods, Figures, Data Availability Section)
Could your study fall under dual use research restrictions? Please check biosecurity documents and list of **select agents and toxins** (CDC): https://www.selectagents.gov/sat/list.htm	Not Applicable	
If you used a select agent, is the security level of the lab appropriate and reported in the manuscript?	Not Applicable	
If a study is subject to dual use research of concern regulations, is the name of the **authority granting approval and reference number** for the regulatory approval provided in the manuscript?	Not Applicable	

Reporting

The MDAR framework recommends adoption of discipline-specific guidelines, established and endorsed through community initiatives. Journals have their own policy about requiring specific guidelines and recommendations to complement MDAR.

Adherence to community standards	Information included in the manuscript?	In which section is the information available? (Reagents and Tools Table, Materials and Methods, Figures, Data Availability Section)
State if relevant guidelines or checklists (e.g., **ICMJE, MIBBI, ARRIVE, PRISMA**) have been followed or provided.	Not Applicable	
For **tumor marker prognostic studies,** we recommend that you follow the **REMARK** reporting guidelines (see link list at top right). See author guidelines, under 'Reporting Guidelines'. Please confirm you have followed these guidelines.	Not Applicable	
For **phase II and III randomized controlled trials**, please refer to the **CONSORT** flow diagram (see link list at top right) and submit the CONSORT checklist (see link list at top right) with your submission. See author guidelines, under 'Reporting Guidelines'. Please confirm you have submitted this list.	Not Applicable	

Data Availability

Data availability	Information included in the manuscript?	In which section is the information available? (Reagents and Tools Table, Materials and Methods, Figures, Data Availability Section)
Have **primary datasets** been deposited according to the journal's guidelines (see 'Data Deposition' section) and the respective accession numbers provided in the Data Availability Section?	Yes	Data availability section
Were **human clinical and genomic datasets** deposited in a public access-controlled repository in accordance to ethical obligations to the patients and to the applicable consent agreement?	Not Applicable	
Are **computational models** that are central and integral to a study available without restrictions in a machine-readable form? Were the relevant accession numbers or links provided?	Not Applicable	
If publicly available data were reused, provide the respective **data citations in the reference list**.	Not Applicable	
