# Supplementary material for: Adult neural stem cells and neurogenesis are resilient to intermittent fasting
Source: EMBO Rep. 2023 Nov 21;24(12):e57268. doi: 10.15252/embr.202357268 (PMC10702802; doi:10.15252/embr.202357268)
Supplement: Supplementary file 9 — Source Data for Figure 3 [file EMBR-24-e57268-s007.zip › Figure 4/4C/README.rtf]

Channels
Channel 1: NeuN
Channel 2: DCX
Channel 3: YFP
Channel 4: DAPI
